# Efficacy of *Forsythia suspensa* (Thunb.) Vahl on mouse and rat models of inflammation-related diseases: a meta-analysis

**DOI:** 10.3389/fphar.2024.1288584

**Published:** 2024-03-04

**Authors:** Chenyu Zhou, Quan Xia, Hamizah Shahirah Hamezah, Zheng Fan, Xiaohui Tong, Rongchun Han

**Affiliations:** ^1^ School of Pharmacy, Anhui University of Chinese Medicine, Hefei, China; ^2^ Department of Pharmacy, The First Affiliated Hospital of Anhui Medical University, Hefei, China; ^3^ Institute of Systems Biology, Universiti Kebangsaan Malaysia, Bangi, Malaysia; ^4^ Affiliated Taihe Hospital of Chinese Medicine, Anhui University of Chinese Medicine, Taihe, China; ^5^ School of Life Sciences, Anhui University of Chinese Medicine, Hefei, China; ^6^ Functional Activity and Resource Utilization on Edible and Medicinal Fungi Joint Laboratory of Anhui Province, Jinzhai, China; ^7^ Joint Research Center for Chinese Herbal Medicine of Anhui of IHM, Anhui University of Chinese Medicine, Hefei, China

**Keywords:** meta-analysis, forsythiae fructus, inflammatory disease, inflammatory signaling pathway, MAPK pathway

## Abstract

**Objective:** To evaluate the efficacy of the fruits of the medicinal plant *Forsythia suspensa* (Thunb.) Vahl (FS), in treating inflammation-associated diseases through a meta-analysis of animal models, and also probe deeply into the signaling pathways underlying the progression of inflammation.

**Materials and methods:** All data analyses were performed using Review Manager 5.3 and the results are presented as flow diagrams, risk-of-bias summaries, forest plots, and funnel plots. Summary estimates were calculated using a random- or fixed-effect model, depending on the value of I2.

**Results:** Of the 710 records identified in the initial search, 11 were selected for the final meta-analysis. Each study extracted data from the model and treatment groups for analysis, and the results showed that FS alleviated the inflammatory cytokine levels in serum; oxidant indicator: reactive oxygen species; enzymes of liver function; endotoxin and regulatory cells in blood; and improved the antioxidant enzyme superoxide dismutase.

**Conclusion:** FS effectively reversed the change in acute or chronic inflammation indicators in animal models, and the regulation of multiple channel proteins in inflammatory signaling pathways suggests that FS is a good potential drug for inflammatory disease drug therapy.

## 1 Introduction

Inflammation is categorized as acute or chronic. Acute inflammation is a transient normal physiological response of the body to external stimulation, injury, or infection caused by various pathogens or sterile stimuli, such as toxins, allergens, foreign bodies, and even injured host cells (Grondman et al., 2020). Irritants bind to their receptors on immune cells, triggering a signaling cascade that leads to the production of cytokines and inflammatory mediators (Halova et al., 2018). The process of inflammation is terminated when the inflamed tissue is repaired and enters a homeostatic state (Kourtzelis et al., 2020). Chronic inflammation is activated when the acute inflammatory mechanism fails to fight infection or heal injury, which induces continuous production of immune cells.

As an important defense mechanism of the body, inflammation has long been a “double-edged sword” for human health. The traditional standard view of inflammation is that it is a protective response triggered by the body to fight infections or tissue injuries. However, owing to practical needs, scientists in recent years have focused on inflammation as a vital risk factor for many chronic diseases, including hepatitis, diabetes, and cancer, and have demonstrated that inflammatory processes are closely related to the occurrence and development of aforementioned diseases (Deng and Liu, 2021). Since the advent of aspirin over a century ago, hundreds of drugs have been developed to treat various inflammatory diseases. Considering the toxicity and effectiveness of traditional medicines, researchers have focused on the application of Chinese herbal medicines for the treatment of inflammatory syndromes.

The dried fruit of *Forsythia suspensa* (Thunb.) Vahl (FS) is a crude drug named Forsythiae Fructus. Referring to the 2020 edition of the *Pharmacopoeia of the People’s Republic of China* (Commission of Chinese Pharmacopoeia, 2020), FS tastes bitter and is slightly cold. According to the meridian distribution theory of traditional Chinese medicine (TCM), it belongs to the lung, heart, and small intestine meridians and has the effect of clearing heat and detoxifying, reducing swelling, and dispersing nodules, as well as dispersing wind and heat. At present, more than 200 chemical components have been isolated from FS, which mainly include phenylethanolsides, lignans, volatile oils, and flavonoids. Several publications have reported that forsythoside A, forsythoside B, phillyrin, phillygenin, rutin, luteolin, and other components extracted from FS have anti-inflammatory activities (Feng et al., 2018). Hence, FS is often used in the clinical treatment of various inflammation-related diseases, such as anemopyretic cold, scabies, ulcers, and mastitis (Xia et al., 2016).

By further exploring the anti-inflammatory molecular mechanism of FS, researchers have applied the method of molecular docking of network pharmacology and found that several selected chemical components of FS are associated with numerous related target proteins in NF-κB and mitogen-activated protein kinase (MAPK) signaling pathways (Liu, 2021), indicating that the signaling pathways related to the anti-inflammatory effects of FS may mainly be these two pathways. Inflammation-related signaling pathways regulate the production of various enzymes and mediators during inflammation. FS exerts anti-inflammatory effects by affecting these signaling pathways *in vivo*, thereby affecting enzyme activity and blocking the production of inflammatory cytokines (Stipp and Acco, 2021).

## 2 Materials and methods

### 2.1 Data sources and searches

Studies were systematically and comprehensively retrieved using electronic databases in English, such as PubMed/Medline, and CNKI in Chinese from 2000 to 2023. Search strategy included the following key words [(“forsythia” OR forsythia OR forsythia suspensa OR fructus forsythiae OR green fructus forsythiae OR grown fructus forsythiae OR qing qiao OR lao qiao OR forsythia suspensa extract) AND (“Inflammation” OR inflammation OR inflammatory disease OR inflammatory related disease OR inflammatory associated disease OR acute inflammation OR chronic inflammation) AND (“models, animal” OR animal models OR animal OR experimental OR mice OR mouse OR rat OR rats)]. [(‘lian qiao’ OR ‘qing qiao’ OR ‘lao qiao’ NOT ‘lian qiao gan’) AND (‘yan zheng’) AND (‘xiao shu’ OR ‘dong wu mo xing')] were searched in Chinese database. Two researchers in the field of TCM pharmacology read all the included studies. The papers were double-checked and then discussed with a third researcher specializing in pharmacognosy to avoid omissions and errors.

### 2.2 Inclusion and exclusion criteria

Although the subjects of this meta-analysis were not clinical cases, we referred to the population, intervention, comparison, outcome, and study design (PICOS) principle with changes according to the specific content to list the detailed inclusion and exclusion criteria.

### 2.3 Types of studies

The studies were selected based on the following inclusion criteria: (1) animal models limited to rats and mice; (2) only English and Chinese; (3) inflammation-associated disease models, including acute or chronic inflammation caused indirectly by other diseases; (4) complete papers rather than abstracts; and (5) outcome data, including indicators of inflammatory cytokines or inflammatory pathway proteins.

Studies presented by reviews, *in vitro* studies, conference papers, and clinical trials were excluded.

### 2.4 Types of interventions

Owing to different parts of the FS plant used and various chemical extraction methods in several studies, included articles must meet the following standards referring to Chinese pharmacopoeia: (1) dried fruit as medicinal part (qingqiao or laoqiao); (2) aqueous extract, water decoction, or freeze-dried powder of FS; (3) only FS treatment during intervention; (4) oral administration for intervention; (5) noting the basis for administration dose; and (6) detailed protocol for drug preparation.

The FS used in these studies was derived from *F. suspensa*. One of the active ingredients extracted from FS as a single drug in the experiments–phillyrin, phillygenin, or forsythiaside A–was excluded. Formulas containing FS or combinations were not considered.

### 2.5 Data collection

Two reviewers, Zhou and Tong, reviewed the 11 included studies, screened the necessary information, and listed the tables upon mutual agreement through discussion. Since the triggers that cause inflammation are different and the resultant outcomes are diverse, the selected data were included in our analysis using software Review Manager 5.3: proinflammatory cytokines (TNF-α, IL-6, and IL-1β) in serum, alanine aminotransferase (ALT), aspartate aminotransferase (AST), reactive oxygen species (ROS), endotoxin (ET), and regulatory cells (Treg). Considering disparate drug concentrations in the included experiments, that is, some had only one dose, whereas others had three doses (low, medium, and high), we used the following practice in this study: if there were two or more drug concentrations in an experiment, the control group was divided into the new corresponding control groups, with which different concentration groups were combined for comparison. Missing data that were not specifically presented in the papers were requested by contacting the corresponding or first author.

### 2.6 Risk-of-bias assessment and publication bias

**FIGURE 1:**
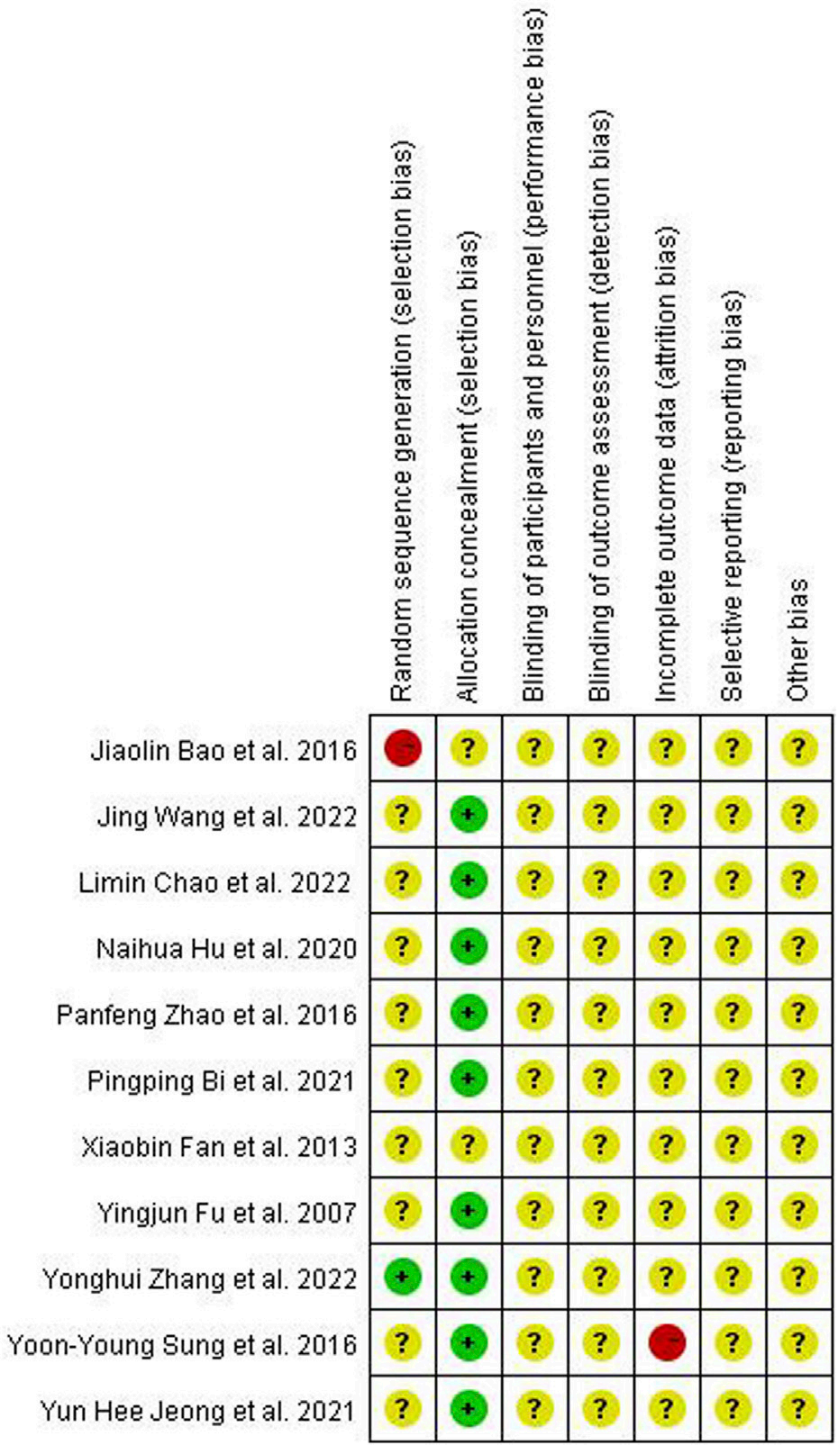
Risk of bias summary.

Quality assessment of the included studies was completed using Revman software (version 5.3). Two reviewers used the randomized controlled trials (RCTs) quality evaluation criteria in the Cochrane Manual of Systematic Reviewers to evaluate the quality of each included methodology as follows: (1) selection bias: random sequence generation, allocation concealment; (2) performance bias: blinding of participants and personnel; (3) detection bias: blinding of outcome assessment; (4) attrition bias: incomplete outcome data; (5) reporting bias: selective reporting; and (6) other biases. Each assessment result was represented by three risk degrees–low, unclear, or high risk–in terms of the risk offset that may occur (Figure 1).

Among the 11 articles, only Zhang et al. (Zhang et al., 2022) employed the double-blind method to group experimental animals, which was evaluated as “low risk” in selection bias. Conversely, Bao et al. (Bao et al., 2016) did not explain the grouping method in detail and was, therefore, classified as “high risk.”

**FIGURE 2:**
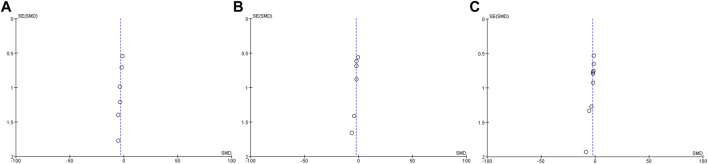
The funnel plot of **(A)**. TNF-α, **(B)**. IL-6, **(C)**. IL-1β.

A graphical funnel plot was used to investigate the presence of publication bias in the studies, including the main outcomes (Figure 2).

### 2.7 Statistical method

Meta-analysis was performed using Revman (version 5.3). Owing to the diversity among the experimental animals in terms of sex, breed, and weight, standard mean difference (SMD) and 95% confidence interval (CI) were used as effect analysis statistics for the measurement data. The I^2^ test was used as the indicator of heterogeneity test in the analysis. *p* > 0.1 and I^2^ < 50% indicated that the results of included studies were homogenous and the fixed-effects model was used for meta-analysis, and if *p* ≤ 0.1 and I^2^ ≥ 50%, the random-effects model was used on account of great statistical heterogeneity among the results. Statistical significance was set at *p* < 0.05.

## 3 Results

### 3.1 Study selection

**FIGURE 3:**
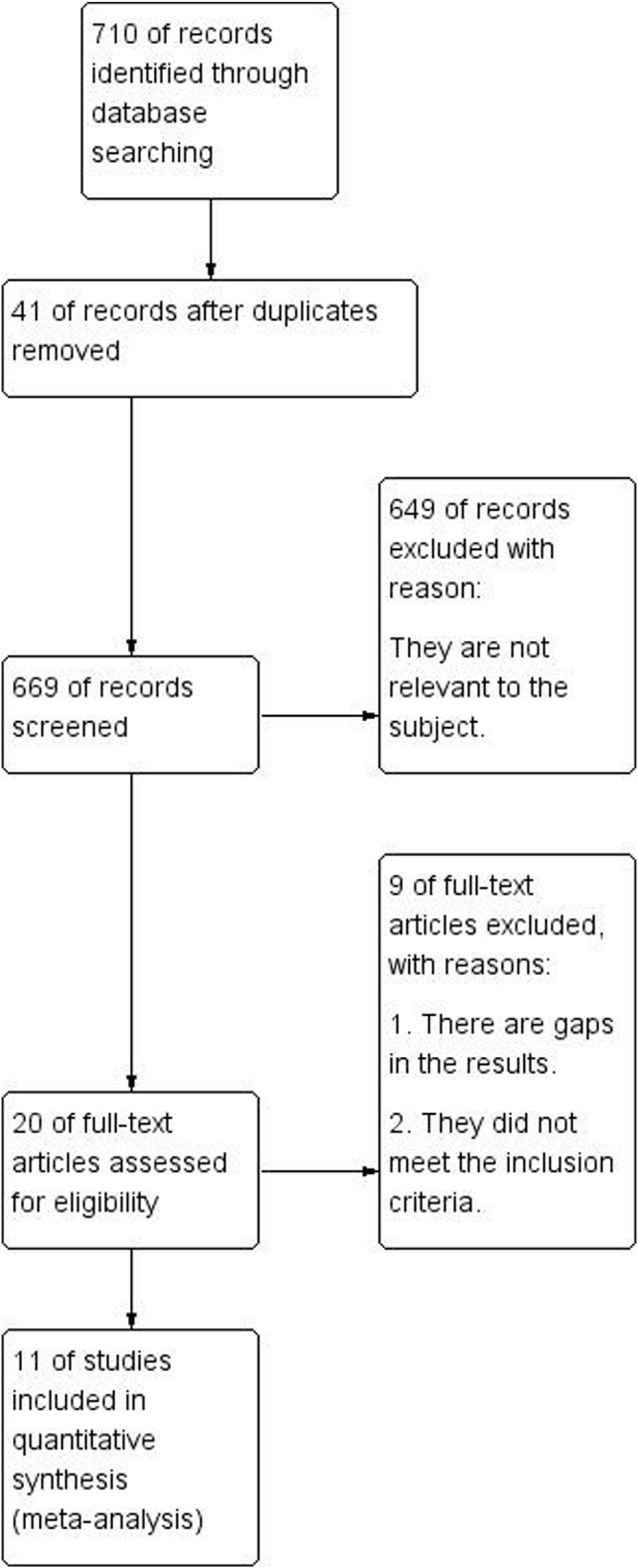
Flow diagram.

As illustrated in Figure 3, 710 articles were retrieved from Chinese and English databases through a keyword search, and 42 were duplicates. Of the remaining 669 essays, 649 were eliminated after title and abstract perusal, leaving 20 eligible studies for full-text review. Through simultaneous screening by two reviewers, the experimental materials or methods of seven studies were found to not fulfill the inclusion criteria, and two showed insufficient data. In summary, 11 articles were eventually included in the quantitative synthesis.

### 3.2 Characteristics of included studies

There were 442 experimental animals in the 11 included articles. Because of the different disease models involved, the following species were used: ICR mice, SD rats, NC/Nga mice, Wistar rats, and C57BL/6 mice. Except for two studies that used female mice and one study that used half males and half females, all subjects were male. Despite the various administration dosages for each experimental design (Table 1), the method for drug intervention was by gavage. The duration of the drug intervention depended on the severity of inflammation. Fulminant hepatitis, burn injury, and acute organ injury associated with acute inflammation had generally 3–10 days of intervention period, whereas treatment for chronic inflammation, such as liver fibrosis and atopic dermatitis, lasted at least 2 weeks and even 8 weeks for tumors. Statistical heterogeneity was apparent for most continuous variables. The main outcomes suggested no significant publication bias.

**TABLE 1:** Summary of included studies.

Study	Animal model/species	Sex	Age (weeks)	Weight (g)	N per group	Dose or concentration	Duration of intervention (days)	Outcome
Zhang et al. (2022)	Chronic pelvic inflammatory model rats/SD	F	12–13	219.7 ± 8.5	10	250 mg/kg body weight	/	IgG, IgA, IgM
Jeong et al. (2021)	Fulminant hepatitis mice/ICR	M	6	27–33	9	Med: FS 100 mg/kg Hi: FS 300 mg/kg	6	serum TNF-α, IL-6, IL-1β, AST, ALT, ALP
Sung et al. (2016)	Murine atopic dermatitis mice/NC/Nga	M	8	/	6	500 µg/mouse	14	serum IgE, IL-4
Zhao et al. (2016)	Liver injury rats/SD	M	/	75–90	8	100 mg/kg body weight	7	serum TNF-α, IL-6, IL-1β, AST, ALT, ROS, SOD
Hu et al. (2020)	Rats with liver fibrosis/SD	MF	/	180–220	10	2.5 g/kg body weight 5 g/kg body weight	56	serum TNF-α, IL-6, IL-1β, ALT, AST, AKP
Bao et al. (2016)	B16 melanoma mice/C57BL/6	F	/	/	6	5 g/kg body weight 10 g/kg body weight LFS: 0.75 g/kg body weight	32	TNF-α, IL-6, ROS, MDA, GSH
Wang et al. (2022)	LPS-induced acute lung injury mice/ICR	M	5–6	18–22	5	MFS: 1.5 g/kg body weight HFS: 3 g/kg body weight	3	mRNA IL-6, IL-1β, TNF-α
Bi (2021)	Chemotherapy induced-nausea and vomiting rats/Wistar	M	/	180–220	6	1.75 g/kg body weight	6	serum ROS, IL-1β, IL-18; ASC, caspase-1
Chao et al. (2022)	Ulcerative colitis mice/C57BL/6J	M	7	/	10	LFS: 0.1 g/mL	/	serum IL-1β; HO-1, ASC, caspase-1
						MFS: 0.2 g/mL		
						HFS: 0.4 g/mL		
LFS: 1.25 g/kg								
Fu (2007)	Burnt rats/SD	M	6–8	150–200	8	MFS: 2.5 g/kg	10	Treg, ET
						HFS: 5 g/kg		
Fan et al. (2013)	Severe acute pancreatitis rats/Wistar	M	/	230–270	8	LFS: 1.25 g/kg	7	AMY, ALT, ET, Treg
						MFS: 2.5 g/kg		
						HFS: 5 g/kg		

### 3.3 Outcome analysis

#### 3.3.1 Serum inflammatory factors

**FIGURE 4:**
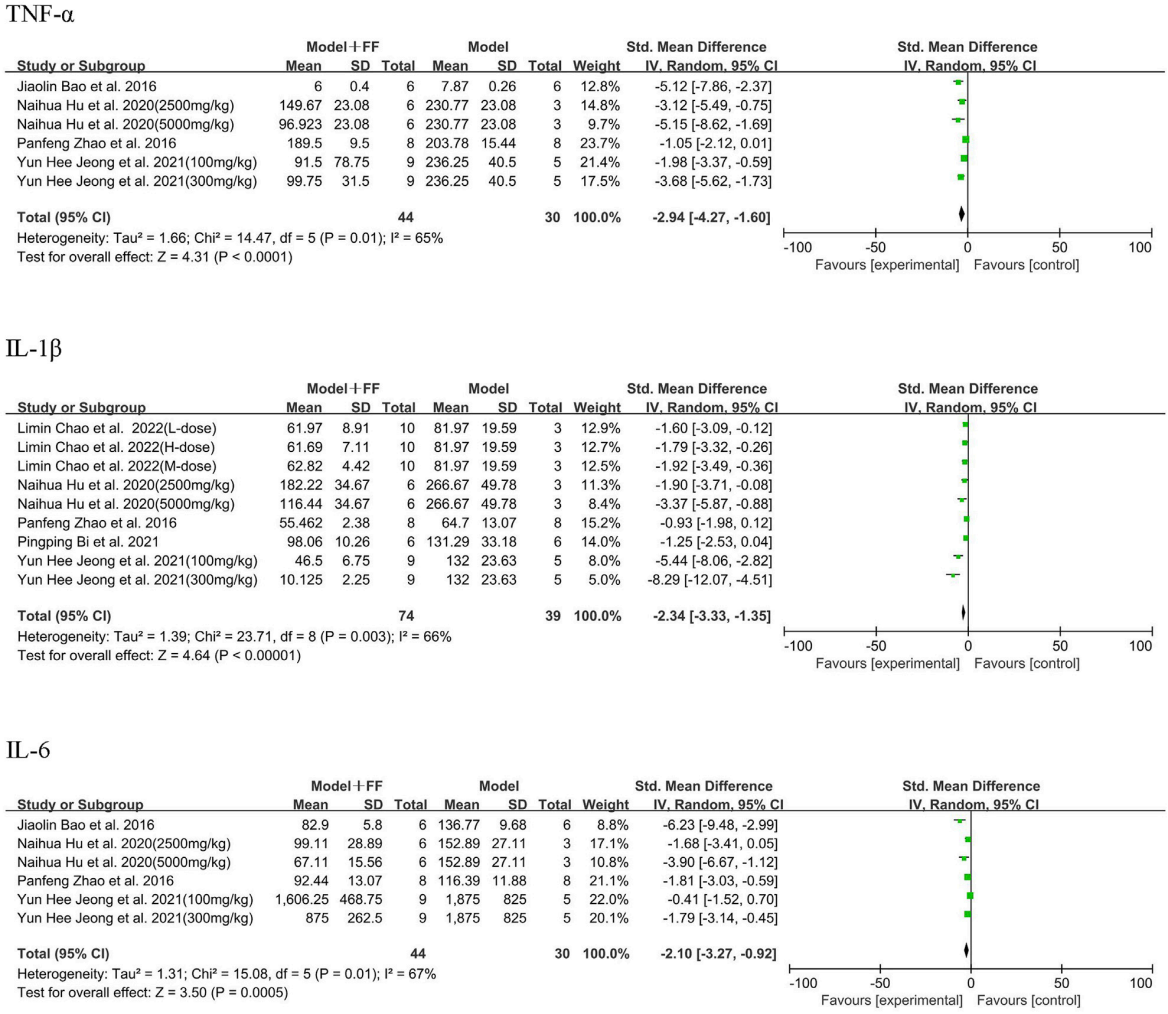
Inflammatory factors in serum.

As the main outcomes, TNF-α, IL-1β, and IL-6 were measured in the blood serum by six studies, which applied 10 concentrations of FS intervention (Figure 4). Regarding four studies ((Jeong et al., 2021; Zhao et al., 2016; Hu et al., 2020; Bao et al., 2016)) that evaluated TNF-α, the results indicated significant heterogeneity among them (*p* = 0.01, I^2^ = 65%). Using random-effects model, meta-analysis illustrated that the level of TNF-α was dramatically decreased (SMD -2.94, 95% CI -4.27–1.6, and *p* < 0.0001). Regarding the five studies [(Jeong et al., 2021; Zhao et al., 2016; Hu et al., 2020; Bi, 2021; Chao et al., 2022)], FS significantly reduced IL-1β levels compared with the model group (SMD -2.34, 95% CI -3.33–1.35, and *p* < 0.00001, and heterogeneity *p* = 0.003, I^2^ = 66%). Considering IL-6 in four studies [(Jeong et al., 2021; Zhao et al., 2016; Hu et al., 2020; Bao et al., 2016)], the outcome data showed the level in Model + FS group was lower than that in the Model group (SMD -2.1, 95% CI -3.27–0.92, *p* = 0.0005) resulted from random-effects model due to heterogeneity (*p* = 0.01, I2 = 67%).

#### 3.3.2 Anti-oxidation effect

**FIGURE 5:**
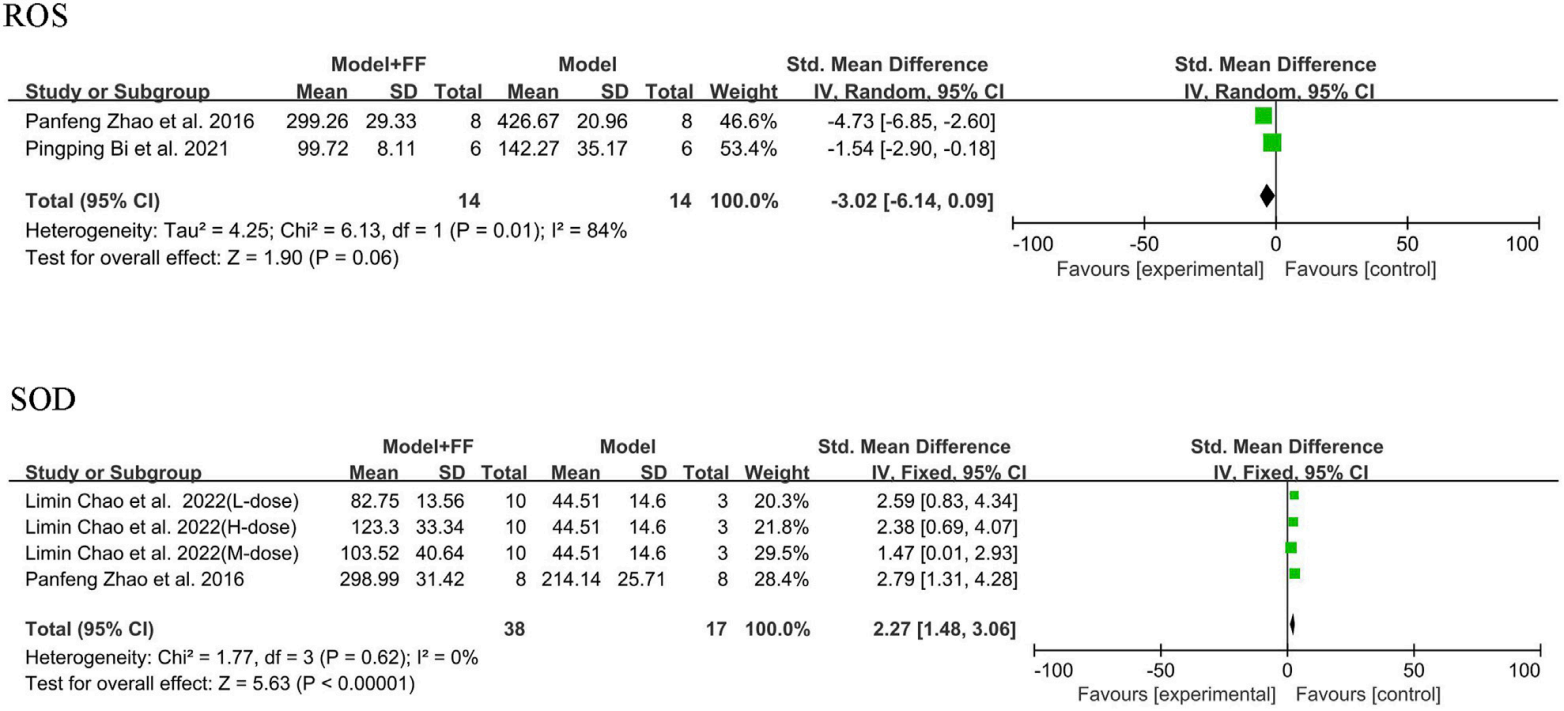
The level of ROS and SOD.

Alleviation of oxidative stress by FS in an inflammatory mouse model was determined by assessing ROS and the antioxidant enzyme superoxide dismutase (SOD). Only two studies ((Zhang et al., 2022; Bi, 2021)) using the random-effects model mentioned ROS, and the analysis results suggested significant heterogeneity (*p* = 0.01, I^2^ = 84%). Although the sample size was small in only two groups, a reduction in ROS in the mouse blood was still evident (SMD -3.02, 95% CI -6.14–0.09, and *p* = 0.06). Under fixed-effects model, two studies [(Zhang et al., 2022; Chao et al., 2022)] showed that the heterogeneity of SOD outcome was not significant (*p* = 0.62, I^2^ = 0%), with a clear elevation in SOD level in mice blood (SMD 2.27, 95% CI 1.48–3.06, and *p* < 0.00001; Figure 5).

#### 3.3.3 Liver parameters

**FIGURE 6:**
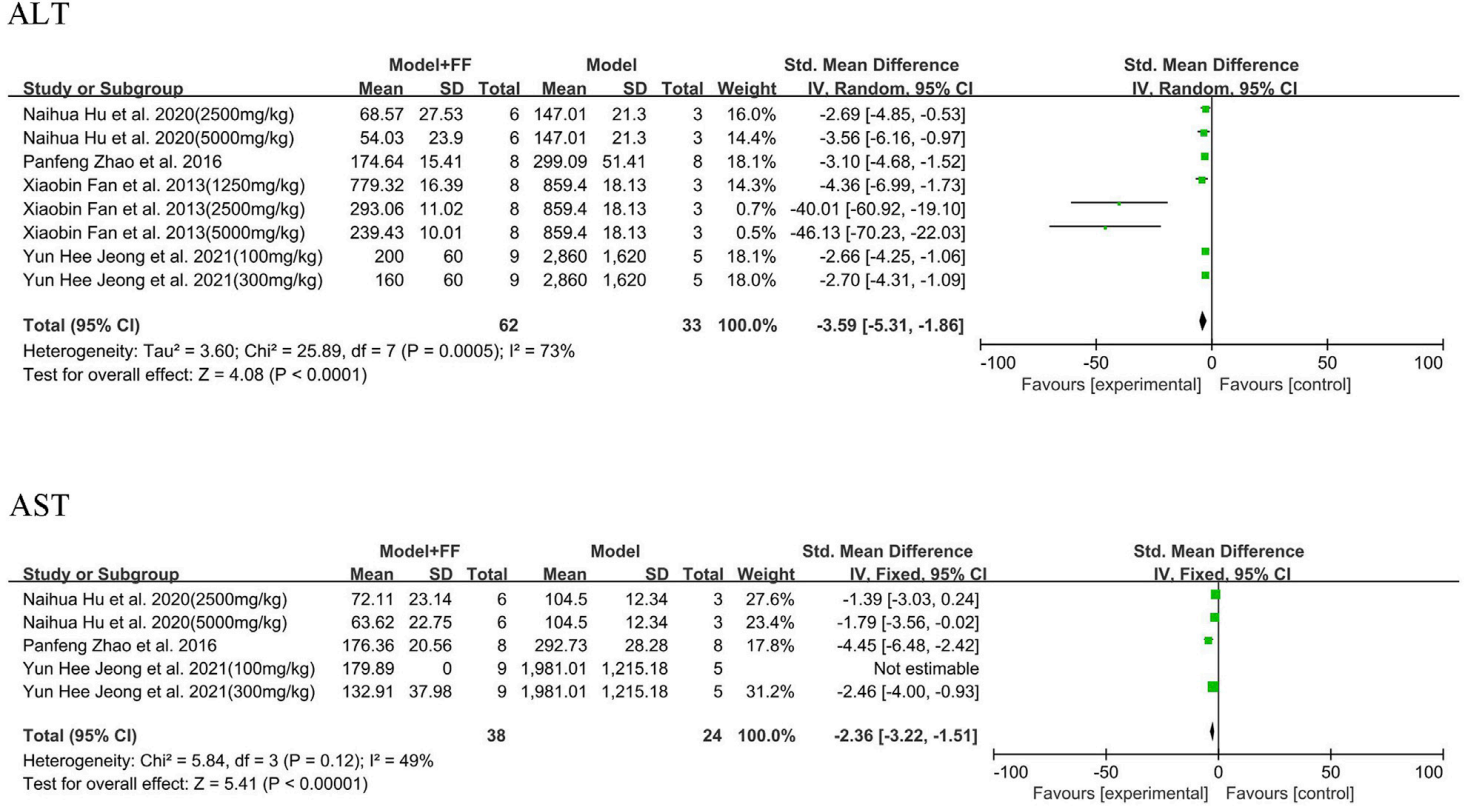
Enzymes of liver function.

Serum ALT and AST outcomes for hepatocytes and liver injury are illustrated in Figure 5 and summarized in four studies [(Zhang et al., 2022; Jeong et al., 2021; Hu et al., 2020; Fan et al., 2013)] that showed significant heterogeneity (*p* = 0.0005, I^2^ = 73%; Figure 6). A random-effects model was selected for analysis, which found that ALT substantially decreased in treatment group (SMD -3.59, 95% CI -5.31–1.86, and *p* < 0.0001). In three studies ((Zhang et al., 2022; Jeong et al., 2021; Hu et al., 2020)), the levels of AST in serum were decreasing after FS intervention compared with model groups (SMD -2.36, 95% CI -3.22–1.51, and *p* < 0.00001), whereas the heterogeneity was not significant (*p* = 0.12, I^2^ = 49%). Based on the value of I^2^, a fixed-effects model was used in the meta-analysis.

#### 3.3.4 Blood parameters

**FIGURE 7:**
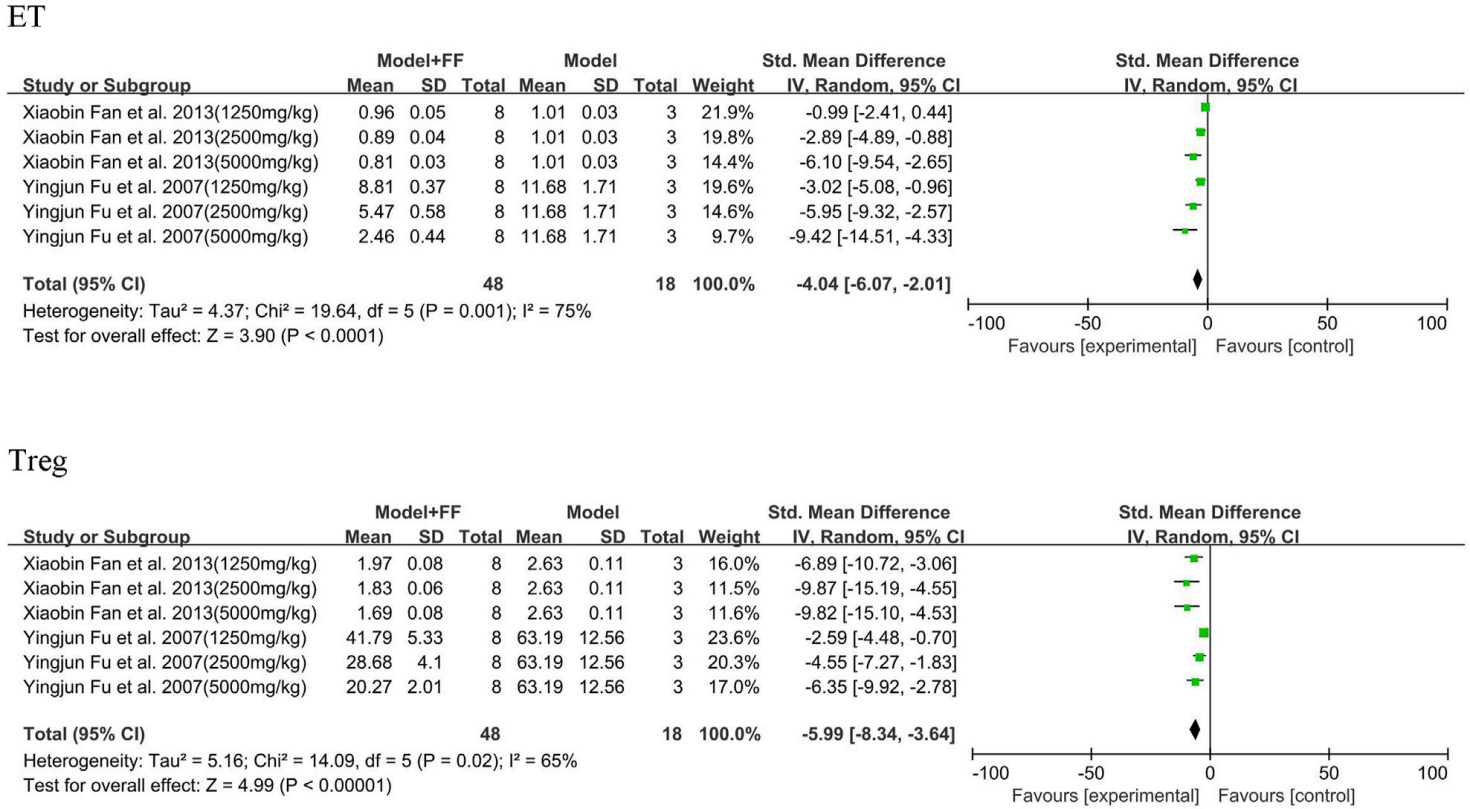
Blood parameters.

Under acute inflammatory conditions, the expression of ET and Treg (%) in peripheral blood sharply increased (Figure 7). In two studies ((Fu, 2007; Fan et al., 2013)), FS decoction in three concentrations (1250, 2500, and 5000 mg/kg) played a key part in decreasing these two indicators in blood. The ET analysis results demonstrated significant heterogeneity (*p* = 0.001, I^2^ = 75%), and significant statistical differences were observed between the model and treated groups (SMD -4.04, 95% CI -6.07–2.01, and *p* < 0.0001). Heterogeneity of Treg analysis was not as significant as that of ET (*p* = 0.02, I^2^ = 65%); however, the concentration of Treg in peripheral blood was also decreased (SMD -5.99, 95% CI -8.34–3.64, and *p* < 0.00001).

### 3.4 Inflammatory cytokines and expression of inflammation-related genes and proteins

Based on the findings in Section 3.3.1, not only was there a sharp reduction of cytokine TNF-α and IL-6 levels in serum after FS intervention, but also a decrease of *TNF-*α and *IL-6* mRNA levels was observed in the liver, lungs, and colon according to relevant studies [(Jeong et al., 2021; Hu et al., 2020; Bi, 2021)]. In addition to reducing serum IL-1β levels, FS also downregulated expressions of *IL-1*β mRNA in the liver, lungs, and colon tissue in four studies [(Jeong et al., 2021; Hu et al., 2020; Wang et al., 2022; Chao et al., 2022)]. By reporting the decreased IL-18 concentration in rat serum after FS treatment, Bi (Bi, 2021) showed that FS significantly reduced the expression levels of NLRP3, caspase-1, and IL-1β in gastrointestinal tissues of rats who underwent chemotherapy. Moreover, Chao et al. Chao et al. (2022) concluded that FS might exert anti-inflammatory effects by mediating pyroptosis through the Nrf-NLRP3 pathway.

Two studies (Zhang et al., 2022; Jeong et al., 2021) related to hepatic diseases showed that FS intervention inhibited NF-κB pathway and upregulated Nrf2/Nrf2-HO-1 pathway. Fan et al. (Fan et al., 2013) reported that FS lowered the expressions of *NF-κB* and *Foxp3* mRNAs. The effect of downregulating *Foxp3* mRNA and protein expression was also illustrated in another study (Fu, 2007). Zhang et al. (Zhang et al., 2022) reported that FS reduced the ICAM-1, TLR4, and NF-κB levels while simultaneously increasing the level of INF-γ. In liver fibrosis model, Hu et al. (Jeong et al., 2021) showed that FS upregulated the expression of TGF-β1, p-smad2, and p-smad3 and lowered smad7 related to the TGF-β/smads signaling pathway. They also reported that TLR4/MyD88/NF-κB signaling pathway-related proteins MyD88, TAK1, p-p65, and p-IκBα were significantly decreased compared with model group. Bao et al. (Bao et al., 2016) demonstrated the anti-tumor activity of FS through activation of the MAPK signaling pathway and upregulation of Nrf2/HO-1.

## 4 Discussion

The occurrence of disease and inflammation is reciprocal, which means that inflammation is an inevitable consequence of several diseases, and chronic inflammation may trigger autoimmune diseases and drive cancer (Singhal and Kumar, 2022). Roe summarized and classified different types of diseases that induce inflammation and regulate cytokines. In addition to the known causes, such as pathogen infection and physical injury, several chronic conditions are also probably accompanied by inflammation (Roe, 2020). In various animal experiments, FS intervention not only reduced the levels of related inflammatory indicators, but also intuitively changed the morphology of the damaged site and alleviated the condition.

### 4.1 Hepatic diseases

The liver is an important target organ for inclusion in disease models in meta-analyses. High concentrations of aminotransferases are present in the liver, and the activity of various enzymes indicates liver injury. When hepatic parenchymal cells are damaged, sensitive cytoplasmic markers, such as ALT and AST, are released into the blood (Morán-Salvador et al., 2013). As early as the 20th century, clinical findings on the use of FS in the treatment of acute hepatitis concluded that FS significantly reduced the activity of serum ALT and ameliorated liver degeneration and necrosis. Based on this, researchers have used animal models of acute liver injury to show that oleanolic and ursolic acids in FS may affect enzymes (Liu and Yang, 1988). However, the inflammatory factors and molecular mechanisms involved in this process have not been thoroughly studied.

As an indispensable component regulating lipid metabolism, the liver is involved in a series of processes such as lipid absorption, digestion, transportation, and decomposition (Kalish et al., 2015). The accumulation of lipids in cells results in mitochondrial dysfunction, increased oxidative stress in the endoplasmic reticulum, and other changes in organelle function that promote the formation of an inflammatory environment (van Dierendonck et al., 2022). When lipid peroxidation alters cell membrane permeability and leads to hepatocyte death, the level of ROS in the serum and liver increases rapidly, while the activity of antioxidant enzymes such as SOD decreases simultaneously (Menezes et al., 2023). Overproduction of ROS turns on the redox sensitive transcription factor NF-κB, and subsequently triggers an inflammatory response, releasing cytokines such as IL-6, IL-1β, and TNF-α. The powerful antioxidant capacity of FS was demonstrated by the reversal of oxidative injuries expressed in related indicator tests. Elevated expression of inflammatory cytokines, such as IL-6, IL-8, and TNF-α, induce insulin resistance, which contributes to the development of fatty hepatitis (Fang et al., 2022). In addition, when hepatocytes are damaged by the hepatitis B virus, inflammation- and fibrosis-related mediators are released, triggering the activation of hepatic stellate cells (HSC) and producing extracellular matrix and pro-inflammatory mediators (Henderson et al., 2020). In the case of chronic liver injury, activated HSC promote immune cells recruitment and cytokines secretion, such as TGF-β, as well as a crucial protein in fibrosis disease-related pathways, thus promoting the progression of liver fibrosis and cirrhosis (Protopsaltis et al., 2019). After being treated with FS (2.5 and 5 g/kg), mRNA expression of *TGF-β1* decreased significantly, suggesting that FS effectively alleviates liver fibrosis in mice. As mentioned in several studies, the components showing bioactivity in FS detected by HPLC were mainly forsythiaside A, forsythin, phillygenin, and pinoresinol. Furthermore, forsythoside A is the most abundant constituent (4.54%) in FS (Hu et al., 2020) and can be a candidate drug for the prevention and treatment of liver injury or inflammation-related diseases.

### 4.2 Chronic inflammation and cancer

A similar causal relationship exists between inflammation and cancer. The microenvironment in which malignant tumors grow often lacks sufficient oxygen and nutrients to keep pace with their rapid growth. In this case, numerous inflammatory cells are recruited by the accelerated release of inflammatory mediators, which promote neovascularization and provide additional growth factors for the surviving tumor cells (Protopsaltis et al., 2019; Greten and Grivennikov, 2019). The classical nuclear transcription factor NF-κB, which regulates several inflammatory cytokines, plays a key role in this process. NF-κB has been shown to participate in tumor angiogenesis, induction of anti-apoptotic gene expression to prevent tumor cell apoptosis, and activation of epithelial cell growth to promote tumor metastasis (Kaltschmidt et al., 2018).

Another signaling pathway involved in inflammation is the MAPK pathway, which is responsible for relaying extracellular stimulation to intracellular responses and has three subfamilies: ERK1/2, JNKs, and p38 (Lee et al., 2020). In the model of LPS-induced inflammation, MAPK/NF-κB pathway was activated and resulted in upregulated expression of MAPKs-related phosphorylated proteins. Western blotting revealed that FS significantly repressed the expression of these three MAPK proteins. Bao et al. (Bao et al., 2016) tested the anti-tumor activity of FS *in vivo* using a mouse allograft tumor model and showed that FS significantly inhibited the proliferation of melanoma cells and growth of melanoma in C57BL/6 mice. The anti-tumor activity of FS may be due to the upregulation of Nrf2/HO-1 through the activation of MAPK pathways, thereby reducing oxidative stress and inflammation, and inhibiting tumor cell proliferation and angiogenesis. p38 MAPK also plays a key role in tumor immunity. p38 regulates T cell activation by selectively activating the nuclear factor of activated T cells (Gurusamy et al., 2020) and is also important for Tregs that are induced by the tumor microenvironment. Tregs inhibit autoreactive T cells, thus limiting the efficacy of tumor immunotherapy, indicating that p38 inhibition may be a potential therapeutic strategy.

Remarkably, in tumor tissues, FS abates tumorigenesis by activating the MAPK pathway. It can be speculated that FS relieves inflammatory reactions in organs and tissues by inhibiting MAPK, while promoting the apoptosis of cancer cells by activating MAPK. The underlying molecular mechanisms warrant further investigation. Owing to the pivotal role of the MAPK cascade in cancer, various ERK/JNK/p38 and its upstream factor inhibitors can be used for cancer treatment, and new drugs are constantly being developed for clinical treatment.

## 5 Conclusion and limitation

The results of this meta-analysis shows that FS is effective in decreasing proinflammatory factors and can improve the microenvironment in animal models experiencing inflammation. Referring to the 11 included studies, FS can alleviate acute inflammatory injury in the liver, lungs, and colon; improve oxidative stress; have a significant effect on treating chronic pelvic inflammatory disease and atopic dermatitis; and inhibit B16 melanoma growth.

However, considering the quantity and quality of the included studies, this article has many limitations: (1) Only Chinese and English literature was included, and results in other languages are missing; (2) due to the insufficient number of included articles, no subgroup analysis was conducted based on the type of animal model, intervention period, and other factors; (3) apart from inflammatory factors, few meaningful outcome data were included in the analysis; and (4) significant statistical heterogeneity exists in the analysis, which may be due to the various animal models, poor methodological quality, or differences in treatment dose;

A change in perspective is precisely because the outcomes are cluttered by the included studies that we need to conduct a systematic meta-analysis to identify the core efficacy and indicators. In the process of retrieving, we also found three reviews on the pharmacological effects of FS (Wang et al., 2018; Zhang et al., 2024; Zhou et al., 2022). Although three publications all generalized the anti-inflammatory effects of FS, they primarily focused on the compounds derived from *F. suspensa*, with no more than two cited articles in each review describing the anti-inflammatory effects of FS. Furthermore, the types of inflammatory models were limited, most of which were *in vitro* models, and only neuroinflammation, HSV-1 virus and allergic inflammation models were mentioned *in vivo*. Our article serves to complement and update the study status of FS as a therapeutic agent for anti-inflammation *in vivo*. From the perspective of published reviews, the lack of publications systematically analyzing the anti-inflammatory effects of FS from biochemical indicators such as AST, ALT, ROS, Treg, etc. and other specific data including inflammatory cytokines in serum or tissue needs to be addressed. A meta-analysis screens all high-quality studies, and conducts combined quantitative analysis of data including inflammatory factors, antioxidant indicators, liver function parameters and blood parameters through statistical methods, objectively evaluating the anti-inflammatory effects of FS. Additionally, clear statistical charts allow readers to quickly find the required information. Different kinds of animal models have been established to study inflammation related diseases, such as mice, rats, rabbits, zebrafish, etc. However, during the collecting process, we collected only two publications assessing anti-inflammatory effects of compounds extracted from *F. suspensa* utilizing zebrafish model (Yang et al., 2017; Gong et al., 2020), and no relevant studies adopting a rabbit model were retrieved. Therefore, types of animal models in this paper were limited to mice and rats. Compared to the well-known Chinese medicines worldwide, such as ginseng, studies on the pharmacological effects of FS are not very extensive; in particular, the English literature that can be searched is limited. The authors believe that with the increasing attention of the world onto TCM, an increasing number of studies on FS will be included in meta-analyses in the future, providing novel evidence for the treatment of inflammation-related diseases, including cancer.

## Data Availability

The original contributions presented in the study are included in the article/Supplementary Material, further inquiries can be directed to the corresponding authors.

## References

[B1] Bao J. Ding R. Zou L. Zhang C. Wang K. Liu F. (2016). Forsythiae Fructus inhibits B16 melanoma growth involving MAPKs/Nrf2/HO-1 mediated anti-oxidation and anti-inflammation. Am. J. Chin. Med. 44 (5), 1043–1061. 10.1142/S0192415X16500580 27430915

[B2] Bi P. (2021). Study on the Mechanism of Forsythiae Fructus in the Prevention and Treatment of Chemotherapy-Induced Nausea and Vomiting Based on Nlrp3 Inflammasome. Guangdong Pharm. Univ. Guangzhou, China. 10.27690/d.cnki.ggdyk.2021.000033

[B3] Chao L. Lin J. Zhou J. Du H. Chen X. Liu M. (2022). Polyphenol rich *Forsythia suspensa* extract alleviates DSS-Induced ulcerative colitis in mice through the Nrf2-NLRP3 pathway. Antioxidants (Basel). 11 (3), 475. 10.3390/antiox11030475 35326124 PMC8944444

[B4] Commission of Chinese Pharmacopoeia (2020). Pharmacopoeia of the peoples Republic of China. Beijing, China: China Medical Science Press,

[B5] Deng Z. Liu S. (2021). Inflammation-responsive delivery systems for the treatment of chronic inflammatory diseases. Drug Deliv. Transl. Res. 11 (4), 1475–1497. 10.1007/s13346-021-00977-8 33860447 PMC8048351

[B6] Fan X. Li W. Chen B. Xiong Z. Duan J. (2013). Effects of Forsythia Suspensa on Expression of Nf-Κb and Foxp3 During Liver Injury in Rats with Severe Acute Pancreatitis. Second Hosp. Shanxi Med. Univ. Shanxi, China

[B7] Fang Z. Wei L. Lv Y. Wang T. Hamezah H. S. Han R. (2022). Phillyrin restores metabolic disorders in mice fed with high-fat diet through inhibition of interleukin-6-mediated basal lipolysis. Front. Nutr. 9, 956218. 10.3389/fnut.2022.956218 36276810 PMC9581271

[B8] Feng Z. Gao X. Han Y. Wang F. Zhou S. Jiang Y. (2018). Study progress of. Forsythia Suspensa. Mod. Agric. Sci. Technol. 12, 60–62.

[B9] Fu Y. (2007). Changes of Tlr4 and Treg in Severely Burnt Rats and Effects Of Forsythia Suspensa On Them. Nanchang Univ. Nanchang, China

[B10] Gong L. Yu L. Gong X. Wang C. Hu N. Dai X. (2020). Exploration of anti-inflammatory mechanism of forsythiaside A and forsythiaside B in CuSO_4_-induced inflammation in zebrafish by metabolomic and proteomic analyses. J. Neuroinflammation. 17 (1), 173. 10.1186/s12974-020-01855-9 173 32493433 PMC7271515

[B11] Greten F. R. Grivennikov S. I. (2019). Inflammation and cancer: triggers, mechanisms, and consequences. Immunity 51 (1), 27–41. 10.1016/j.immuni.2019.06.025 31315034 PMC6831096

[B12] Grondman I. Pirvu A. Riza A. Ioana M. Netea M. G. (2020). Biomarkers of inflammation and the etiology of sepsis. Biochem. Soc. Trans. 48 (1), 1–14. 10.1042/BST20190029 32049312

[B13] Gurusamy D. Henning A. N. Yamamoto T. N. Yu Z. Zacharakis N. Krishna S. (2020). Multi-phenotype CRISPR-Cas9 screen identifies p38 kinase as a target for adoptive immunotherapies. Cancer Cell. 37 (6), 818–833. 10.1016/j.ccell.2020.05.004 32516591 PMC10323690

[B14] Halova I. Rönnberg E. Draberova L. Vliagoftis H. Nilsson G. P. Draber P. (2018). Changing the threshold-signals and mechanisms of mast cell priming. Immunol. Rev. 282 (1), 73–86. 10.1111/imr.12625 29431203

[B15] Henderson N. C. Rieder F. Wynn T. A. (2020). Fibrosis: from mechanisms to medicines. Nature 587 (7835), 555–566. 10.1038/s41586-020-2938-9 33239795 PMC8034822

[B16] Hu N. Guo C. Dai X. Wang C. Gong L. Yu L. (2020). Forsythiae Fructuse water extract attenuates liver fibrosis via TLR4/MyD88/NF-κB and TGF-β/smads signaling pathways. J. Ethnopharmacol. 262, 113275. 10.1016/j.jep.2020.113275 32810620

[B17] Jeong Y. H. Hwang Y. H. Kim T. I. Oh Y. C. Ma J. Y. (2021). Forsythia Fruit prevents fulminant hepatitis in mice and ameliorates inflammation in murine macrophages. Nutrients 13 (8), 2901. 10.3390/nu13082901 2901 34445058 PMC8399229

[B18] Kalish B. T. Fell G. L. Nandivada P. Puder M. (2015). Clinically relevant mechanisms of lipid synthesis, transport, and storage. JPEN J. Parenter. Enter. Nutr. 39, 8S-17S–17S. 10.1177/0148607115595974 26187937

[B19] Kaltschmidt B. Greiner J. F. W. Kadhim H. M. Kaltschmidt C. (2018). Subunit-specific role of NF-κB in cancer. Biomedicines 6 (2), 44. 10.3390/biomedicines6020044 44 29673141 PMC6027219

[B20] Kourtzelis I. Hajishengallis G. Chavakis T. (2020). Phagocytosis of apoptotic cells in resolution of inflammation. Front. Immunol. 11, 553. 10.3389/fimmu.2020.00553 32296442 PMC7137555

[B21] Lee S. Rauch J. Kolch W. (2020). Targeting MAPK signaling in cancer: mechanisms of drug resistance and sensitivity. Int. J. Mol. Sci. 21 (3), 1102. 10.3390/ijms21031102 1102 32046099 PMC7037308

[B22] Liu W. (2021). Study on anti-inflammatory mechanism of Forsythiae Fructus based on network pharmacology. J. Shanxi Univ. 46 (1), 208–219. 10.13451/j.sxu.ns.2021038

[B23] Liu Z. Yang D. (1988). Study on the Effective Component of Forsythiae Fructus to the Liver Injury. Shanxi Med. Coll. Taiyuan, China.

[B24] Menezes L. B. Segat B. B. Tolentino H. Pires D. C. Mattos L. M. M. Hottum H. M. (2023). ROS scavenging of SOD/CAT mimics probed by EPR and reduction of lipid peroxidation in *S. cerevisiae* and mouse liver, under severe hydroxyl radical stress condition. J. Inorg. Biochem. 239, 112062. 10.1016/j.jinorgbio.2022.112062 36403436

[B25] Morán-Salvador E. Titos E. Rius B. González-Périz A. García-Alonso V. López-Vicario C. (2013). Cell-specific PPARγ deficiency establishes anti-inflammatory and anti-fibrogenic properties for this nuclear receptor in non-parenchymal liver cells. J. Hepatol. 59, 1045–1053. 10.1016/j.jhep.2013.06.023 23831119

[B26] Protopsaltis N. J. Liang W. Nudleman E. Ferrara N. (2019). Interleukin-22 promotes tumor angiogenesis. Angiogenesis 22 (2), 311–323. 10.1007/s10456-018-9658-x 30539314

[B27] Roe K. (2020). An inflammation classification system using cytokine parameters. Scand. J. Immunol. 93 (2), e12970. 10.1111/sji.12970 32892387

[B28] Singhal A. Kumar S. (2022). Neutrophil and remnant clearance in immunity and inflammation. Immunology 165 (1), 22–43. 10.1111/imm.13423 34704249

[B29] Stipp M. C. Acco A. (2021). Involvement of cytochrome P450 enzymes in inflammation and cancer: a review. Cancer Chemother. Pharmacol. 87 (3), 295–309. 10.1007/s00280-020-04181-2 33112969

[B30] Sung Y. Y. Lee A. Y. Kim H. K. (2016). *Forsythia suspensa* fruit extracts and the constituent matairesinol confer anti-allergic effects in an allergic dermatitis mouse model. J. Ethnopharmacol. 187, 49–56. 10.1016/j.jep.2016.04.015 27085937

[B31] van Dierendonck XAMH Vrieling F. Smeehuijzen L. Deng L. Boogaard J. P. Croes C. A. (2022). Triglyceride breakdown from lipid droplets regulates the inflammatory response in macrophages. Proc. Natl. Acad. Sci. U. S. A. 119 (12), 10.1073/pnas.2114739119 e2114739119 PMC8944848 35302892

[B32] Wang J. Luo L. Zhao X. Xue X. Liao L. Deng Y. (2022). Forsythiae Fructuse extracts alleviates LPS-induced acute lung injury in mice by regulating PPAR-γ/RXR-α in lungs and colons. J. Ethnopharmacol. 293, 115322. 10.1016/j.jep.2022.115322 35483561

[B33] Wang Z. Xia Q. Liu X. Liu W. Huang W. Mei X. (2018). Phytochemistry, pharmacology, quality control and future research of *Forsythia suspensa* (Thunb.) Vahl: a review. J. Ethnopharmacol. 210, 318–339. 10.1016/j.jep.2017.08.040 28887216

[B34] Xia W. Dong C. Yang C. Chen H. (2016). Advances in chemical constituents and pharmacology of. Forsythia Suspensa. Mod. Chin. Med. 18 (12), 1670–1674. 10.13313/j.issn.1673-4890.2016.12.031

[B35] Yang L. Zhou X. Huang W. Fang Q. Hu J. Yu L. (2017). Protective effect of phillyrin on lethal LPS-induced neutrophil inflammation in zebrafish. Cell Physiol. biochem. 43 (5), 2074–2087. 10.1159/000484192 29059681

[B36] Zhang L. Lang F. Feng J. Wang J. (2024). Review of the therapeutic potential of Forsythiae Fructus on the central nervous system: active ingredients and mechanisms of action. J. Ethnopharmacol. 319 (Pt 2), 117275. 10.1016/j.jep.2023.117275 37797873

[B37] Zhang Y. Luo F. Hu H. (2022). Effect of *Forsythia suspensa* extract on the level of ICAM-1 and INF-γ in chronic pelvic inflammatory model rats. Med. J. West China 34 (2), 190–194. 10.3969/j.issn.1672-3511.2022.02.007

[B38] Zhao P. Piao X. Pan L. Zeng Z. Li Q. Xu X. (2016). *Forsythia suspensa* extract attenuates lipopolysaccharide-induced inflammatory liver injury in rats via promoting antioxidant defense mechanisms. Anim. Sci. J. 88 (6), 873–881. 10.1111/asj.12717 27753186

[B39] Zhou M. Huo J. Wang C. Wang W. (2022). UPLC/Q-TOF MS screening and identification of antibacterial compounds in *Forsythia suspensa* (thunb.) Vahl leaves. Front. Pharmacol. 12, 704260. 10.3389/fphar.2021.704260 35153732 PMC8831367

