# Supplementary material for: Efficacy of Forsythia suspensa (Thunb.) Vahl on mouse and rat models of inflammation-related diseases: a meta-analysis
Source: Front Pharmacol. 2024 Mar 4;15:1288584. doi: 10.3389/fphar.2024.1288584 (PMC10946063; doi:10.3389/fphar.2024.1288584)
Supplement: Supplementary file 1 [file DataSheet1.zip › Data Sheet 1/Data Sheet 1.pdf]

连翘中对实验性肝损伤有效成分的研究

刘正光　杨丁铭　（药学专业）

提要　连翘为我国常用的中药之一，动物实验和临床观察，对于急性肝炎都有较好的效果。用动物实验，从连翘的有效部分，分离出齐墩果酸及熊果酸为有效成分。

关键词　连翘　齐果墩酸　熊果酸

连翘〔Forsythia Suspensa (Thunb.) Vahl〕为木犀科连翘属落叶灌木，以果实入药。味苦，性微寒，具有清热解毒，散结消肿，我国药典已收载。动物实验证明：对于用四氯化碳致成大鼠的急性肝损伤的连翘治疗，能使肝脏变性、坏死明显减轻。肝细胞内蓄积的糖原及核糖核酸的含量恢复，血清谷丙转氨酶（SGPT）活力显著下降，说明连翘有抗肝损伤作用[1,2]。临床观察对急性肝炎收到了满意的疗效，特别是在降酶退黄作用上，效果较为突出[3,4]。

据文献报道连翘的成分有：三帖酸类（如熊果酸、齐墩果酸等），木脂素类和甙类及芦丁等[5,6,7,8,9]。

我们在连翘治疗肝炎临床有效的基础上，对连翘的有效成分进行了分离，鉴定现报道如下：

一、样品来源　市售连翘，产于山西夏县，去心粉碎。

二、分离　经粉碎的连翘置圆底烧瓶中，加甲醇，加热回流2小时，连续提取3次，合并提取液，回收甲醇至1/10，加10倍量水，过滤。

滤液在水浴上浓缩至膏状，得连翘A。

沉淀加1％氢氧化钾溶液溶解，过滤、滤液加1：1盐酸呈酸性，过滤，沉淀水洗至无氯离子。于100℃烤干。得连翘B（黄色粉状物）。

三、连翘A和连翘B对大鼠SGPT活力的影响　采用四氯化碳急性肝损伤动物模型的方法[10]结果如表。

表　连翘A和连翘B对大鼠SGPT活力的影响

组别	动物数	$\overline{X} \pm S$
正常组	6	66.9±11.4
对照组	7	340.6±240.6
连翘A组	7	246.3±278.7
连翘B组	6	75.0±13.0

$t = 2.915$　　$P < 0.05$

(C)1994-2024 China Academic Journal Electronic Publishing House. All rights reserved.　http://www.cnki

连翘A无降转氨酶作用，连翘B有明显的降转氨酶作用。

四、连翘B的分离 将连翘B加乙醇溶解、再加入等量的粗硅胶混匀，100℃烤干，研细，放入沙氏提取器中，先用石油醚（60～90℃）提取3～4小时，残渣挥去石油醚，改用乙醚提取15～20小时。滤出提取液的沉淀，回收乙醚至出现沉淀，过滤，合并沉淀，得白色粉状物。

将白色粉状物溶于适量乙醇，煮沸加活性炭脱色，滤液放置，待析出白色沉淀物后，过滤，滤液回收至1/2，放置，析出针状结晶，用乙醇再结晶，得连翘B_1。滤液继续回收至½，放置数日，析出团状针形结晶，用乙醇再结晶，得连翘B_2。

五、连翘B_1和B_2的鉴定

（1）连翘B_1的鉴定

熔点： 282～283℃（温度计未校正）。

红外光谱：$IR_{Max}^{KBr}cm^{-1}$，3527，3400，2949，1716，1454，1390，1378，1358；（A区三个吸收峰）1325，1280，1240；（B区三个吸收峰）与熊果酸红外光谱图一致。

薄层层析：吸附剂：硅胶G（EMK）；展开剂：氯仿—甲醇（8∶2）；显色剂：10%硫酸。结果：紫红色斑点，与样品熊果酸Rf值一致。

根据以上实验连翘B_1为熊果酸。

（2）连翘B_2的鉴定

熔点：306～307℃（温度计未校正），B_2与样品齐墩果酸混融，熔点不下降。

红外光谱：$IR_{max}^{KBr}cm^{-1}$：3400，2920，2860，1680，1460，1380，1040，750；与样品齐墩果酸红外光谱一致。

薄层层析：吸附剂：硅胶G（EMK）；展开剂：氯仿—甲醇（8∶2）；显色剂：10%硫酸。结果：紫红色斑点与样品齐墩果酸Rf值一致。

根据以上实验连翘B_2为齐墩果酸。

连翘B对动物实验有明显的降转氨酶作用，从连翘B中分离出的齐墩果酸在治疗肝炎方面已有不少报道[11,12]，并且已有商品出售。熊果酸也有动物实验能降低血清转氨酶作用及临床治疗肝炎的报道[13,14]。因此可以认为齐墩果酸和熊果酸为连翘治疗肝炎的有效成分。

（山西省医药研究所代做动物试验，卫生部药品生物制品检定所梁文藻同志提供熊果酸样品，山西省药检所代做红外光谱，特此致谢。）

参考文献

1．山西医学院肝病研究组．新医药杂志 1973；(9)：21

2．山西医学院肝病研究组．山西医药 1973；(9)：44

3．山西医学院肝病研究组，太原市传染病医院肝炎病区．医卫通讯 1973；(3)：24

4．周良楣，等．中药连翘制剂对急性肝炎的临床观察，内部资料

5．西部三省，等．药学杂志 1977；97(10) 1134

6．千葉真理子，等．生药学杂志 1979；32(3) 194

7．梁文藻，等．药物分析杂志 1985；5(1)：1

8．梁文藻，等．药物分析杂志 1985；5(2)：79

(C)1994-2024 China Academic Journal Electronic Publishing House. All rights reserved. http://www.cnki

9. 梁文藻，等. 药物分析杂志 1985；5(2):67
10. 马学虑，等. 药学学报 1982；(2):93
11. 湖南医药工业研究所. 中草药通讯 1977；(4):32
12. 续立志，等. 中草药通讯 1979；(8):25
13. 杨模坤，等. 中草药通讯 1981；(11):9
14. 温志坚，等. 中药药理与临床 1987；(1):38

山西医学院学报1988年第19卷第1期

脊柱侧凸后继发性改变的力学分析

谭瑞诚 韩西城 张正之 （第一附属医院骨科）
吴文周 （太原工业大学数力系）

提要 脊柱侧凸后的继发性改变不仅仅是脊柱骨与关节的畸形，更重要的是这种畸形往往会引起某些内脏的功能障碍如心、肺、胃肠和神经、脊髓等功能受到影响，因此本文以力学原理来解释脊柱侧凸，有利于改进对脊柱侧凸的治疗方法，也使人们对脊柱侧凸对人体影响引起足够的重视。

关键词 脊柱侧凸 生物力学

脊柱的某一段偏离身体中线称脊柱侧凸，按病因其分为：①先天性脊柱侧凸；②后天性脊柱侧凸；③原发性（特发性）脊柱侧凸。绝大多数为原发性脊柱侧凸约占80%左右。故本文着重讨论原发性脊柱侧凸后的继发性改变的力学分析，以便对脊柱侧凸的进一步了解。

一、脊柱侧凸对肌肉、韧带、神经根、肋间神经及脊髓的影响

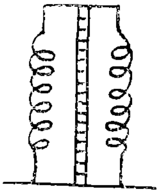

图1 正常脊柱两旁肌肉的张力平衡。脊柱两旁的肌肉相似弹簧，脊柱正常时两旁的拉力相等。否则脊柱侧凸。

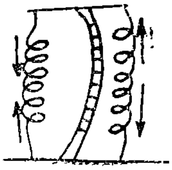

图2 脊柱侧凸后，凸侧椎旁肌肉、韧带张力增大。

脊柱侧凸时造成脊柱两旁不对称，从而破坏了它的平衡，早期凸侧受牵拉的肌肉呈痉挛状态、肌肉若长期被牵拉痉挛而产生肌营养不良性改变及劳损而发生肌肉萎缩。凸

(C)1994-2024 China Academic Journal Electronic Publishing House. All rights reserved. http://www.cnki
