# Supplementary material for: Efficacy of Forsythia suspensa (Thunb.) Vahl on mouse and rat models of inflammation-related diseases: a meta-analysis
Source: Front Pharmacol. 2024 Mar 4;15:1288584. doi: 10.3389/fphar.2024.1288584 (PMC10946063; doi:10.3389/fphar.2024.1288584)
Supplement: Supplementary file 1 [file DataSheet1.zip › Data Sheet 1/This meta-analysis included research articles written in Chinese and we packed all these Chinese publications as the supplementary file/Fan et al 2013.pdf]

连翘对重症急性胰腺炎大鼠肝组织中 NF－κB 和 Foxp3 表达的影响

范晓彬，李文星，陈炳合，熊泽翼，段吉明
（山西医科大学第二临床医学院 普外科，太原 030001）

摘要：目的 探讨核因子 NF－κB 和 Foxp3 在重症急性胰腺炎（SAP）肝损伤中的作用及连翘对其表达活性的影响。**方法** 雄性 Wistar 大鼠 80 只，随机分成假手术组（SO 组）、SAP 组和干预组，其中干预组分连翘高、中、低剂量组和阳性对照组（PDTC）。牛磺胆酸钠溶液在胰胆管远端注射造模，SO 和 SAP 组于术后 3、6、12 h，干预组于术后 12 h 处死大鼠，分别留取标本。测各组血淀粉酶（AMY）、ALT 及 TNFα 水平，鲎试剂法测血浆内毒素水平，流式细胞术测外周血 Treg 百分数，对胰腺及肝脏进行病理学检查及评分，RT－PCR 法检测肝脏组织中 NF－κBmRNA 和 Foxp3mRNA 表达量。组间比较采用单因素方差分析，进一步进行多重比较，采用 LSD 法进行统计学处理，各指标间相关性分析采用直线相关分析。**结果** 与 SO 组比较，SAP 组中各项指标均随时间升高，于 12 h 达高峰。与 SAP12 h 组相比，干预组（大鼠死亡率为 0）肝脏组织中的 NF－κBmRNA 和 Foxp3mRNA 表达明显降低（$P<0.01$），与 Treg 呈正相关（$r=0.738$，$P<0.01$）。随连翘剂量增加，AMY、ALT 及 TNFα 水平均明显降低，肝脏和胰腺组织炎症明显减轻，高剂量组和阳性对照组相比较无明显差异（$P>0.05$）。**结论** NF－κB 的激活参与 SAP 肝损伤的发生，连翘能显著降低 NF－κB 的活性及肝脏组织中 NF－κBmRNA 和 Foxp3mRNA 的表达，减轻 SAP 肝损伤的严重程度。

关键词： 胰腺炎，急性坏死性；NF－κB；转录因子；连翘
中图分类号： R576；R575　　**文献标志码：** A　　**文章编号：** 1001－5256（2013）07－0503－05

Effects of Forsythia suspensa on expression of NF－κB and Foxp3 during liver injury in rats with severe acute pancreatitis

FAN Xiaobin，LI Wenxing，CHEN Binghe，et al.（Department of General Surgery，The Second Hospital of Shanxi Medical University，Taiyuan 030001，China）

Abstract: Objective To investigate the roles of nuclear factor－kappa B（NF－κB）and forkhead box P3（Foxp3）during liver injury in rats with severe acute pancreatitis（SAP）and the effects of Forsythia suspensa on their expression. **Methods** Eighty male Wistar rats were randomly allocated into sham operation（SO）group，SAP group，and intervention group. The intervention group was further divided into high－dose，middle－dose，and low－dose Forsythia suspensa subgroups and positive control（PDTC）subgroup. A rat model was induced by injecting sodium taurocholate into the bile－pancreatic duct. The rats in SO and SAP groups were sacrificed at 3，6，and 12 h after operation，and those in intervention group were sacrificed at 12 h after operation. The serum levels of amylase（Amy），alanine aminotransferase（ALT），and tumor necrosis factor（TNF）α were measured. The endotoxin content in plasma was determined using a limulus reagent. The percentage of Treg cells among peripheral blood lymphocytes was measured by flow cytometry. The pathological examination and scoring of the liver and pancreas were performed. The mRNA expression levels of NF－κB and Foxp3 in liver tissue were measured by RT－PCR. One－way analysis of variance was used for comparison among groups；multiple comparison was performed by LSD test；the indices were subjected to linear correlation analysis. **Results** Compared with those in the SO group，all the indices in the SAP group increased over time and reached the peak levels at 12 h after operation. Compared with the rats in SAP group that were sacrificed at 12 h after operation，the intervention group（mortality = 0）had significantly decreased mRNA expression of NF－κB and Foxp3 in liver tissue（$P<0.01$）. The NF－κB mRNA level was positively correlated with the percentage of Treg cells（$r=0.738$，$P<0.01$）. As the dose of Forsythia suspensa rose，Amy，ALT，and TNFα levels decreased significantly，and the inflammation of liver and pancreas was significantly alleviated. There were no significant differences between the high－dose Forsythia suspensa subgroup and PDTC subgroup（$P>0.05$）. **Conclusion** Activation of NF－κB contributes to the liver injury in rats with SAP. Forsythia suspensa can significantly reduce the activation of NF－κB and the mRNA expression of NF－κB and Foxp3 in liver tissue，thus alleviating the liver injury in rats with SAP.

Key words: pancreatitis，acute necrotizing；NF－kappa B；transcription factors；forsythia suspensa

doi：10.3969/j.issn.1001－5256.2013.07.007
收稿日期： 2013－01－23；**修回日期：** 2013－02－25。
基金项目： 山西省回国留学人员科研资助项目（2012－090）
作者简介： 范晓彬（1982－），男，主要从事肝胆胰疾病研究。
通信作者： 李文星，电子信箱：doctorli5393@163.com。

(C)1994-2022 China Academic Journal Electronic Publishing House. All rights reserved. http://www.cnki.net

重症急性胰腺炎(severe acute pancreatitis,SAP)是病死率非常高的临床急腹症,死亡主要的原因是细胞因子的激活、免疫功能的紊乱以及炎症介质的释放。因血流动力学和解剖的关系,肝脏成为SAP最早累及的胰外器官之一,其并发肝损伤的机率可高达88.9%[1],其机制主要是失控性炎症反应及机体免疫功能紊乱。而核因子(NF)-κB在其发展中起着"信使"作用。作为重要的转录因子,NF-κB参与免疫反应和淋巴细胞分化,其活化可能调控着SAP的启动。NF-κB信号通路对Foxp3表达的调控起着非常关键的作用,当NF-κB被激活或信号强度增强时可直接促进Foxp3的表达和Treg细胞的增加,从而分泌大量的抑制性细胞因子来调节免疫功能的紊乱。SAP肝损伤重要的发病机制之一为肠源性内毒素血症,祖国医学认为内毒素血症在病机上为邪实正虚,所以治疗应采用祛邪与扶正的原则[2]。连翘具有抗内毒素、抗炎,又有其显著的增强机体免疫和解毒系统对内毒素予以解毒的功效[3]。且目前尚未见在SAP肝损伤中应用的报告。本实验拟探讨NF-κB和Foxp3在SAP肝损伤中的作用机制及连翘对其表达活性和SAP肝损伤保护的影响。

1　材料与方法

1.1　实验动物　健康雄性Wistar大鼠80只,体重(250±20)g,山西医科大学实验动物中心提供。

1.2　主要试剂及仪器　牛磺胆酸钠(NaTc)购自美国Sigma公司,Anti Rat CD4 FITC及Anti-Rat CD25 PE均由美国eBioscience公司提供,兔抗大鼠Foxp3mRNA单克隆抗体和兔抗大鼠NF-κB p65单克隆抗体(武汉博士德生物有限公司提供),鲎试剂盒购自厦门鲎试剂公司,流式细胞仪FACScalibur(美国BD公司),RT-PCR试剂盒(Gibco公司),TRIzol(Ivitrigon公司),PCR引物、DNA Marker(上海赛百盛公司),PDTC购自美国Sigma公司,实验用连翘为木犀科植物贯叶连翘的干燥果实,购自山西医科大学第二医院中药房(经省中医学院郑学军教授鉴定,符合现行中国药典药用标准)。

1.3　实验动物分组及模型制备　80只大鼠随机分为SO组(24只)、SAP组(24只),其中每组再分3、6、12 h三个亚组(n=8),和干预组(32只)其中包括:连翘高、中、低剂量组(AFS1、AFS2、AFS3)和阳性对照组(PDTC)每组各8只。连翘干预组在造摸前7 d开始给药,给予连翘水煎液高(5 g/kg)、中(2.5 g/kg)、低(1.25 g/kg),每天1次,灌胃。阳性对照组于造摸前1 h给药,100 mg/kg,腹腔注射。实验大鼠术前禁食过夜,用1%戊巴比妥钠(0.4 ml/100g)腹腔内注射麻醉,消毒,铺无菌巾。取剑突下切口入腹腔,提起胃、十二指肠,显露胰脏,确认胆胰管,以0.1 ml/min的速度经胆胰管远端缓慢逆行推注5%牛磺胆酸钠溶液(0.1 ml/100g),推注完毕压迫穿刺点5 min,去除小血管夹及丝线,查无胆漏,关腹。SO组仅开腹翻动胰腺两次,余同SAP组。

1.4　连翘水煎液的制备方法　称取连翘药材200 g,加水煎2次,2 h/次,滤过合并煎液,浓缩,定容至80 ml,过滤后质量浓度成为25 g/ml[3],放4 ℃冰箱备用。

1.5　实验标本留取　术后各时间点处死大鼠,腹主动脉取血,抗凝处理后取1 ml全血立即送检,余离心、分离后于-20℃冻存,取部分肝和胰腺组织液氮冷冻后于-70℃冰箱冻存,剩余部分肝和胰腺组织在10%甲醛溶液中固定,常规石蜡包埋。干预组于术后12 h处死大鼠,处理同上。

1.6　观察指标及方法

1.6.1　血浆和血清检测　血浆血淀粉酶(AMY)及ALT用全自动生化分析仪检测。血浆内毒素(endotoxin,ET)检测用鲎试剂法,按照试剂盒说明检测。血清肿瘤坏死因子(TNF)α按试剂盒说明进行放射免疫分析法检测。

1.6.2　胰腺和肝脏组织病理学检测及评分　胰腺组织:制作胰腺组织HE染色切片,采用盲法光镜下阅片,随机选择10个高倍视野。肝脏组织:方法同上。

1.6.3　外周血Treg检测　流式细胞术检测外周血Treg亚群百分数。取肝素抗凝血80 μl加入试管,分别加入Anti-Rat CD4 FITC 2 μl及Anti-Rat CD25 PE 2.5 μl,室温避光孵育15 min后加入1 ml红细胞裂解液,混匀室温下孵育10 min,2 ml PBS洗涤2次,1000 r/min离心5 min,弃上清悬浮细胞后上机检测。Cell Quest软件分析数据,$CD4^+$ FITC/$CD25^+$ PE均标记上的细胞为阳性细胞,阳性细胞与淋巴细胞之比为Treg细胞百分数。

1.6.4　RT-PCR法检测肝脏组织NF-κB和Foxp3mRNA的表达量　TRIzol试剂提取肝脏组织总RNA,并对其浓度和纯度以及完整性进行检测;总RNA 1 μg经逆转录合成cDNA第1链;NF-κB PCR扩增引物:上游5′-AACACTGCCGAGCTCAAGAT-3′,下游5′-CATCGGCTTGAGAAAAGGAG-3′(163bp);Foxp3 PCR扩增引物:上游5′-CGGGAGAGTTTCTCAAGCAC-3′,

(C)1994-2022 China Academic Journal Electronic Publishing House. All rights reserved.　http://www.cnki.net

下游 5′－GGAGCTCTTGTCCACTGAGG－3′(206bp)；Beta－actin PCR 扩增引物：上游 5′－GTCAGGTCATCACTATCGGCAAT－3′，下游 5′－AGAGGTCTTTACGGATGTCAACGT－3′(147bp)，PCR 扩增条件：94 ℃预变性 10 min，94 ℃变性 15 s，60 ℃退火 60 s，72 ℃延伸 1 min，72 ℃后延伸 5 min，扩增 45 个循环结束。荧光定量 PCR 读取 C_T(cycle threshold) 值，首先计算各样本测定基因 P65 和 Foxp3 的 C_T 与内对照基因 Beta－actin C_T 的差值，即 $\Delta C_T = C_{T(P65和Foxp3)} - C_{T(beta-actin)}$，再用各实验组 P65 和 Foxp3 样本的 ΔCT 减去正常对照组 P65 和 Foxp3 样本的 ΔC_T，得到 $\Delta\Delta C_T$，利用 $2^{-\Delta\Delta C_T}$ 进行计算，表示实验组测定基因 P65 和 Foxp3 的表达相对与正常对照组样本 P65 和 Foxp3 表达的变化倍数[8]。

1.7 统计学处理 应用 SPSS16.0 统计软件分析，定量数据以均数±标准差($\bar{x} \pm s$) 表示，组间比较采用单因素方差分析，进一步进行多重比较，采用 LSD 法进行统计学处理，各指标间相关性分析用直线相关分析，以 $P < 0.05$ 为差异有统计学意义。

2 结果

2.1 血浆 AMY、ALT、TNFα、ET 水平变化及血浆 Treg 表达变化 损伤后，SAP 组 AMY、ALT、TNFα、ET 及 Treg 变化较同时点 SO 组明显增加，并于损伤后 12 h 达高峰，连翘干预组和阳性对照组血浆中 AMY、ALT、TNFα、ET 及 Treg 变化较 SAP 12 h 组降低(表 1)。

2.2 大鼠肝脏、胰腺组织形态学变化 光镜下观察 SO 组为正常肝脏结构，SAP 组 3 h 可见肝细胞轻度肿胀，少量肝细胞气球样变和淋巴细胞浸润；6 h 肝细胞可见点状坏死，肝窦可见增宽，明显气球样变，肝细胞中度肿胀，胞浆疏松，汇管区可见淋巴细胞浸润；12 h 肝细胞气球样变，局灶性片状坏死，中性粒细胞和淋巴细胞大量浸润。镜下见 SO 组胰腺结构完整，轮廓清晰。SAP 组随时间延长病理改变越来越重，腺泡破裂，结构模糊，淋巴细胞大量浸润，伴胰腺组织灶性及片性出血。连翘和 PDTC 处理组组织变性坏死明显减轻(表 2)。

2.3 肝脏组织 NF－κBmRNA 和 Foxp3mRNA SAP 组肝脏组织中 NF－κBmRNA 和 Foxp3mRNA 12 h 的表达量比 3、6 h 明显升高，与 SO 组相应时间点相比，差异有统计学意义($P < 0.01$)。连翘干预组(AFS1、AFS2、AFS3)和 PDTC 处理组中 NF－κBmRNA 和 Foxp3mRNA 表达量较SAP12h组明显下降，且AFS1、AFS2、AFS3三者有量效关系，各组与 SAP 组 12 h 相比，差异有统计学意义($P < 0.01$)，以 AFS1 和 PDTC 处理组下降最明显(但均未降至正常水平)，AFS1 和 PDTC 组两者之间比较差异无统计学意义($P > 0.05$)(表 3)。

表 2 各组大鼠胰腺和肝脏病理学评分情况($\bar{x} \pm s$)

组别	时间	胰腺病理评分	肝脏病理评分
SO	3 h	0.00±0.00 2)	0.00±0.00 2)
	6 h	0.45±0.11 2)	0.11±0.02 2)
	12 h	0.55±0.12 2)	0.21±0.02 2)
SAP	3 h	5.54±0.81 1)2)	1.35±0.06 1)2)
	6 h	7.46±1.04 1)2)	1.97±0.15 1)2)
	12 h	11.29±0.92 1)	2.29±0.13 1)
AFS1	12 h	6.27±0.70 2)	1.53±0.09 2)
AFS2	12 h	7.93±0.87 2)	1.72±0.07 2)
AFS3	12 h	10.28±0.78 2)	2.19±0.11 2)
阳性对照	12 h	5.84±0.91 2)	1.49±0.09 2)

注：与同一时点 SO 组相比，1) $P < 0.01$，与 SAP12 h 组相比，2) $P < 0.05$

表 1 外周血 AMY、ALT 及 Treg 的水平动态变化($\bar{x} \pm s$)

组别	时间	AMY(U/L)	ALT(U/L)	ET(Eu/ml)	Treg(%)	TNFα(ng/ml)
SO	3 h	1201.45±106.89 2)	82.04±3.84 2)	0.40±0.02 2)	1.65±0.07 2)	0.35±0.07 2)
	6 h	1274.19±80.33 2)	91.15±3.64 2)	0.49±0.05 2)	1.67±0.07 2)	0.36±0.07 2)
	12 h	1327.44±50.33 2)	99.05±3.48 2)	0.67±0.05 2)	1.72±0.06 2)	0.41±0.10 2)
SAP	3 h	4913.36±375.02 1)2)	193.55±9.53 1)2)	0.78±0.05 1)2)	2.07±0.10 1)2)	1.57±0.09 1)2)
	6 h	6161.40±413.22 2)	389.87±10.35 1)2)	0.91±0.03 1)2)	2.20±0.06 1)2)	1.95±0.12 1)2)
	12 h	8220.74±364.50 1)	859.47±18.13 1)	1.01±0.03 1)	2.63±0.11 1)	2.03±0.12 1)
AFS1	12 h	5322.26±307.10 2)	239.43±10.01 2)	0.81±0.03 2)	1.69±0.08 2)	1.70±0.08 2)
AFS2	12 h	6217.19±348.17 2)	293.06±11.02 2)	0.89±0.04 2)	1.83±0.06 2)	1.82±0.09 2)
AFS3	12 h	8013.68±397.29	779.32±16.39 2)	0.96±0.05 2)	1.97±0.08 2)	1.94±0.12 2)
阳性对照	12h	5141.78±316.15 2)	218.25±9.09 2)	0.79±0.03 2)	1.67±0.05 2)	1.66±0.13 2)

注：与同一时点 SO 组相比，1) $P < 0.01$；与 SAP 12 h 组相比，2) $P < 0.05$

(C)1994-2022 China Academic Journal Electronic Publishing House. All rights reserved. http://www.cnki.net

2.4 NF－κB与Treg相关性分析 相关性分析表明,肝脏组织NF－κBmRNA与血浆Treg表达呈正相关,相关系数为0.738($P<0.01$)。

表3 肝脏组织中NF－κBmRNA和Foxp3mRNA水平表达动态变化($\bar{x}\pm s$)

组别	时间	NF－κBmRNA	Foxp3mRNA
SO	3 h	0.13±0.04 2)	0.04±0.02 2)
	6 h	0.14±0.02 2)	0.05±0.01 2)
	12 h	0.15±0.05 2)	0.06±0.03 2)
SAP	3 h	0.36±0.08 1)2)	0.68±0.09 1)2)
	6 h	1.34±0.08 1)2)	2.75±0.46 1)2)
	12 h	1.85±0.11 1)	6.68±0.76 1)
AFS1	12 h	0.65±0.16 2)	1.36±0.17 2)
AFS2	12 h	0.96±0.09 2)	1.89±0.26 2)
AFS3	12 h	1.55±0.08 2)	4.49±1.12 2)
阳性对照	12 h	0.55±0.09 2)	1.21±0.15 2)

注:与同一时点SO组相比,1) $P<0.01$;与SAP12 h组相比,2) $P<0.05$

3 讨论

SAP时失控性炎症反应引起大量细胞因子的释放及机体免疫功能严重紊乱,导致机体的多脏器功能障碍(multiple organ dysfunction syndrome, MODS)和多器官功能衰竭(multiple organ failure, MOF),从而使患者死亡[7-8]。在SAP的众多并发症中其合并肝损伤的机率可高达88.9%[1],肝损伤后反过来又可加重SAP的病情,影响着SAP的治疗及预后。在本实验中:SAP各组大鼠的病理可见肝细胞不同程度的变性和坏死以及ALT的升高,提示SAP大鼠存在肝损伤。

SAP肝损伤的机制很多,目前公认大量细胞因子的激活和炎症介质的释放在其中发挥重要作用。NF－κB广泛存在于肝细胞内,是介导炎症反应的关键分子,并在天然免疫和获得性免疫中起着重要作用。SAP时内毒素、炎症介质、胰酶等激活肝脏中的NF－κB后,诱导多种促炎细胞因子(如TNFα、IL－1)的表达[9],产生级联放大效应并形成恶性循环,引起免疫调节的失衡。有报道称,注射脂多糖(LPS)后的大鼠,肝组织中NF－κB阳性细胞表达率在短时间内就可见明显升高[10]。Treg可分泌大量抗炎细胞因子(如IL－10、TGFβ),调节Th1/Th2水平来影响SAP的发生发展,其细胞活性水平可通过Treg的特征性标志Foxp3来反映[11],且与Treg的免疫调节功能密切相关[12]。天然Treg细胞在体内依靠IL－2和TGFβ来维持Foxp3的表达,当NF－κB被激活或信号强度增强时,作为家族成员的c－Rel－P50和c－Rel－P65可以与Foxp3基因位点上非编码序列中的CNS1启动子及CNS3增强子区域CPG岛结合[13],直接促进Foxp3的表达和Treg细胞的增加。在本实验中,SAP组肝脏中NF－κBmRNA和Foxp3mRNA表达随时间点延长而增加,与病理的严重程度呈正相关,提示SAP后发生严重的免疫紊乱和炎症发应,推测NF－κB在SAP肝损伤免疫紊乱中可能发挥着重要作用。同时外周血中Treg变化与两者基本一致,说明SAP后引起的NF－κB激活,导致Foxp3表达和Treg细胞增加,而Treg分泌大量抑制性细胞因子来抑制NF－κB的活化,调节机体免疫平衡,来影响SAP的发展和转归。

SAP时,肝脏清除肠道来的大量内毒素的能力明显减弱,致使血液中的高内毒素对机体的造成“二次打击”[14-15],进一步引起胰腺和肝脏等多脏器损伤,本实验显示,血液中内毒素的水平和Treg增加,既然Treg可以分泌大量的抗炎细胞因子,因此可以认为SAP后不会发生过度炎症反应,甚至可抑制反应,但SAP组肝脏组织中NF－κBmRNA表达增加,同时也与SAP后极易发展成全身炎症反应综合征、MODS、MOF的事实相悖,所以在本实验中作者认为,Treg数量的增多能减轻炎症,但又可以下调对病原体的控制能力,引起内毒素移位和细菌感染机率增加,导致严重的免疫功能紊乱和大量的促炎细胞因子(如TNFα)释放,对脏器造成损伤。这也证实了SAP大鼠中经典的LPS→NF－κB→Treg→细胞因子途径的存在。中药连翘有抗炎、抗内毒素、清热解毒、抗肝损害、免疫调节等作用,同时能调控NF－κB途径的活化。有报道称,连翘是直接对内毒素进行摧毁而非短时间的抑制[16],也有学者报道连翘败毒片能提高机体细胞免疫和体液免疫[15],傅强等[17]报道连翘31.25 g/L浓度以上时即可对内毒素显著拮抗,也可抑制内毒素诱发的炎性细胞因子的大量表达;韩双红等[18]的研究发现连翘败毒片可显著促进小鼠血清抗体生成和增强小鼠的迟发型超敏反应,具有提高小鼠机体体液免疫和细胞免疫功能的作用。在本实验的干预组中,可见AFS1、AFS2、AFS3组可显著下调外周血中ET、ALT及Treg,同时也下调了肝脏组织中NF－κBmRNA和Foxp3mRNA的表达,使炎症介质和细胞因子的水平

(C)1994-2022 China Academic Journal Electronic Publishing House. All rights reserved. http://www.cnki.net

降低，减轻了 SAP 的炎症反应和病理改变，其中以 AFS1 的作用最显著，且 AFS1 与 NF－κB 特异性抑制剂的 PDTC 组产生的效应基本相同。说明连翘有明显的抗 NF－κB 和调节 Treg 的作用，可逆转 SAP 对机体免疫系统的抑制作用。同时，本实验 AFS1、AFS2、AFS3 三组指标的结果差异较大，这就说明连翘的抗内毒素和免疫调节作用存在量效关系。这为连翘在防治 SAP 免疫失衡的应用提供了实验依据。

总之，SAP 肝损伤原因可能是 NF－κB 被激活后大量促炎细胞因子的释放和外周血 Treg 水平显著升高共同作用的结果，同时 Treg 下调了对病原体的控制能力，引起了严重的肠源性内毒素血症，使大鼠的免疫系统极度紊乱，而连翘在拮抗内毒素的同时，还可以下调 NF－κBmRNA 和 Foxp3mRNA，体现了对抗性和保护性治疗的结合。

参考文献：

[1] HALONEN K，PETTILA V，LEPPANIEMI AK，et al. Multiple organ dysfunction associated with severe acute pancreatitis [J]. Crit Care Med，2002，30(6)：1274－1279.

[2] SHI JQ，FU YJ，XIE Y，et al. The effect of forsythia suspense on anti－endotoxin and LPS－TLR4 signal－transducing pathway [D]. Nanchang University，2007，51：87－90.（in Chinese）
石俊强，傅颖珺，谢勇，等. 连翘抗内毒素作用及其对 LPS－TLR4 信号通路的影响 [D]. 南昌大学，2007，51：87－90.

[3] FU YJ，YUAN JL，CHEN J，et al. Forsythiae to the effect of peripheral blood and spleen of Foxp3 in severely burned rats [J]. Chin J Cell Mol Immunol，2009，25(10)：935－938.（in Chinese）
傅颖珺，袁娟丽，陈江，等. 连翘对严重烧伤大鼠外周血 Treg 及脾脏 Foxp3 的影响 [J]. 细胞与分子免疫学杂志，2009，25(10)：935－938.

[4] SCHMIDT J，LEWANDROWSKI K，WARSHAW AL，et al. Morphometric characteristics and homogeneity of a new model of acute pancreatitis in the rat [J]. Int Pancreatol，1992，12(1)：41－51.

[5] CAMARGO CA，MADDEN JF，GAO W，et al. Intefleukln－6 protects liver against WRXIH is chemia/reperfusion injury and promotes hepatocyte proliferation in the rodent [J]. Hepatology，1997，26(6)：1513－1520.

[6] PFAFFL MW. A new mathematical model for relative quantification in real－time RT－PCR [J]. Nucl Acids Res，2001，29(9)：2002－2007.

[7] CHEN MF，WANG FM. Advances in severe acute pancreatitis treatments [J]. J Clin Hepatol，2012，28(8)：571－572.（in Chinese）
陈梅福，王方明. 重症急性胰腺炎治疗的进展 [J]. 临床肝胆病杂志，2012，28(8)：571－572.

[8] SUN HW，WANG MM，ZHANG T，et al. Eeffect of LXA_4 on the expression of NF－κB and ICAM－1 in the process of SIRS induced by ANP [J]. J Med Res，2012，41(3)：24－27.（in Chinese）
孙洪伟，汪茂名，张涛，等. LXA_4 对 ANP 诱发 SIRS 过程中 NF－κB、ICAM－1 表达的影响 [J]. 医学研究杂志，2012，41(3)：24－27.

[9] FUJIOKA S，NIU J，SCHMIDT C，et al. NF－kappaB and AP－1 connection：Mechanism of NF－kappaB－dependent regulation of AP－1 [J]. Mol Cell Biol，2004，24(17)：7806－7819.

[10] ZHANG WS，LENG QX，WANG JP. The effect of transcription factor NF－κB in the endotoxin shock [J]. Prog Anat Sci，2005，11(2)：161－163，166.（in Chinese）
张维山，冷启新，王金平. 转录因子 NF－κB 在内毒素休克中的作用 [J]. 解剖科学进展，2005，11(2)：161－163，166.

[11] RAO DN，NAQVI RA. FoxP3：A key player in T regulatory biology [J]. Ind J Clin Biochem，2011，26(1)：1－2.

[12] HAIQI H，YONG Z，YI L. Transcriptional regulation of Foxp3 in regulatory T cells [J]. Immunobiology，2011，216(6)：678－685.

[13] LONG M，PARK SG，STRICKLAND I，et al. Nuclear factor－κB modulates regulatory T cell development by directly regulating expression of foxp3 transcription factor [J]. Immunity，2009，31(6)：921－931.

[14] HAIQI H，YONG Z，YI L. Transcriptional regulation of Foxp3 in regulatory T cells [J]. Immunobiology，2011，216(6)：678－685.

[15] HAN DW. The role of enterogenous endotoxin concentration on the pathogenesis of pancreatitis [J]. World Chin J Digest，2006，32(29)：28－31.（in Chinese）
韩德五. 肠源性内毒素血症在胰腺炎发病中的作用 [J]. 世界华人消化杂志，2006，32(29)：28－31.

[16] GAO SJ，DAI XZ，YAO HM. The Comparison test of several medicine that can clearing away heat and toxic materials in anti－endotoxin effect [J]. Tianjin J Tradit Chin Med，1992，9(3)：42－44.（in Chinese）
高淑娟，戴锡珍，要华民. 几种清热解毒中药抗内毒素作用的比较实验 [J]. 天津中医药，1992，9(3)：42－44.

[17] FU Q，CUI HL，CUI NJ. Experimental study on inhibitory effect of forsythia extract on inflammatory response induced by endotoxin [J]. Tianjin Med J，2003，31(3)：161－163.（in Chinese）
傅强，崔华雷，崔乃杰. 连翘提取物抑制内毒素诱导的炎症反应的实验研究 [J]. 天津医药，2003(3)：161－163.

[18] HAN SH，WANG YF，ZHANG JX. Research of anti－endotoxin and immune regulation of forsythiae disease and piece [J]. Tianjin J Tradit Chin Med，2004，21(5)：417－419.（in Chinese）
韩双红，王玉芬，张居馨. 连翘败毒片的抗内毒素及免疫调节作用的研究 [J]. 天津中医药，2004，21(5)：417－419.

（本文编辑：王　莹）

(C)1994-2022 China Academic Journal Electronic Publishing House. All rights reserved. http://www.cnki.net
