# Supplementary material for: Efficacy of Forsythia suspensa (Thunb.) Vahl on mouse and rat models of inflammation-related diseases: a meta-analysis
Source: Front Pharmacol. 2024 Mar 4;15:1288584. doi: 10.3389/fphar.2024.1288584 (PMC10946063; doi:10.3389/fphar.2024.1288584)
Supplement: Supplementary file 1 [file DataSheet1.zip › Data Sheet 1/This meta-analysis included research articles written in Chinese and we packed all these Chinese publications as the supplementary file/Feng et al 2018.pdf]

连翘的研究进展

冯治朋[1] 高秀强[1*] 韩颜超[1] 王芳芳[1] 周盛茂[1] 姜永新[1] 王保琼[1] 田清存[1] 崔旭盛[1,2]
([1]石家庄以岭药业股份有限公司,河北石家庄 050035;[2]中国农业大学农学院)

摘要 全面系统地查阅有关连翘的文献资料,综述连翘近年来在化学成分、药理作用、临床应用、栽培现状、质量影响因素及开发利用方面的研究进展,提出连翘栽培的重点研究方向,为连翘资源的综合开发利用提供参考依据。

关键词 连翘;药理作用;栽培现状;质量影响因素
中图分类号 S567 文献标识码 A 文章编号 1007-5739(2018)12-0060-03

Research Progress on *Forsythia Suspensa*(Thunb.)Vahl.

FENG Zhi-peng[1] GAO Xiu-qiang[1*] HAN Yan-chao[1] WANG Fang-fang[1] ZHOU Sheng-mao[1] JIANG Yong-xin[1] WANG Bao-qiong[1] TIAN Qing-cun[1] CUI Xu-sheng[1,2]
([1]Shijiazhuang Yiling Pharmaceutical Co.,Ltd,Shijiazhuang Hebei 050035;[2]College of Agricultural,China Agricultural University)

Abstract The literatures about *Forsythia suspensa* were systematically and systematically inspected,and the research progress of *Forsythia* in chemical composition,pharmacological action,clinical application,cultivation status,quality influencing factors and exploitation and utilization were reviewed in recent years.The key research directions of *Forsythia* cultivated were put forward,in order to provide reference for the comprehensive development and utilization of *Forsythia* resources.

Key words *Forsythia Suspensa*(Thunb.)Vahl.;pharmacological effect;cultivation status;quality influencing factor

连翘是我国的传统中药,始载于《神农本草经》。传统中医认为,连翘有疏散风热、清热解毒、消肿散结的功效,可以治疗风热感冒、温病初起、热淋涩痛、痈疽、瘰疬等证[1];现代药理研究表明,连翘有解热、抗炎、抗病毒、抗菌、抗肿瘤、降压等作用,可治疗临床发热、流感、肿瘤、高血压等症。传统中医药知识和现代药理研究相结合,使连翘具有更好的临床应用前景。

1 种类及分布

木犀科连翘属连翘[*Forsythia suspensa*(Thunb.)Vahl]被《中国药典》(2015版)列为中药"连翘"的唯一基原。据本草考证,宋代以前中药"连翘"多采用金丝桃科湖南连翘(*Hyperium ascyron* L.)为基原,宋代之后中药"连翘"多采用木犀科连翘[*F. suspensa*(Thunb.)Vahl]为基原,并一直沿用至今[2],其资源主要分布于我国山西、陕西、河北、甘肃、宁夏、山东、江苏、河南、江西、湖北、四川及云南等省(区),另外在朝鲜、日本和欧洲也有少量生长。

2 化学成分、药理作用及临床应用

2.1 化学成分

连翘中含多种化学成分,自首次报道从连翘果实中得到酸类化合物以来,已有200余种化学成分被提取分离出来,主要含脂类、苯乙烯类及其衍生物、黄酮类、木脂素类和醇、酯、醚、醛、酮等类化合物。其中连翘苷、连翘脂苷、齐墩果酸等是其作用的主要有效成分[3],其中连翘苷在中国药典中被当作连翘的质量控制指标。

罗彬等[4]利用正相硅胶柱色谱、葡聚糖凝胶Sephadex LH-20、高效液相等手段对连翘提取物进行分离纯化,并通过^{1}H-NMR、^{13}C-NMR、MS等波谱技术鉴定结构,最终分离并鉴定了12个化合物,其中罗汉松脂素、松脂素单甲醚、连翘酯苷G、异槲皮素、紫云英苷为首次从该种植物中分离得到。阎新佳等[5]利用大孔吸附树脂、Sephadex LH-20、反相HPLC等多种色谱对连翘提取物进行分离纯化,最终得到13个单体化合物,其中4-羟基-4-异丙基-1-环己烯甲酸、壬二酸、2-羟基-丁二酸-4-内酯等9个单体化合物为首次从该种植物中分离得到。随着研究的深入,越来越多的化合物将从连翘中分离得到。

2.2 药理作用

2.2.1 解热作用。连翘的现代药理研究的解热作用与其传统中医认为的疏散风热、清热解毒的功效相一致。苏红昌等[6]认为,连翘酯苷A(forsythiaside A,FA)能降低由酵母菌感染引发小鼠体温升高。党珏等[7]研究表明,连翘提取物和连翘挥发油均具有下调酵母致热大鼠下丘脑中cAMP的作用,从而达到解热效果。此外,连翘提取物还具有下调下丘脑中PGE2的作用。

2.2.2 抗炎作用。连翘的抗炎作用与其消肿散结的功效相一致。连翘作为"疮家圣药",更加说明其具有显著的抗炎作用。Kang H S等[8]研究发现,连翘的甲醇提取物中含有牛蒡子苷元,它可抑制动物毛细血管通透性增加和增加动物炎症组织白细胞含量,从而达到止痛和抗炎的作用。全云云等[9]采用酶联免疫法(ELISA)测定由二甲苯致导致耳肿胀的小鼠血清中肿瘤坏死因子-α(TNF-α)和白介素-6(IL-6)的含量,结果表明连翘脂素抗炎效果最好,连翘酯苷A和连翘酯苷B次之,而连翘苷基本无明显抗炎效果,并且认为连翘抗炎的机制有可能是减少TNF-α和IL-6这2种炎症因子的生成。

2.2.3 抗菌、抗病毒。连翘的抗菌、抗病毒作用与其清热解毒功效的药理作用基本一致。连翘有较广的抗菌谱,对细菌和真菌都有一定的抑制作用。刘玉婕等[10]研究表明,连翘和金银花配伍使用耐甲氧西林金黄色葡萄球菌(MDR-MRSA)的活性最高,连翘次之,金银花最低。连翘提取物中

基金项目 国家公益性行业科研专项(201507002);国家中药标准化项目(ZYBZH-C-HEB-12);国家重点研发计划(2017YFC1701700);石家庄市科技研发计划(161200343A)。
作者简介 冯治朋(1988-),男,山东菏泽人,硕士,从事药材基地管理工作。
*通信作者
收稿日期 2018-03-15

(C)1994-2019 China Academic Journal Electronic Publishing House. All rights reserved. http://www.cnki.net

连翘苷可以抑制甲型流感病毒核蛋白(NP)基因的转染后表达,从而达到抗“甲流”的作用,其 3 种不同剂型中,以连翘苷抑制 NP 基因表达量最强,连翘醇提液次之,连翘水提液最弱[11]。

2.2.4 抗氧化作用。科学研究表明,人体衰老的原因与人体内自由基太多,引起氧化太过,消耗太多能量,破坏正常细胞有关。防治衰老与如何防治自由基氧化息息相关。连翘花醇提物可以通过清除活性氧自由基和提高抗氧化酶活力保护线粒体,连翘叶中多酚类物质可以清除亚硝酸盐和阻断亚硝胺的形成[12-13]。李寒冰等[14]采用清除有机自由基 DPPH 法评价连翘 4 种不同规格商品的抗氧化性,发现青翘心抗氧化作用最强,且连翘的抗氧化性与连翘酯苷 A 和槲皮素的含量有关。

2.2.5 对心脑血管系统的作用。Wang 等[15]研究了连翘酯苷(forsythiaside,FTS)对快速老化模型(SAMP8)小鼠的影响,结果表明,FTS 对 SAMP8 小鼠认知障碍具有一定的改善作用,并对其神经具有一定的保护作用,可用于健忘症的治疗。Kim 等[16]研究表明,连翘的主要成分连翘酯苷(FTS)对短暂性脑缺血沙鼠的神经有保护作用,其机制可能与 FTS 诱导活化的神经胶质细胞的减少及 IL-1β、TNF-α 水平的提高有关。Iizuka 等[17]通过大鼠主动脉环离体实验研究了连翘酯苷(FTS)的血管舒张作用,结果表明,FTS 只能抑制由 NE 引起的血管收缩,并推测其作用机制可能是减少 NE 引起的 Ca^{2+}内流有关。

2.2.6 抗肿瘤。肿瘤是一种由环境因素与遗传因素相互作用的复杂性疾病,同时也被称为“21 世纪人类的主要杀手”。近年来,随着研究的发展,采用中药抗肿瘤取得了长足的发展。毛威[18]对连翘抗肿瘤活性的研究发现,连翘中的连翘脂素和表松脂素对人胃癌细胞株 SGC7901 生长有一定的抑制作用,并认为连翘抗肿瘤活性与环氧木质素类物质有关。孙士萍等[19]采用 MTT 法检测连翘根醇提物对食管癌 TE-1、TE-13、Yes-2 和 Eca-109 细胞增殖的抑制作用,结果发现,治疗组裸鼠肿瘤细胞凋亡数量明显高于对照组,Bcl-2 蛋白的表达水平低于对照组,Bax 蛋白表达水平高于对照组。连翘根醇提物通过诱导食管癌细胞凋亡,达到对食管癌细胞的生长抑制作用。

2.2.7 保肝、利胆作用。肝胆疾病是一种常见的多发慢性疾病,严重危害人体健康。冯 芹等[20]测定被 CCl_4 诱导急性肝损伤大鼠血清中丙氨酸氨基转移酶(ALT)、天冬氨酸氨基转移酶(AST)、总胆红素(TBIL)水平,发现连翘苷元可明显降低血清中 ALT、AST 和 TBIL 水平,由此说明连翘苷元具有保肝作用。连翘苷元保肝的作用机制与其增加肝组织中抗氧化酶的活性、降低脂质过氧化水平、降低 TNF-α、IL-8 等促炎因子水平有关。

杨美兰等[21]采用胆管引流法,观察连翘酯苷对正常麻醉大鼠胆汁流量的影响,并分别测定胆汁中主要成分,结果表明,连翘酯苷可显著增加麻醉大鼠胆汁的流量,呈现比较明显的量效关系,但对胆汁成分影响不明显,说明连翘酯苷有明显的利胆作用。

2.2.8 其他作用。连翘还有调节免疫[22]、镇吐止呕[23]、利尿降压[24]、降血脂[25]等作用。

2.3 临床应用

连翘是我国临床常用中药之一,具有抗菌、抗病毒、抗炎等作用,常被用来治疗急性风热感冒、痈肿疮毒、尿路感染等症,为连花清瘟胶囊、维 C 银翘片、双黄连口服液、清热解毒口服液等中成药或中药制剂的主要原料[26]。

2018 年国家卫生计生委发布了《流行性感冒治疗方案》,将连花清瘟胶囊和维 C 银翘片等列为流感推荐用药,为中成药的发展提供了巨大的平台,也为中成药更广泛的临床应用提供了方向。

3 栽培现状

连翘在我国使用历史虽然悠久,但人工规模化种植极为少见,多数都是仿野生种植,管理比较粗放,技术不得要领,产量和质量均不稳定,影响整个连翘产业的规模化发展[27]。因此,有必要对其栽培方面进行研究和开发,以发挥其更大的作用。

3.1 育苗技术

连翘育苗通常采用种子繁殖育苗和扦插育苗 2 种。近年来,随着农业技术的发展,容器育苗技术已应用到连翘育苗中,可显著缩短连翘培育时间,减少水分消耗,提高移栽成活率,并且通过与雨水集蓄系统的连结,适合在干旱荒山地区大面积推广应用。

3.2 田间管理

田间管理是生产高品质药用连翘的关键之一。然而很多药农对连翘的田间管理也是一知半解,包括灌水、施肥、松土、采收不得其时,整形修剪、病害防治、加工不得其法,从而导致连翘产量较低,质量不稳定。田敏利等[28]明确指出,连翘田间管理的时间和方法,期望通过规范化田间管理,实现从“田间到临床”的全过程质量监管。

3.3 环境因子对栽培的影响

不同的环境因子对连翘的生长发育有着不同影响,同时也影响其分布区域。因此,要明确其适宜范围,从而为不同区域环境适应性措施的探索提供科学依据,也为不同地区连翘栽培提供参考。

3.3.1 温度和降水。杨 洋等[29]利用 BioMod2 程序包提供的广义线性模型(GLM)、随机森林模型(RF)、推进式回归树模型(GBM/BRT)及人工神经网络模型(ANN)4 种模型得出,连翘最适宜的生长环境为年均温 12.1~17.3 ℃,年降水量 400~1 000 mm,并认为温度和降水是影响连翘分布的主导环境因子。

3.3.2 坡面和海拔。不同山地海拔高度和坡面对连翘的枝、叶、花、果的生长发育都有着不同的影响。阳坡、阴坡日积累温差近 5 ℃,阴坡花期晚于阳坡,且花期延长,落花率降低,坐果率提高,从而提高产量。不同海拔对叶体积快速生长期的影响不显著,但随着海拔的增加,短花柱类型叶纵径的相对生长速度较快[30]。野生连翘在人工抚育下,阴坡和阳坡连翘的株高、分枝数均低于未抚育的野生连翘,但连翘冠幅、结实量显著大于未抚育的野生连翘,且阴坡经人工抚育后的连翘结实量显著高于阳坡[31]。由此可以看出,阴坡更加适合连翘的栽培。

(C)1994-2019 China Academic Journal Electronic Publishing House. All rights reserved. http://www.cnki.net

3.3.3 肥料养分。合理施肥可以促进连翘树新梢生长，也可增加连翘叶叶绿素含量，提高光合作用，增加有机物的积累，从而提高连翘产量。连翘花期施用磷酸二铵和喷施磷酸二氢钾800倍液，可显著提高坐果率和干果重，单株果实增产47.5%[32]。

3.4 质量影响因素

3.4.1 不同部位有效成分不同。李丹凤等[33]对连翘不同部位活性成分含量进行分析研究，结果发现，连翘酯苷A含量青翘>老翘>连翘叶>连翘花；连翘叶中连翘苷和芦丁含量最高；连翘花中(+)-松脂素-β-D-葡萄糖苷含量最高。程启斌等[34]对连翘不同部位总酚含量的测定结果表明，连翘总酚含量连翘叶>连翘花>青翘>老翘，其认为连翘叶和花中的总酚含量高的原因可能是细胞内含较多叶绿素，通过光合作用转化成大量类黄酮类多酚化合物——花青素，而果实中多以油脂性成分为主。连翘不同部位有效成分不同，可以利用这一点开发不同产品，扩大连翘的使用范围，避免资源的浪费。

3.4.2 采收时间。近些年，由于连翘需求量增大，药农在利益的驱使下，对连翘进行"抢青"采摘，此时的连翘并未成熟，有效成分积累不够，导致连翘资源浪费和药材质量下降。崔旭盛等[35]对涉县连翘适宜采收期进行了研究，发现7月中下旬采摘的连翘，各项指标都符合《中国药典》的规定，且浸出物、连翘苷和连翘酯苷的含量相对较高，故认为连翘青翘最佳的采收期为7月中下旬。闫 瑞等[36]对"抢青"采收和正常采收的青翘进行了比较，发现"抢青"采收虽然连翘酯苷A是正常采收的1.32倍，连翘苷是正常采收的1.42倍，但千粒重却仅是正常采收的0.26倍。

3.4.3 产地。中药自古就有道地药材的说法，道地药材就是突出产地的重要性。郑爱群[37]比较了陕西黄龙和陕南汉中的连翘中连翘酯苷A的含量，发现无论是青翘还是老翘，黄龙地区的都明显高于汉中地区的。魏 珊等[38]利用气相色谱对不同产地的连翘挥发油的主要成分进行测定，发现山西地区的含量明显高于河南、河北和陕西等地区，山西太原的出油率最高，绛县和安泽的月桂烯含量最高，左权的对伞花烃含量最高，安泽的柠檬烯含量最高，晋城的α-松油醇含量最高。刘红莉等[39]对陕西商洛地区连翘中连翘酯苷A的含量测定发现，同一地区不同县城连翘酯苷A的含量各不相同，即使是同一县城不同地方，连翘酯苷A的含量也不相同。由此可以看出，产地对连翘质量影响的重要性。

4 开发利用

连翘在我国分布最广，数量最多，使用历史也最为悠久。连翘除药用外，在园林绿化、水土保持、食品、化妆品等方面也广泛应用。

连翘树生长旺盛，树形婀娜多姿，且花开周期长、开花数量多，是观光农业和现代园林难得的优良树种。连翘根系发达，可牵拉和固着土壤，增强土壤吸水和抗冲能力，是国家推荐的防治水土流失的最佳经济作物之一。连翘枝条质地柔韧，可编制成工艺品。连翘花既可以抑制酪氨酸酶活性也可以提取纯天然植物色素黄色素，可作为高级食品添加剂和化妆品使用[40-41]。王亚恒等[42]将连翘叶开发成了速溶保健茶，扩大了连翘的应用范围，避免了资源的浪费。连翘籽含油率达25%~33%，既可制成优质食用油，也可制成良好的工业原料[43]。总之，可以说连翘一身都是宝，且应用广泛，是一种值得大力开发和保护的中药材。

5 参考文献

[1] 国家药典委员会.中华人民共和国药典(一部)[S].北京：中国医药科技出版社，2015：170-171.
[2] 李英霞，孟庆梅.连翘的本草考证[J].中药材，2002，25(6)：435-437.
[3] 付云飞，李清，毕开顺.RP-HPLC法同时测定不同产地连翘中的7种成分[J].中草药，2013，44(8)：1043-1046.
[4] 罗彬，张进忠.连翘提取物化学成分研究[J].中国实验方剂学杂志，2013，19(3)：143-146.
[5] 阎新佳，温静，项峥，等.连翘的化学成分研究[J].中草药，2017，48(4)：644-647.
[6] 苏红昌，万红叶，刘翠玲，等.连翘酯苷A对酵母致热小鼠体温及TRPA1的影响[J].中国实验方剂学杂志，2016，22(1)：134-138.
[7] 党珏，袁岸，罗林，等.连翘提取物和连翘挥发油对酵母致热大鼠的解热机制研究[J].天然产物研究与开发，2017，29(9)：1542-1545.
[8] KANG H S，LEE J Y，KIM C J.Anti-inflammatory activity of arctigenin from Forsythiae Fructus[J].J Ethnopharmacol，2008，116：305.
[9] 全云云，袁岸，龚小红，等.连翘抗炎药效物质基础筛选研究[J].天然产物研究与开发，2017，29(3)：435-438.
[10] 刘玉婕，王长福，齐彦，等.金银花、连翘及其不同比例配伍抗MDR-MRSA总活性部位的体外筛选研究[J].中医药信息，2016，33(4)：14-16.
[11] 段林建，胡伶清，张清，等.不同连翘制剂对甲型流感病毒NP基因转染后表达的影响[J].中医学报，2015，30(1)：71-73.
[12] 李兴泰，李洪成，刘泽.连翘花醇提物保护线粒体及抗氧化研究[J].中成药，2009，31(6)：839-843.
[13] 田叶，田艳花，张立伟.连翘叶清除亚硝酸盐和阻断亚硝胺形成及抗氧化活性研究[J].化学研究与应用，2017，29(3)：390-395.
[14] 李寒冰，刘亚敏，吴宿慧，等.基于抗氧化活性与有效成分关联分析的连翘商品规格品质评价[J].中国医院药学杂志，2017，37(23)：2368-2373.
[15] WANG H M，WANG L W，LIU X M，et al.Neuroprotective effects of forsythiaside on learning and memory deficits in senescence-accelerated mouse prone (SAMP8)mice[J].Pharmacol Biochem Behav，2013，105(7)：134-141.
[16] KIM J M，KIM S，KIM D H，et al.Neuroprotective effect of forsythiaside against transient cerebral global ischemia in gerbil[J].Eur J Pharmacol，2011，660(2-3)：326-333.
[17] IIZUKA T，NAGAI M.Vasorelaxant effects of forsythiaside from the fruits of Forsythia suspense[J].Yakugaku Zasshi，2005，125(2)：219-224.
[18] 毛威.连翘化学成分及其抗肿瘤活性的研究[D].武汉：湖北中医学院，2009：29.
[19] 孙士萍，李磊，戴素丽，等.连翘根醇提物对食管癌移植瘤生长的体内抑制作用[J].肿瘤，2015，35(1)：1-7.
[20] 冯芹，夏文凯，王现珍，等.连翘苷元对四氯化碳大鼠急性肝损伤的保护作用[J].中国药理学通报，2015，31(3)：426-430.
[21] 杨美兰，王光建，吴永丰.连翘酯苷对大鼠胆汁流量和主要成分的影响[J].河南中医，2011，31(5)：478-479.
[22] 傅颖珺，袁娟丽，陈江，等.连翘对严重烧伤大鼠外周血Treg及脾脏Foxp3的影响[J].细胞与分子免疫学杂志，2009，25(10)：935.
[23] 聂克，朱学萍.连翘镇吐止呕作用的初步实验研究[J].山东中医药大学学报，2009，33(6)：537.
[24] 袁岸，赵梦洁，李燕，等.连翘的药理作用综述[J].中药与临床，2015，6(5)：56-59.
[25] 赵咏梅，李发荣，杨建雄，等.连翘苷降血脂及抗氧化作用的实验研究[J].天然产物研究与开发，2005，17(2)：157.
[26] 张萌，富志军，林以宁.连翘有效成分的吸收与代谢研究进展[J].亚太传统医药，2013，9(5)：58-61.
[27] 于磊，孙晓薇，钱世江，等.连翘栽培技术[J].中国林副特产，2009，5(12)：49-50.
[28] 田敏利，崔旭盛，田清存，等.涉县连翘生产标准操作规程(SOP)[J].

(下转第64页)

(C)1994-2019 China Academic Journal Electronic Publishing House. All rights reserved. http://www.cnki.net

的17%。

2.2 华亭县晚霜冻的时空变化特征

2.2.1 华亭县终霜日的年际变化特征。1971—2017年47年来，终霜日年际波动较大，并未呈现出明显的线性或多项式变化趋势，终霜日期围绕平均值上下振荡，说明华亭县终霜日并无明显的提前或推迟。

2.2.2 华亭县晚霜冻日数的年际变化特征。1971—2017年47年来，华亭县晚霜冻日数减少了10 d左右；分别分析轻霜冻和重霜冻变化日数可知，轻霜冻日数变化呈现显著的线性变化趋势，而重霜冻日数总体上虽然也呈现较为显著的线性下降趋势（通过0.05水平的显著性检验），但是其年际波动幅度较大。

2.2.3 华亭县晚霜冻的空间分布特征。华亭县晚霜冻日数呈现明显的地区差异性，年平均晚霜冻日数总体上呈现出由东北至西南逐渐递减的趋势，但也表现出明显的局地性。晚霜冻出现日数与地形、海拔高度密切相关。总的来说，晚霜冻日数分布总体由南到北依次递减，但由于地形原因，又呈现出“两边高、中间低”的分布[5]。

2.3 华亭县晚霜冻的异常特征

从表2可以看出，47年来华亭县特早终霜日只出现过1次，发生频率仅为2%。偏早终霜日出现日数最高，共发生7次，占总年份的15%，偏晚终霜日总共有4次，偏晚终霜日出现较为均匀，其中20世纪70、80、90年代、21世纪10年代各出现1次。47年来特晚霜冻共出现3次，其中20世纪90年代就发生2次，1997年终霜日出现在5月30日，为华亭县最晚终霜日。

表2 华亭县晚霜冻终霜日异常特征分布

异常类型	指标	出现年份	出现频率/%
特早	04-23	1973（21）	2
偏早	04-30	1980、1984、1992、1998、1999、2009、2012	15
偏晚	05-18	1972、1985、1995、2011	9
特晚	05-25	1971、1993（58）、1997（60）	6

2.4 晚霜冻对华亭核桃的影响

从年际变化特征可以看出，虽然受全球变暖的影响，华亭县晚霜冻日数总体呈现出减少的趋势，但是晚霜冻结束日并未提前或推迟，且近年来极端气候事件加剧，晚霜冻结束日波动较大，防霜冻形势不容乐观。

3 结论

分析结果表明，华亭县平均终霜日在5月8日，最早终霜日为4月21日，最迟终霜日为5月30日。终霜日期主要集中于5月中旬和下旬。

华亭县晚霜冻每年平均发生15次，华亭县晚霜冻主要以轻霜冻为主，占总日数的比重达到80%，平均每年发生12次，重霜冻虽然每年平均发生日数较少，但是对农作物造成的危害却是巨大的。

终霜日年际波动较大，并未呈现出明显的线性或二次变化趋势，说明华亭县终霜日并无明显的提前或推迟。

华亭县晚霜冻出现日数以2.18 d/10年的速率减少。47年来，华亭县晚霜冻日数减少了10 d左右；轻霜冻日数变化先升后降，呈现显著的线性变化趋势。而重霜冻日数总体上虽然也呈现较为显著的下降趋势，但是其年纪波动幅度较大。

用M-K方法检测得知，华亭县晚霜冻日数表现总体上呈现明显的减少趋势，但是也具有明显的阶段性特征。且华亭县晚霜冻日数减少是一种气候突变现象，突变点在2008年[6]。

华亭县晚霜冻日数呈现明显的地区差异性，年平均晚霜冻日数总体上呈现出由东北至西南逐渐递减的趋势，但也表现出明显的局地性。晚霜冻出现日数与地形、海拔高度密切相关。

华亭县核桃在4—5月较易遭受晚霜冻影响，晚霜冻对核桃生长发育影响较大，轻则造成当年核桃产量、品质等大幅度下降，重则使幼苗冻死、冻伤，甚至毁种，山区更加需要注意晚霜冻所带来的不利影响。

4 参考文献

[1] 刘吉峰，丁裕国，江志红.全球变暖加剧对极端气候概率影响的初步探讨[J].高原气象，2007，26（4）：837-842.
[2] 良勋，薛登智，梁键，等.近二十年广东的霜冻气侯特征及其变化特征[J].广东气象，2000（4）：7-9.
[3] 许育良.春季低温晚霜冻对成县核桃生长的影响[J].农业灾害研究，2012（1）：44-46.
[4] 陈少勇，孙秉强.白银市霜冻气侯变化及对农业生产的影响[J].甘肃科学学报，2006，18（4）：46—49.
[5] 林倩倩，郭慧.1971—2012年平凉市霜冻变化特征分析[J].沙漠与绿洲气象，2015，9（3）：57-62.
[6] 陈乾金，张永山.华北异常初终霜冻气候特征的研究[J].自然灾害学报，1995，4（2）：33-39.

（上接第62页）

现代中药研究与实践，2015，29（4）：4-7.
[29] 杨洋，卫海燕，王丹，等.连翘潜在地理分布预测模型的比较[J].生态学杂志，2016，35（9）：2562-2568.
[30]王海莉.不同环境条件对连翘生长发育过程的影响[J].中医学报，2014，29（11）：1630-1631.
[31] 张建军.生态因子及抚育对野生连翘生长和产量的影响[J].山西农业大学学报（自然科学版），2013，33（1）：10-15.
[32] 金蒙蒙.氮磷钾配比对连翘生长特性及土壤养分影响的研究[D].保定：河北农业大学，2016.
[33] 李丹凤，李石飞，张立伟.连翘不同部位活性成分含量分析[J].山西医科大学学报，2015，46（11）：1097-1100.
[34] 程启斌，李石飞，张立伟.连翘不同部位总酚含量测定及抗氧化活性比较研究[J].化学研究与应用，2016，28（5）：610-616.
[35] 崔旭盛，李鑫，王伟，等.连翘适宜采收期研究[J].安徽农业科学，2017，45（11）：107-108.
[36] 闫瑞，杨印军，刘红卫，等.“抢青”采摘对青翘中连翘酯苷A和连翘苷含量的影响[J].中国现代中药，2016，18（5）：579-582.
[37] 郑爱群.不同产地和采集时间连翘中连翘酯苷A的含量测定分析[J].深圳中西医结合杂志，2014，24（8）：9-10.
[38] 魏珊，吴婷，李敏，等.不同产地连翘挥发油主要成分分析及抗菌活性研究[J].中国实验方剂学杂志，2016，22（4）：69-74.
[39] 刘红莉，宋小俊，姚远，等.商洛地产药材—连翘中连翘酯苷A的质量分析[J].新疆医科大学学报，2017，40（4）：509-511.
[40] 白美美，李丹凤，李石飞，等.连翘花中抑制酪氨酸酶活性成分研究[J].天然产物研究与开发，2017，29（10）：1688-1694.
[41] 张明静，李彬.连翘花黄色素超声波提取及其稳定性探究[J].湖州职业技术学院学报，2017，15（3）：85-88.
[42] 王亚恒，姚宁，王小平，等.连翘叶速溶保健茶成型工艺优选[J].中国药师，2017，20（10）：1863-1865.
[43] 刘成伦，杨雪艳.天然药物化学[M].北京：中央广播电视大学出版社，2011：112-119.

(C)1994-2019 China Academic Journal Electronic Publishing House. All rights reserved. http://www.cnki.net
