# Supplementary material for: Efficacy of Forsythia suspensa (Thunb.) Vahl on mouse and rat models of inflammation-related diseases: a meta-analysis
Source: Front Pharmacol. 2024 Mar 4;15:1288584. doi: 10.3389/fphar.2024.1288584 (PMC10946063; doi:10.3389/fphar.2024.1288584)
Supplement: Supplementary file 1 [file DataSheet1.zip › Data Sheet 1/This meta-analysis included research articles written in Chinese and we packed all these Chinese publications as the supplementary file/Liu 2021.pdf]

山西大学学报(自然科学版):46(1):208－219,2023
Journal of Shanxi University(Nat.Sci.Ed.)

DOI:10.13451/j.sxu.ns.2021038

基于网络药理学研究连翘抗炎作用机制

刘文倩[1],高耀[2],张立伟[1],李石飞[1*]

(1. 山西大学 分子科学研究所，化学生物学与分子工程教育部重点实验室，山西 太原 030006；
2. 山西大学 中医药现代研究中心，山西 太原 030006)

摘　要:文章基于网络药理学研究连翘抗炎作用机制。通过TCMSP数据库收集连翘化学成分,并以OB≥30%,DL≥0.18筛选连翘化学成分。采用PharmMapper数据库预测连翘作用靶点。以“inflammation”为关键词使用OMIM数据库和TTD数据库检索炎症相关靶点,并用Cytoscape 3.8.1软件构建“活性成分-靶点”网络。通过String数据库进行蛋白质相互作用分析,并使用Cytoscape 3.8.1软件构建蛋白相互作用网络。最后使用DAVID数据库对连翘抗炎作用靶点进行通路富集分析,以探究连翘抗炎的作用机制。结果为经筛选得到良好的连翘活性成分21个,预测到45个潜在的抗炎靶点。其中连翘抗炎的主要靶点为丝裂原激活蛋白激酶14(MAPK14)、SRC蛋白激酶(SRC)、胰岛素样生长因子1(IGF1)、丝裂原激活蛋白激酶1(MAPK1)及表皮生长因子受体(EGFR)等核心靶点。经GO分析和KEGG通路分析,连翘参与的抗炎信号通路主要有癌症通路、催乳激素信号通路、结核病、破骨细胞分化、丙型肝炎、PI3K-Akt信号通路等。本研究得连翘的抗炎作用潜在机制可能是槲皮素,连翘苷,木犀草素等活性化合物通过作用于SRC、MAPK14、MAPK1、IGF1、EGFR等靶点调控多条信号通路发挥作用。

关键词:网络药理学;连翘;抗炎;作用机制

中图分类号:R285.5　　**文献标志码**:A　　**文章编号**:0253-2395(2023)01-0208-12

Study on Anti-inflammatory Mechanism of Forsythiae Fructus Based on Network Pharmacology

LIU Wenqian[1], GAO Yao[2], ZHANG Liwei[1], LI Shifei[1*]

(1. *Key Laboratory of Chemical Biology and Molecular Engineering of Ministry of Education, Institute of Molecular Science, Shanxi University, Taiyuan* 030006, *China;*
2. *Modern Research Center for Traditional Chinese Medicine, Shanxi University, Taiyuan* 030006, *China*)

Abstract: The anti-inflammatory mechanism of Forsythiae Fructus has been studied based on network pharmacology. In this work, chemical constituents of Forsythiae Fructus were collected through TCMSP database, and the chemical constituents of Forsythiae Fructus were screened with OB≥30% and DL≥0.18. The PharMapper database was used to predict the targets of Forsythiae Fructus. The OMIM database and TTD database were used to search the targets of inflammation with "inflammation" as the keyword. And Cytoscape 3.8.1 software was used to construct the network of "active ingredient-target". The protein interaction analysis was carried out through the String database. And the Cytoscape 3.8.1 software was used to construct the protein interaction network. Finally, the DAVID database was used to conduct pathway enrichment analysis on the anti-inflammatory targets of Forsythiae Fructus to explore the anti-inflammatory mechanism of Forsythiae Fructus. In total, 21 good active ingredients and 45 anti-inflammatory targets has passed through screening. The main anti-inflammatory targets of Forsythiae Fructus are MAPK14, SRC, IGF1, MAPK1,

收稿日期:2021-02-07;**接受日期**:2021-06-04
基金项目:国家中药标准化项目(ZYBZH-Y-JIN-34);国家自然科学基金(31800293)
作者简介:刘文倩(1996-),女,山西大同人,硕士研究生,主要研究方向为天然药物化学。E-mail:2313478677@qq.com
* **通信作者**:李石飞(LI Shifei),E-mail:lisf@sxu.edu.cn;
引文格式:刘文倩,高耀,张立伟,等.基于网络药理学研究连翘抗炎作用机制[J].山西大学学报(自然科学版),2023,46(1):208-219.DOI:10.13451/j.sxu.ns.2021038

(C)1994-2023 China Academic Journal Electronic Publishing House. All rights reserved.　http://www.cnki.net

EGFR, etc. After GO analysis and KEGG pathway analysis, Forsythiae Fructus is mainly involved in pathways in cancer, prolactin signaling pathway, tuberculosis, osteoclast differentiation, hepatitis C, PI3K-Akt signaling pathway, etc. The anti-inflammatory ingredients of Forsythiae Fructus may be quercetin, forsythin, luteolin, etc. They regulate multiple signal pathways by acting on targets such as SRC, MAPK14, MAPK1, IGF1, EGFR, etc.

Key words: network pharmacology; Forsythiae Fructus; anti-inflammatory; mechanism

0 引言

中药连翘是木犀科植物连翘*Forsythia suspensa*（Thunb.）Vahl的干燥果实，主要分布于我国的山西、陕西，河南和山东等地。连翘气微香、味苦、性微寒、归肺心、小肠经，具清热解毒，消肿散结，疏散风热等效用，是有着千年历史的传统中药[1]。连翘散结消肿、清热解毒的药效在多方面得到体现，主要包括抗炎、抗肿瘤、抗病原微生物、解热等。连翘被广泛运用于临床，是许多中药复方制剂的重要成分，如连花清瘟胶囊，双黄连含片，牛黄上清片等[2-3]。

炎症作为一种常见而又十分复杂的病理现象，它是机体对刺激及损伤产生的一种防御反应。炎症一般对机体是有益的，但严重的炎症亦会伤害机体，炎症与多数疾病都有密切的联系[4-5]。连翘具有较强的抗炎作用，临床上用于治疗炎症相关疾病，如急性风热感冒，痈肿疮毒等[6]。对于连翘化学成分的相关研究，已知从连翘中可分离出达300多种化学成分，主要化学成分多达150多种，包含有黄酮类、木脂素类、苯乙醇苷类以及挥发油类等。其中连翘苷，槲皮素，木犀草素等均报道有良好的抗炎活性[2,5]。相关文献报道，厉世伟等研究连翘有效成分对炎症模型的作用，结论得连翘提取物对炎性细胞有不同程度保护，具有一定的抗炎作用[4]。王越等研究连翘成分连翘苷对LPS刺激的BV2小胶质细胞炎性反应的抑制作用，结果表明连翘苷能够抑制小胶质细胞活化产生的炎症反应[7]。陈泽文等研究连翘不同极性部位的抗炎活性，结果表明连翘的部分提取物具有良好的抗炎活性[8]。目前关于连翘抗炎的研究有很多，大多研究了连翘的各种成分表现出的抗炎活性，然而在系统阐明连翘抗炎具体作用机制及相关作用等方面的研究相对缺乏。因此我们需要进一步加强对连翘抗炎作用机制的研究。

中药注重整体性，并在功效上表现多样性特点。其分子机制相对复杂，难以深入研究。而网络药理学是一门将信息网络与计算机科学相结合，基于系统生物学和多向药理学理论的新学科，其特点具整体性和系统性，与中医药整体性原则相吻合[9-10]。从网络药理学这门技术上可以获得“多成分-多靶点”的相互联系及蛋白互作关系，预测中药及其分子作用机制，使得中药研究得以更好延伸。本研究以连翘抗炎作出发点，通过网络药理学的方法来研究连翘抗炎的作用机制，为连翘后期研究提供相关理论依据。

1 材料与方法

1.1 连翘活性成分收集与筛选

本研究经中医药系统药理学数据库TCMSP（http://tcmspw.com/tcmsp.php）检索连翘的活性成分信息。口服药物在体内经过吸收（absorption）、分布（distribution）、代谢（metabolism）及排泄（excretion）的过程，为ADME过程。口服生物利用度（OB，oral bioavailability）是衡量中药临床药效的有效指标，类药性原则（DL，drug likeness）是指化合物与已知药物的相似性。所以本研究以OB≥30%及DL≥0.18的2个条件进行筛选以获得有效的活性成分[11-12]。

1.2 连翘活性成分作用靶点的预测与筛选

将TCMSP数据库筛选得到活性成分的化学结构数据导入PharmMapper（http://lilab-ecust.cn/pharmmapper/）平台进行预测靶点，再将每个成分的预测结果的Z值进行降序，分别选择前50个结果进行后续研究[12]。并使用Uniprot蛋白质数据库（https://www.uniprot.org）规范靶点信息。

1.3 炎症靶点的收集与筛选

通过OMIM数据库（https://omim.org/）和TTD数据库（https://db.idrblab.org/ttd/）检索炎症靶点，以“inflammation”为关键词进行检索，

(C)1994-2023 China Academic Journal Electronic Publishing House. All rights reserved. http://www.cnki.net

其中OMIM数据库选带有“*”的炎症靶点。收集两个数据库与炎症相关的靶点信息并进行整合。再将预测靶点与炎症靶点取交集得到共同靶点,并绘制韦恩图。

1.4 构建“连翘成分-预测靶点”和“连翘成分-炎症靶点”网络图

将连翘的活性成分,预测靶点,交集的炎症靶点建成EXCEL数据,将其导至Cytoscape 3.8.1(http://www.cytoscape.org/)软件中,利用该软件构建“连翘成分-预测靶点”,“连翘成分-炎症靶点”,以探究连翘抗炎作用机制。运用“Network analyzer”功能分析相关属性,导出接近中心性ClosenessCentrality,中介中心性BetweennessCentrality,点度中心性Degree等参数,Degree为每一个节点拥有边的数量,一个节点的Degree越大,说明该节点在网络中就越重要。

1.5 蛋白靶点PPI(Protein-Protein Interaction)网络的构建

取交集靶点导入到String平台(https://string-db.org),生物种类设为人类,其余为默认设置,得到蛋白互作(PPI)网络,再利用CytoScape3.8.1软件中的MCODE插件进一步分析PPI网络,得到蛋白互作关系的高密度子网[13]。

1.6 连翘抗炎靶点的功能与通路富集分析

将交集靶点上传至DAVID数据库,进行GO(Gene Ontology)功能和KEGG(Kyoto Encyclopedia of Genes and Genomes)通路富集分析。所得结果以$P<0.05$为界,GO分析选取前10个富集结果和KEGG分析选取前20个富集结果并利用R语言进行可视化,并构建“连翘抗炎靶点-通路”网络图。

2 结果

2.1 连翘活性成分及成分靶点的获取

经TCMSP数据库获得150种连翘活性成分,再经OB≥30%及DL≥0.18筛选后获得21个连翘活性成分。21个成分分为8种类型结构,分别为:7个木脂素类及其苷类,4个黄酮类,1个C6-C2天然醇及其苷类,2个甾醇类,4个萜类,1个醌类,1个有机酸类,1个生物碱物质,详见表1。21个活性成分经PharmMapper平台预测靶点并筛选,再删除重复靶点得254个靶点,最后又经Uniprot数据库规范得230个。

2.2 炎症靶点的获取

通过OMIM数据库输入“inflammation”关键词得1091个炎症靶点,又经筛选带有“*”符号的靶点获得818个炎症靶点。另外使用TTD数据库输入“inflammation”得120个炎症靶点。最后整合得871个炎症靶点。将所得的连翘活性成分预测靶点与炎症靶点取交集,得到交集靶点45个,并绘制韦恩图,如图1所示。

2.3 构建“连翘活性成分—靶点”网络图

使用Cytoscape3.8.1软件构建“连翘活性成分-预测靶点”网络图(共251个节点,其中21个成分节点和230个预测靶点;共949条边),绿色六边形为21个成分,黄色和蓝色菱形为230个靶点,其中黄色为度值≥11的靶点,如图2所示。在该网络中,分析其拓扑学参数,其中有6个预测靶点可以与15个及以上的化合物相互作用。度值≥15的靶点有碳酸酐酶-2(Carbonic anhydrase 2),细胞分裂蛋白激酶2(Cell division protein kinase 2),酪氨酸蛋白磷酸酶非受体1型(Tyrosine-protein phosphatase non-receptor type 1),凝血酶原(Prothrombin),雄激素受体(Androgen receptor),热休克蛋白HSP 90-alpha(Heat shock protein HSP 90-alpha),详见表2。由上述可知,连翘的有效活性成分与相应靶点之间存在一个成分对应多个靶标、一个靶标对应多个成分的关系,这体现了连翘多成分、多靶点共同作用的机制。

构建“连翘活性成分-炎症靶点”网络图(共66个节点,其中21个成分节点和45个炎症靶点;共211条边),绿色菱形为连翘21个活性成分,淡粉色方形为45个炎症靶点。分析网络图可以得到连翘抗炎的核心成分,网络分析表明:其中有8个活性成分的度值在11及以上,依次为MOL000098(槲皮素),MOL003281(20(S)-达玛烷-24-烯-3β,20-二醇-3-乙酸酯),MOL003305(连翘苷),MOL003322(4-[(3R,6S)-3-(3,4-二甲氧基苯基)-1,3,3a,4,6,6a-六氢呋喃[3,4-c]呋喃-6-基]-3-甲氧基苯酚),MOL003283((+)-异落叶松树脂醇),MOL003344(β-香树脂醇乙酸酯),MOL000006(木犀草素),MOL003308((+)-松脂醇单甲醚-4-

(C)1994-2023 China Academic Journal Electronic Publishing House. All rights reserved. http://www.cnki.net

表1 连翘主要活性成分

Table 1 The main active ingredients of Forsythiae Fructus

MOL ID	Molecule Name	OB/%	DL	Chemical strcuture type
MOL000173	Wogonin<br>汉黄芩素	30.68	0.23	黄酮类
MOL003281	20(S)—Dammar—24—ene—3β,20—diol—3—acetate<br>20(S)—达玛烷—24—烯—3β,20—二醇—3—乙酸酯	40.23	0.82	萜类
MOL003283	(2R,3R,4S)—4—(4—hydroxy—3—methoxy—phenyl)—7—methoxy—2,3—dimethylol—tetralin—6—ol<br>(+)—异落叶松树脂醇	66.51	0.39	木脂素类及其苷类
MOL003290	(3R,4R)—3,4—bis[(3,4—dimethoxyphenyl)methyl]oxolan—2—one<br>二甲基罗汉松脂素	52.30	0.48	木脂素类及其苷类
MOL003295	(+)—Pinoresinol monomethyl ether<br>松脂素单甲基醚	53.08	0.57	木脂素类及其苷类
MOL003305	Phillyrin<br>连翘苷	36.40	0.86	木脂素类及其苷类
MOL003308	(+)—pinoresinol monomethyl ether—4—D—β—glucoside<br>(+)—松脂素单甲基醚—4—D—β—葡萄糖甙	61.20	0.57	木脂素类及其苷类
MOL003315	3β—Acetyl—20,25—epoxydammarane—24α—ol<br>3β—乙酰基—20,25—环氧达玛烷—24α—醇	33.07	0.79	萜类
MOL000211	Betulinic acid<br>白桦脂酸	55.38	0.78	萜类
MOL003322	Forsythinol<br>4—[(3R,6S)—3—(3,4—二甲氧基苯基)—1,3,3a,4,6,6a—六氢呋喃[3,4—c]呋喃—6—基]—3—甲氧基苯酚	81.25	0.57	C6—C2天然醇及其苷类
MOL003330	(—)—Phillygenin<br>连翘脂素	95.04	0.57	木脂素类及其苷类
MOL003344	β—Amyrin acetate<br>β—香树脂醇乙酸酯	42.06	0.74	萜类
MOL003348	Adhyperforin<br>加贯叶金丝桃素	44.03	0.61	醌类
MOL003365	Lactucasterol<br>胆甾—28—甲基—23,24—环丙烷—Δ^5—4—酮	40.99	0.85	甾醇类
MOL003370	Onjixanthone I<br>7—羟基—1,2,3—三甲氧基蒽酮	79.16	0.30	有机酸类
MOL000358	β—Sitosterol<br>β—谷甾醇	36.91	0.75	甾醇类
MOL000422	Kaempferol<br>山奈酚	41.88	0.24	黄酮类
MOL000522	Arctiin<br>牛蒡子苷	34.45	0.84	木脂素类及其苷类
MOL000006	Luteolin<br>木犀草素	36.16	0.25	黄酮类
MOL000791	Bicuculline<br>(+)—荷包牡丹碱	69.67	0.88	生物碱
MOL000098	Quercetin<br>槲皮素	46.43	0.28	黄酮类

D-β-葡萄糖苷),如图3所示。

2.4 蛋白靶点PPI网络的构建

取交集靶点提交至String平台,得PPI网络图,经Cytoscape3.8.1软件修饰得图45个节点,216条边。度值越大,颜色越深,其平均度值9.6,如图4所示。为更精确地分析连翘抗炎的

(C)1994-2023 China Academic Journal Electronic Publishing House. All rights reserved. http://www.cnki.net

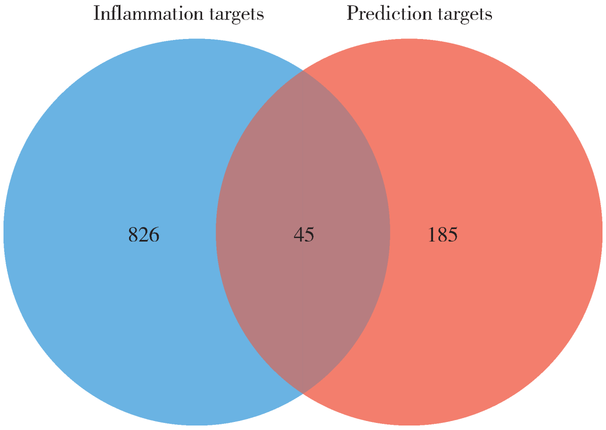

图1 预测靶点与炎症靶点的交集

Fig. 1 The intersection of inflammation targets and prediction targets

作用机制，运用Cytoscape3.8.1中的MCODE插件，得高密度子网，其靶点为45个靶点度值前14个靶点，可见度值越高，在PPI网络中越重要。由图可知，连翘抗炎主要与丝裂原激活蛋白激酶14(MAPK14)，SRC蛋白激酶(SRC)，胰岛素样生长因子1(IGF1)，丝裂原激活蛋白激酶1(MAPK1)，表皮生长因子受体(EGFR)，丝裂原激活蛋白激酶8(MAPK8)，白细胞介素2(IL2)，信号转导与转录激活因子1(STAT1)，90KDA热休克蛋白AA1(HSP90AA1)，基质金属蛋白酶2(MMP2)等靶点密切相关，如图5所示。

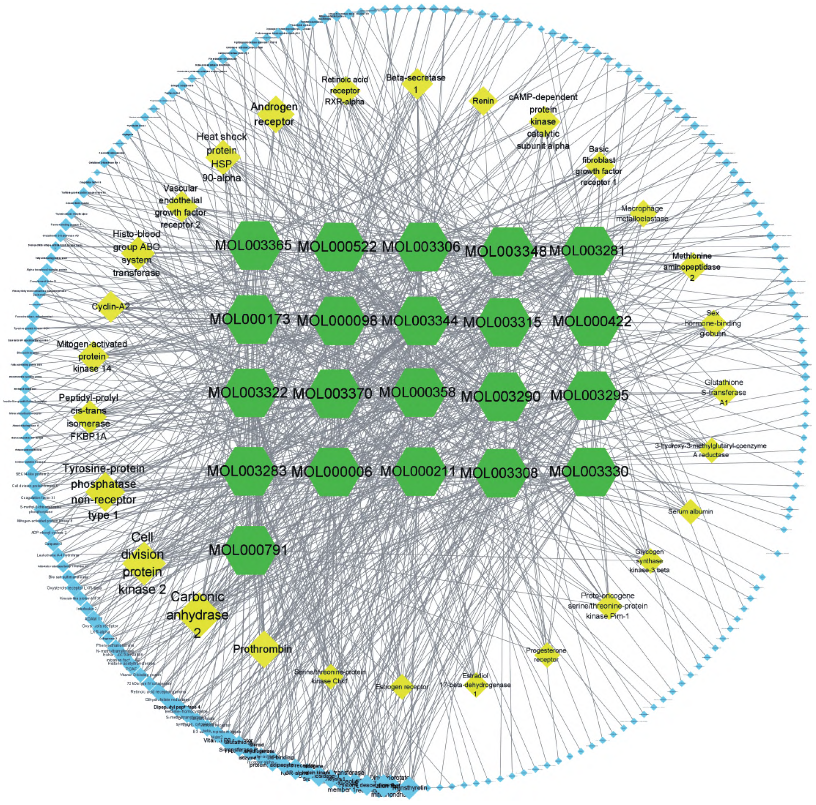

图2 连翘活性成分-预测靶点网络图

Fig. 2 The Forsythiae Fructus's active ingredients-predicted targets network diagram

表2 活性成分-预测靶点网络的关键靶点及其拓扑学性质

Table 2 Active ingredients-the key targets of the predicted target network and their topological properties

Name	Closeness Centrality	Betweenness Centrality	Degree
Carbonic anhydrase 2	0.521 739 130 434 782 6	0.046 492 499 813 446 98	20
Cell division protein kinase 2	0.515 337 423 312 883 5	0.044 790 771 063 967 29	19
Tyrosine-protein phosphatase non-receptor type 1	0.493 150 684 931 506 9	0.030 861 297 799 180 88	17
Prothrombin	0.462 385 321 100 917 46	0.022 471 810 371 072 907	16
Androgen receptor	0.450 805 008 944 543 8	0.019 355 294 091 387 13	16
Heat shock protein HSP 90-alpha	0.457 350 272 232 304 96	0.019 060 681 116 201 482	15

(C)1994-2023 China Academic Journal Electronic Publishing House. All rights reserved. http://www.cnki.net

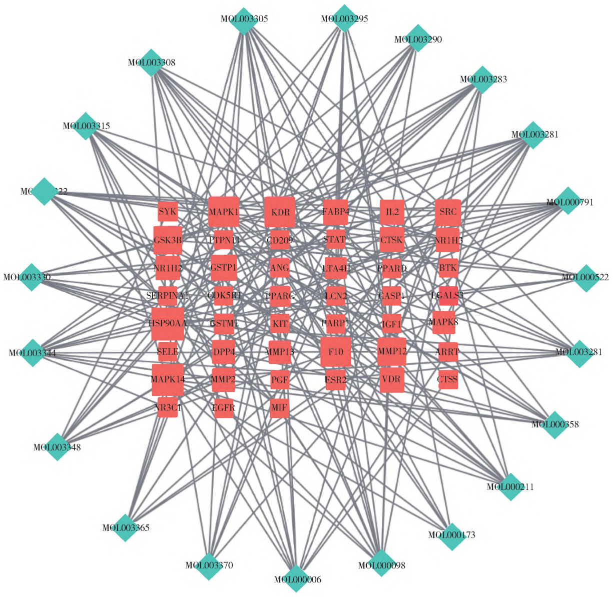

图3 连翘活性成分-炎症靶点网络图

Fig. 3 The Forsythiae Fructus's active ingredients-inflammation target network diagram

2.5 连翘抗炎靶点功能与通路的富集分析

经分析并借助R语言对结果可视化得:连翘参与的生物学过程(BP,Biological Process)主要有细胞对脂多糖的反应(Cellular response to lipopolysaccharide),Peptidyl-serine 磷酸化(Peptidyl-serine phosphorylation),细胞对胰岛素刺激的反应(Cellular response to insulin stimulus),凋亡过程的负调控(Negative regulation of apoptotic process),来自RNA聚合酶Ⅱ启动子转录起始(Transcription initiation from RNA polymerase Ⅱ promoter)等过程。连翘主要参与的细胞组成(CC,Cellular Component)有细胞外间隙(Estracellular space),细胞核(Nucleus),细胞质核周区域(Perinuclear region of cytoplasm),胞质溶胶(Cytosol),核质(Nucleoplasm)等成分。连翘抗炎靶点的功能(MF,Molecular Function)主要有类固醇激素受体活性(Steroid hormone receptor activity),蛋白质酪氨酸激酶活性(Protein tyrosine kinase activity),受体信号蛋白酪氨酸激酶活性(Receptor signaling protein tyrosine kinase activity),酶结合(Enzyme binding),蛋白结合(Protein binding)等功能,如图6所示。

此外,KEGG通路分析结果显示得相关通路有癌症通路(Pathways in cancer),催乳激素信号通路(Prolactin signaling pathway),结核病(Tuberculosis),破骨细胞分化(Osteoclast differentiation),丙型肝炎(Hepatitis C),PI3K-Akt信号通路(PI3K-Akt signaling pathway),GnRH信号通路(GnRH signaling pathway),Rap 1信号通路(Rap 1 signaling pathway),Ras信号通路(Ras signaling pathway),如图7所示,富集通路结果详见表3。最后构建抗炎靶点-通路图,以选取的20条KEGG通路及所涉及的27个抗炎靶点作图,并分析其拓扑参数。网络分析得,MAPK1、MAPK8、EGFR、MAPK14度值分别为19、14、13、12,在网络中度值占最高,为关键靶

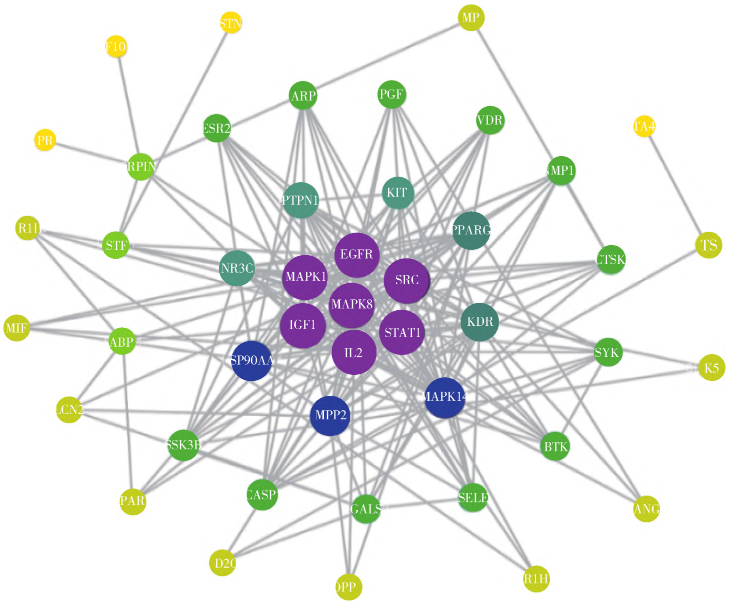

图4 交集靶点蛋白互作关系

Fig. 4 Intersection target protein interaction relationship

(C)1994-2023 China Academic Journal Electronic Publishing House. All rights reserved. http://www.cnki.net

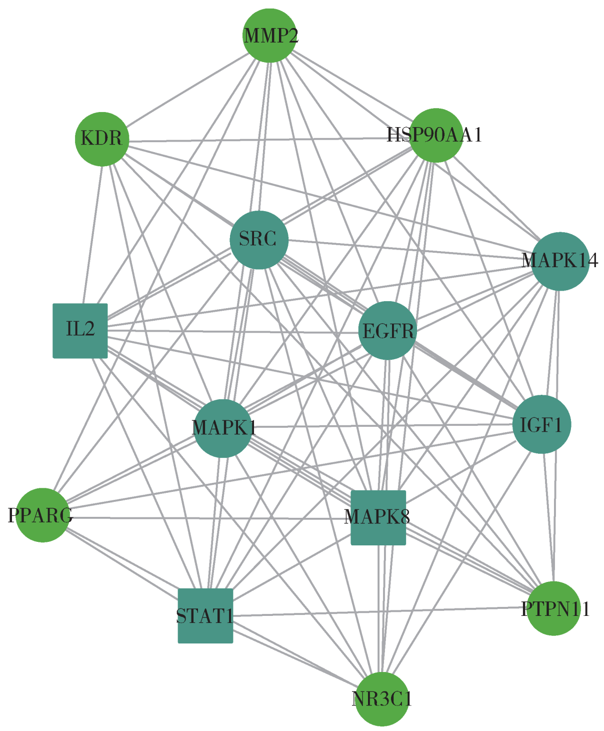

图5 PPI网络中的高密度子网

Fig. 5 High-density subnet in PPI network

点,如图8所示。

3 讨论

连翘是临床常用清热解毒要药,在风热感冒、乳痈、瘰疬、丹毒、温病初起、痈疽等热证治疗显示出显著疗效。现代药理学研究显示,连翘在抗病毒、抗肿瘤、抗炎等方面均显示出作用。其中抗炎作为连翘的主要药理作用之一,目前只是在研究连翘的不同化学成分的抗炎活性较为充分,但连翘抗炎作用机理的研究却相对缺乏。本研究利用网络药理学的方法从连翘抗炎的活性成分、作用靶点、相关通路等方面对作用机制进行了探讨。从连翘的活性成分筛选出连翘抗炎的核心成分为槲皮素、连翘苷、木犀草素等。槲皮素为多醇羟基黄酮类化合物,具有抗炎,免疫调节,抗癌,抗氧化等作用。经研究发现,槲皮素可通过抑制NF-κB通路,降低炎症因子表达,达到抗炎目的[14]。木犀草素是存在于许多药用植物中的典型C6-C3-C6结构的黄酮类化合物,且具有多种药理活性。木犀草素发挥抗炎作用,其通过影响花生四烯酸的代谢,多条炎性信号通路如NF-κB信号通路、MAPK和AP-1信号通路及抑制炎症因子表达等多种途径抗炎[15-16]。相关研究揭示了木犀草素与槲皮素在体外表现良好的抗炎效果[17]。有文献报道连翘苷对炎症因子具有抑制作用,表现出良好的抗炎活性[18-19]。

表3 KEGG富集通路

Table 3 KEGG enrichment pathway

Term	Description	Count	*P*-value
hsa05200	Pathways in cancer	13	9.78×10^{-7}
hsa04917	Prolactin signaling pathway	7	2.43×10^{-6}
hsa05152	Tuberculosis	9	5.10×10^{-6}
hsa04380	Osteoclast differentiation	8	7.16×10^{-6}
hsa05160	Hepatitis C	7	9.01×10^{-5}
hsa04151	PI3K-Akt signaling pathway	10	1.00×10^{-4}
hsa05205	Proteoglycans in cancer	8	1.10×10^{-4}
hsa04510	Focal adhesion	8	1.33×10^{-4}
hsa04912	GnRH signaling pathway	6	1.46×10^{-4}
hsa04015	Rap1 signaling pathway	8	1.50×10^{-4}
hsa04915	Estrogen signaling pathway	6	2.18×10^{-4}
hsa04014	Ras signaling pathway	8	2.36×10^{-4}
hsa04621	NOD-like receptor signaling pathway	5	2.62×10^{-4}
hsa05120	Epithelial cell signaling in Helicobacter pylori infection	5	5.23×10^{-4}
hsa04664	Fc epsilon RI signaling pathway	5	5.54×10^{-4}
hsa04914	Progesterone-mediated oocyte maturation	5	1.40×10^{-3}
hsa04012	ErbB signaling pathway	5	1.40×10^{-3}
hsa05215	Prostate cancer	5	1.46×10^{-3}
hsa05219	Bladder cancer	4	1.55×10^{-3}
hsa05164	Influenza A	6	2.80×10^{-3}

(C)1994-2023 China Academic Journal Electronic Publishing House. All rights reserved. http://www.cnki.net

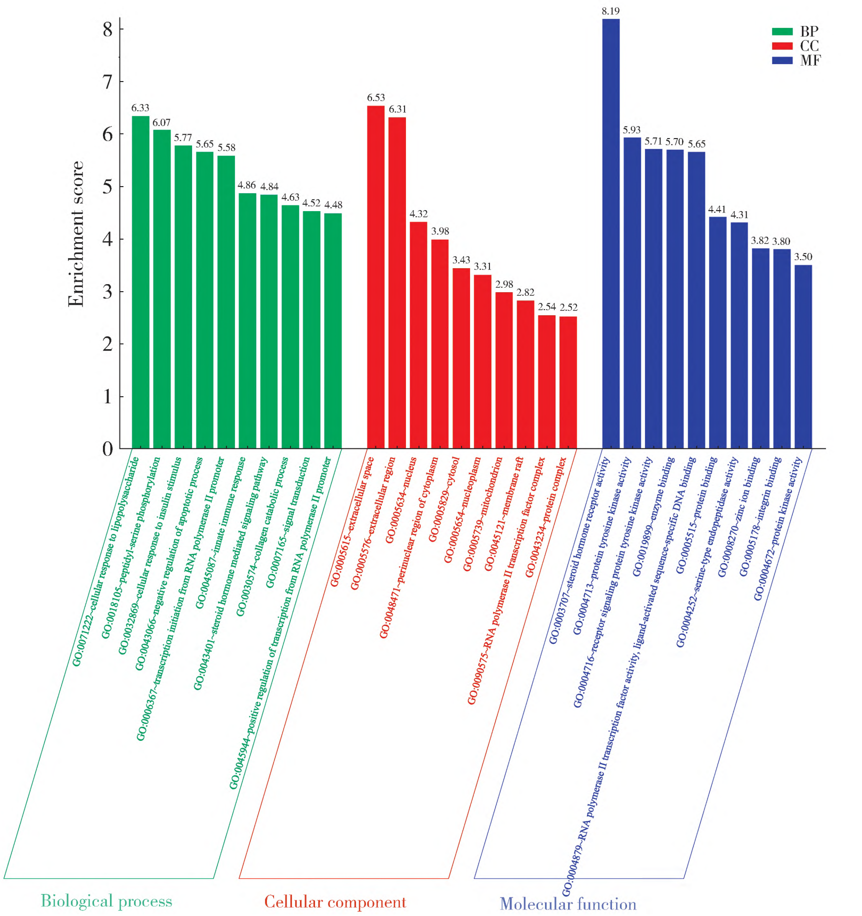

图6 GO分析的生物过程,细胞成分,分子功能

Fig. 6 The biological process, cell composition, molecular function of GO analysis

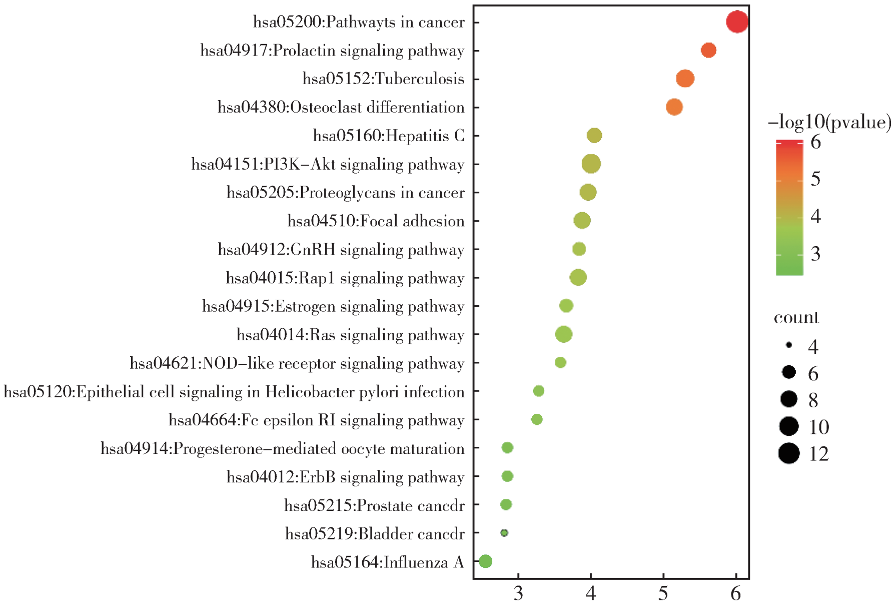

图7 KEGG通路分析

Fig. 7 KEGG pathway analysis

在连翘抗炎的蛋白相互作用网络中通过构建高密度子网,有14个蛋白靶点起关键作用,其核心靶点为MAPK14、SRC、IGF1、MAPK1、EGFR等。丝裂原活化蛋白激酶(MAPK),具

(C)1994-2023 China Academic Journal Electronic Publishing House. All rights reserved. http://www.cnki.net

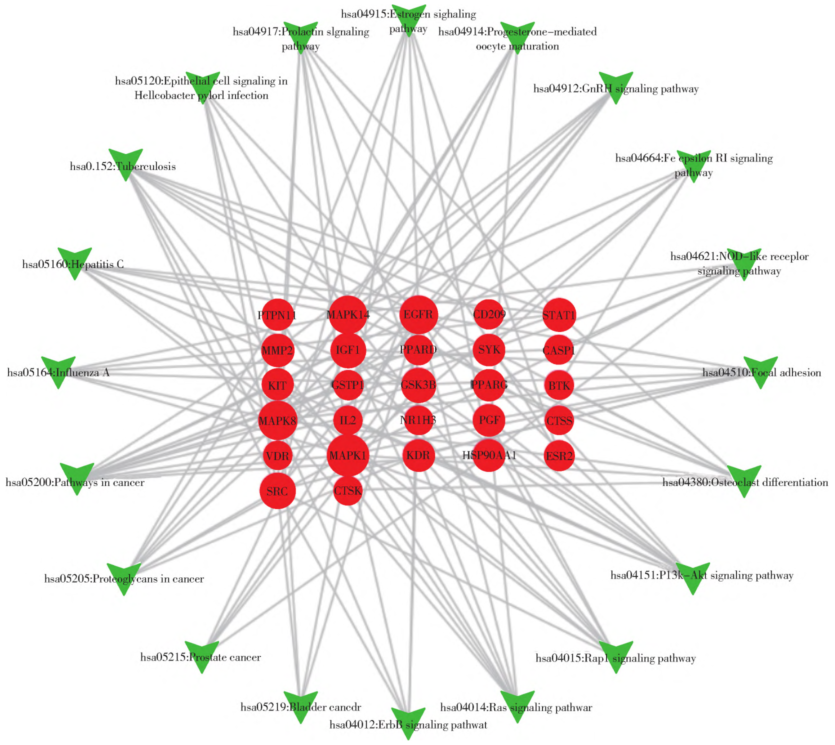

图8　抗炎靶点-通路图

Fig. 8　Anti-inflammatory target-pathway diagram

有调控促炎因子的作用。MAPK 家族可分为 p38、JNK 和 ERK,其中 p38,JNK 在炎症反应中发挥重要作用[20],MAPK14 蛋白激酶是 4 个 p38 MAPKs 之一[12]。MAPK1 是 MAPK 家族一员,参与炎症反应,它的激活可促使炎症因子的表达[21]。SRC 是一种非受体蛋白酪氨酸激酶,它参与细胞的生长、发育和分化等过程且发挥重要作用,并与肿瘤的发展密切相关[22]。IGF1 作为一种重要的生长因子,以自分泌或者是旁分泌的方式促使成骨细胞的增殖、分化、维持骨代谢的平衡,从而促进骨形成[23]。EGFR 作为一种Ⅰ型跨膜糖蛋白,经诱导形成二聚体,再磷酸化后激活下游信号通路,从而影响细胞的增殖、分化与存活[24]。

由本研究中气泡图气泡的颜色和大小,得出连翘抗炎的主要通路有癌症通路、催乳激素信号通路、结核病、破骨细胞分化、丙型肝炎、PI3K-Akt 信号通路等。癌症严重威胁着人类健康,是全球性的公共卫生问题之一[25]。炎症因子与癌症密切相关,其参与了肿瘤的发生、促进、恶性转化、侵袭及转移病理过程,并且核转录因子 NF-κB 在炎症与肿瘤之间起着重要作用[26]。Prolactin 是一种多肽激素,具有作为激素和细胞因子的生物活性功能[27]。它参与生物过程广泛,包括神经调节,泌乳,情绪反应,细胞生长增殖,内分泌以及免疫调节[28]。催乳素还通过对星形胶质细胞白细胞介素功能的旁分泌调节,在神经炎性反应相关过程中发挥关键作用[29]。结核病是一个连续体,由一系列病变组成,是炎症复杂调节的结果,促炎因子包括干扰素、TNF-α、IL-1 以及微小核糖核酸和类花生酸在结核病期间形成一个互作网络。结核病是由一种高度健壮和复杂的细菌病原体结核分枝杆菌引起的,结核病的特点是潜伏和活动期的炎症不消退。结核病是一种局部疾病,典型集中在肺,即肺结核[30-31]。目前临床主要通过抗炎、祛痰及止咳等方式治疗肺结核[32]。破骨细胞由单核细胞融合分化而成,是骨吸收的功能细胞[33],在骨骼发育和维持中起重要作用,破骨细胞异常活化时会导致各种骨相关疾病的发生。据文献报道,有许多中医药基于 NF-κB 信号通路来调控破骨细胞分化[34]。丙型肝炎是由于感染丙肝病毒(HCV)所导致。丙肝病毒是一种有包膜的 RNA 病毒,属于类肝素病毒属和黄病毒科[35]。丙型肝炎的分子机制与多条炎症信号通路相关,如

(C)1994-2023 China Academic Journal Electronic Publishing House. All rights reserved.　http://www.cnki.net

MAPK、NF-κB等信号通路[36]。PI3K-Akt途径是许多系统中细胞生长的重要调节因子，PI3K（磷酸肌醇3-激酶）家族由1类、2类和3类激酶组成，它们将PIP2（磷脂酰肌醇二磷酸）磷酸化为PIP3。1类PI3K激酶参与Akt（蛋白激酶B）的激活，Akt激活导致许多下游蛋白质的刺激，影响细胞生长、存活以及分化[37]。相关文献报道，PI3K-Akt信号通路参与炎症反应，并发挥较明显的调控作用[38-40]。

综上所述，本研究通过网络药理学方法对连翘抗炎作用机制进行了初步预测，分析了连翘抗炎相关靶点及所涉及通路，为后续研究提供了指导意义。基于上述结果，可以发现连翘在抗炎作用中是多个活性成分参与多个靶点的调控，同时一个靶点也参与调控多条信号通路，符合中药的多成分，多靶点，多种途径的特征。

参考文献：

[1] 国家药典委员会. 中华人民共和国药典-一部: 2020年版[M]. 北京: 中国医药科技出版社, 2020: 177-178.
National Pharmacopoeia Committee. Pharmacopoeia of the People's Republic of China - One: 2020 edition[M]. Beijing: China Medical Science Press, 2020: 177-178.

[2] 全云云, 袁岸, 龚小红, 等. 连翘抗炎药效物质基础筛选研究[J]. 天然产物研究与开发, 2017, **29**(3): 435-438. DOI: 10.16333/j.1001-6880.2017.3.013.
QUAN Y Y, YUAN A, GONG X H, *et al*. Investigation on Anti-inflammatory Components of *Forsythia suspensa* [J]. *Nat Prod Res Dev*, 2017, **29**(3): 435-438. DOI: 10.16333/j.1001-6880.2017.3.013.

[3] 袁岸, 赵梦洁, 李燕, 等. 连翘的药理作用综述[J]. 中药与临床, 2015, **6**(5): 56-59.
YUAN A, ZHAO M J, LI Y, *et al*. The Review of Pharmacological Effects of Lianqiao[J]. *Pharm Clin Chin Mater Med*, 2015, **6**(5): 56-59.

[4] 厉世伟, 司宁宁, 李龙, 等. 连翘有效成分对炎症模型的作用[J]. 中兽医医药杂志, 2012, **31**(1): 16-19. DOI: 10.13823/j.cnki.jtcvm.2012.01.004.
LI S W, SI N N, LI L, *et al*. Effect of *Forsythia* Active Components on Cell Inflammation Reaction Model[J]. *J Tradit Chin Vet Med*, 2012, **31**(1): 16-19. DOI: 10.13823/j.cnki.jtcvm.2012.01.004.

[5] 龚莉虹, 余琳媛, 胡乃华, 等. 连翘抗炎药效物质基础及其作用机理研究进展[J]. 中药与临床, 2019, **10**(1): 43-49.
GONG L H, YU L Y, HU N H, *et al*. Advances on the Anti-inflammatory Material Basis of Lianqiao and Its Action Mechanism[J]. *Pharm Clin Chin Mater Med*, 2019, **10**(1): 43-49.

[6] 王婷婷, 张萬, 杨志敏, 等. 连翘及连翘叶抑菌抗炎活性及其主要化学成分研究[J]. 中国药物与临床, 2019, **19**(14): 2380-2381. DOI: 10.11655/zgywylc2019.14.021.
WANG T T, ZHANG H, YANG Z M, *et al*. Antibacterial and Anti-inflammatory Activities of Forsythia Suspense and Forsythia Suspense Leaves and Its Main Chemical Components [J]. *Chin Remedies & Clin*, 2019, **19**(14): 2380-2381. DOI: 10.11655/zgywylc2019.14.021.

[7] 王越, 赵鸿飞, 林创鑫, 等. 连翘苷对LPS刺激的BV2小胶质细胞炎性反应的抑制作用[J]. 中风与神经疾病杂志, 2016, **33**(4): 338-341. DOI: 10.19845/j.cnki.zfysjjbzz.2016.04.013.
WANG Y, ZHAO H F, LIN C X, *et al*. The Inhibitory Effect of Forsythin Inflammation in LPS-induced BV_2 Microglia Cells[J]. *J Apoplexy Nerv Dis*, 2016, **33**(4): 338-341. DOI: 10.19845/j.cnki.zfysjjbzz.2016.04.013.

[8] 陈泽文, 王学方, 李晓, 等. 连翘不同极性部位的抗炎活性研究[J]. 黑龙江畜牧兽医, 2020(18): 124-128. DOI: 10.13881/j.cnki.hljxmsy.2019.11.0088.
CHEN Z W, WANG X F, LI X, *et al*. Study on Anti-inflammatory Activity of Different Polar Parts of *Forsythia suspensa*[J]. *Heilongjiang Animal Sci Vet Med*, 2020(18): 124-128. DOI: 10.13881/j.cnki.hljxmsy. 2019. 11. 0088.

[9] 刘静茹, 姚姗姗, 王文静, 等. 中药黄柏抗炎作用机制的网络药理学分析[J]. 黑龙江畜牧兽医, 2020(20): 117-121. DOI: 10.13881/j.cnki.hljxmsy.2019.11.0250.
LIU J R, YAO S S, WANG W J, *et al*. Network Pharmacology Analysis of Anti-inflammatory Mechanism of *Phellodendron Chinense* Schneid[J]. *Heilongjiang Animal Sci Vet Med*, 2020(20): 117-121. DOI: 10.13881/j.cnki.hljxmsy.2019.11.0250.

[10] 刘莹, 张世超. 基于网络药理学的白花蛇舌草—半枝莲抗肿瘤作用机制研究[J]. 辽宁中医药大学学报, 2020, **22**(8): 27-31. DOI: 10.13194/j.issn.1673-842x.2020.08.008.
LIU Y, ZHANG S C. Study on Anti-tumor Mechanism of *Hedyotis* diffusa and *Scutellaria Barbata* Based on Network Pharmacology[J]. *J Liaoning Univ Tradit Chin Med*, 2020, **22**(8): 27-31. DOI: 10.13194/j.issn. 1673-842x.2020.08.008.

[11] 袁冲, 吴和珍, 刘博, 等. 基于网络药理学的半夏抗炎活性成分及作用机制研究[J]. 中华中医药学刊, 2020, **38**(9): 150-153. DOI: 10.13193/j. issn. 1673-7717. 2020.09.038.
YUAN C, WU H Z, LIU B, *et al*. Anti-inflammatory Active Components and Mechanism of Banxia (*Pinellia* Rhizoma) Based on Network Pharmacology[J]. *Chin Arch Tradit Chin Med*, 2020, **38**(9): 150-153. DOI:

(C)1994-2023 China Academic Journal Electronic Publishing House. All rights reserved. http://www.cnki.net

10.13193/j.issn.1673-7717.2020.09.038.

[12] 雷奇林, 黄雅兰, 钟茜, 等. 基于网络药理学的黄芩抗炎作用机制研究[J]. 中草药, 2018, **49**(15): 3523-3530. DOI: 10.7501/j.issn.0253-2670.2017.15.009.
LEI Q L, HUANG Y L, ZHONG Q, *et al*. Anti-inflammatory Mechanism of Scutellarlae *Radix* Based on Network Pharmacology[J]. *Chin Tradit Herb Drugs*, 2018, **49**(15): 3523-3530. DOI: 10.7501/j. issn. 0253-2670. 2017.15.009.

[13] 但文超, 何庆勇, 曲艺, 等. 基于网络药理学的枳术丸调治血脂异常的分子机制研究[J]. 世界科学技术-中医药现代化, 2019, **21**(11): 2396-2405. DOI: 10.11842/wst.20190723008.
DAN W C, HE Q Y, QU Y, *et al*. Molecular Mechanism of ZHIZHU Pill in Treatment of Dyslipidemia Based on Network Pharmacology[J]. *Mod Tradit Chin Med Mater Med World Sci Technol*, 2019, **21**(11): 2396-2405. DOI: 10.11842/wst.20190723008.

[14] 李聪, 胡强, 张燕翔, 等. 槲皮素的药理学活性研究进展[J]. 湖北中医杂志, 2018, **40**(6): 63-66.
LI C, HU Q, ZHANG Y X, *et al*. Research Progress on Pharmacological Activity of Quercetin [J]. *Hubei J Tradit Chin Med*, 2018, **40**(6): 63-66.

[15] 于倩, 巫冠中. 木犀草素抗炎机制的研究进展[J]. 药学研究, 2019, **38**(2): 108-111. DOI: 10.13506/j.cnki.jpr.2019.02.012.
YU Q, WU G Z. Research Progress of Anti-inflammatory Properties of Luteolin[J]. *J Pharm Res*, 2019, **38**(2): 108-111. DOI: 10.13506/j.cnki.jpr. 2019. 02. 012.

[16] 王伟, 何平, 江小明. 木犀草素及其黄酮苷的抗炎、抗氧化作用[J]. 食品科学, 2020, **41**(17): 208-215. DOI: 10.7506/spkx1002-6630-20190908-103.
WANG W, HE P, JIANG X M. Anti-inflammatory and Antioxidant Effects of Luteolin and Its Flavone Glycosides[J]. *Food Sci*, 2020, **41**(17): 208-215. DOI: 10.7506/spkx1002-6630-20190908-103.

[17] 周霄楠, 韩超, 宋鹏琰, 等. 木犀草素和槲皮素体外抗炎作用研究[J]. 动物医学进展, 2017, **38**(10): 56-61. DOI: 10.16437/j.cnki.1007-5038.2017.10.010.
ZHOU X N, HAN C, SONG P Y, *et al*. Anti-inflammatory Effects of Luteolin and Quercetin in Vitro[J]. *Prog Vet Med*, 2017, **38**(10): 56-61. DOI: 10.16437/j.cnki. 1007-5038.2017.10.010.

[18] 杨本军, 武明飞, 徐涛. 连翘苷调控炎症的抑制作用及机制研究[J]. 安徽医科大学学报, 2020, **55**(7): 1093-1097. DOI: 10.19405/j.cnki.issn1000-1492.2020.07.021.
YANG B J, WU M F, XU T. Effect and Mechanism of Phillyrin on Inhibiting Inflammation[J]. *Acta Univ Med Anhui*, 2020, **55**(7): 1093-1097. DOI: 10.19405/j.cnki. issn1000-1492.2020.07.021.

[19] 李佳行, 杨胜乾, 刘娟娟, 等. 连翘苷对脂多糖诱导肝脏星状细胞活化的抑制作用[J]. 第三军医大学学报, 2020, **42**(4): 342-349. DOI: 10.16016/j. 1000-5404. 201910149.
LI J H, YANG S Q, LIU J J, *et al*. Inhibitory Effect of Phillyrin on Lipopolysaccharide-induced Activation of Rat Hepatic Stellate Cells in Vitro[J]. *J Third Mil Med Univ*, 2020, **42**(4): 342-349. DOI: 10.16016/j. 1000-5404.201910149.

[20] 方明楚, 林振浪. 氯喹通过抑制NF-κB和MAPK信号通路减轻脂多糖诱导的BV2小胶质细胞炎症反应[J]. 中国病理生理杂志, 2020, **36**(7): 1320-1326. DOI: 10.3969/j.issn.1000-4718.2020.07.025.
FANG M C, LIN Z L. Chloroquine Attenuates Lipopolysaccharide-stimulated BV_2 Microglial Ac-tivation by Inhibiting NF- KB and MAPK Signaling Pathways [J]. *Chin J Pathophysiol*, 2020, **36**(7): 1320-1326. DOI: 10.3969/j.issn.1000-4718.2020.07.025.

[21] 陈梦静, 楼烨亮, 陈锋. 基于网络药理学的青蒿抗风湿作用机制研究[J]. 重庆医科大学学报, 2022, **47**(1): 113-120. DOI: 10.13406/j.cnki.cyxb.002694.
CHEN M J, LOU Y L, CHEN F. Research on Anti-rheumatic Mechanism of Artemisiae Annuae Herba Based on Network Pharmacology[J]. *J Chongqing Med Univ*, 2022, **47**(1): 113-120. DOI: 10.13406/j. cnki. cyxb.002694.

[22] 高卓维, 陈广鸿, 符路娣. 芍药苷抑制Src/STAT3信号通路协同替莫唑胺抑制胶质瘤细胞增殖和迁移[J]. 实用医学杂志, 2020, **36**(23): 3199-3205. DOI: 10.3969/j.issn.1006-5725.2020.23.007.
GAO Z W, CHEN G H, FU L D. Paeoniflorin Inhibits Src/STAT3 Signaling Pathway and Temozolomide Inhibits Proliferation and Migration of Primary Human Glioma Cells[J]. *J Pract Med*, 2020, **36**(23): 3199-3205. DOI: 10.3969/j.issn.1006-5725.2020.23.007.

[23] 戈杰, 周培培, 徐天舒, 等. IGF1通过PI3K-AKT通路调控2型糖尿病患者颌骨改建的体外机制研究[J]. 口腔医学研究, 2020, **36**(12): 1157-1161. DOI: 10.13701/j.cnki.kqyxyj.2020.12.017.
GE J, ZHOU P P, XU T S, *et al*. IGF_1 Participation in Bone Regeneration of Type 2 Diabetes Mellitus Patients through PI3K-AKT Signaling Pathway in Vitro [J]. *J Oral Sci Res*, 2020, **36**(12): 1157-1161. DOI: 10.13701/j.cnki.kqyxyj.2020.12.017.

[24] 吴美玉, 阮靖华, 钟伯雄. 人表皮生长因子的研究进展[J]. 生物工程学报, 2020, **36**(12): 2813-2823. DOI: 10.13345/j.cjb.200209.
WU M Y, RUAN J H, ZHONG N. Progress in Human Epidermal Growth Factor Research[J]. *Chin J Biotechnol*, 2020, **36**(12): 2813-2823. DOI: 10.13345/j.

(C)1994-2023 China Academic Journal Electronic Publishing House. All rights reserved. http://www.cnki.net

cjb.200209.

[25] 陈婷婷, 赵嘉琪, 冉明奇. 膳食炎症指数与癌症关系的研究进展[J]. 重庆医学, 2021, **50**(2): 333-337. DOI: 10.3969/j.issn.1671-8348.2021.02.033.
CHEN T T, ZHAO J Q, RAN M Q. Research Progress of Association between Dietary Inflammatory Index and Cancer[J]. *Chongqing Med*, 2021, **50**(2): 333-337. DOI: 10.3969/j.issn.1671-8348.2021.02.033.

[26] 陈楠楠, 戴德. 慢性炎症在恶性肿瘤中的作用研究进展[J]. 中国医学创新, 2020, **17**(14): 169-172. DOI: 10.3969/j.issn.1674-4985.2020.14.043.
CHEN N N, DAI D. Progress in the Role of Chronic Inflammation in Malignant Tumors[J]. *Med Innov China*, 2020, **17**(14): 169-172. DOI: 10.3969/j. issn. 1674-4985.2020.14.043.

[27] BORBA V V, ZANDMAN-GODDARD G, SHOENFELD Y. Prolactin and Autoimmunity: The Hormone as an Inflammatory Cytokine[J]. *Best Pract Res Clin Endocrinol Metab*, 2019, **33**(6): 101324. DOI: 10.1016/j.beem.2019.101324.

[28] ANAGNOSTOU I, REYES-MENDOZA J, MORALES T. Glial Cells as Mediators of Protective Actions of Prolactin (PRL) in the CNS[J]. *Gen Comp Endocrinol*, 2018, **265**: 106-110. DOI: 10.1016/j. ygcen. 2018.01. 024.

[29] YOUSEFVAND S, HADJZADEH M A, VAFAEE F, *et al*. The Protective Effects of Prolactin on Brain Injury [J]. *Life Sci*, 2020, **263**: 118547. DOI: 10.1016/j.lfs.2020.118547.

[30] KAUFMANN S H E, DORHOI A. Inflammation in Tuberculosis: Interactions, Imbalances and Interventions [J]. *Curr Opin Immunol*, 2013, **25**(4): 441-449. DOI: 10.1016/j.coi.2013.05.005.

[31] PIERGALLINI T J, TURNER J. Tuberculosis in the Elderly: Why Inflammation Matters[J]. *Exp Gerontol*, 2018, **105**: 32-39. DOI: 10.1016/j.exger.2017.12.021.

[32] 唐晓林, 赵紫平. 利奈唑胺治疗耐药肺结核对患者血清炎症因子水平的影响[J]. 医学理论与实践, 2020, **33**(23): 3919-3921. DOI: 10.19381/j. issn. 1001-7585.2020. 23.018.
TANG X L, ZHAO Z P. Effect of Linezolid Treatment of Drug-resistant Pulmonary Tuberculosis on the Level of Serum Inflammatory Factors [J]. *J Med Theory Pract*, 2020, **33**(23): 3919-3921. DOI: 10.19381/j.issn.1001-7585.2020.23.018.

[33] 刘朝晖, 马剑雄, 田爱现, 等. 柚皮苷对破骨细胞分化和极化影响的初步探究[J]. 天津医药, 2020, **48**(12): 1141-1145. DOI: 10.11958/20202336.
LIU Z H, MA J X, TIAN A X, *et al*. Effects of Naringin on Differentiation and Polarization of Osteoclasts[J]. *Tianjin Med J*, 2020, **48**(12): 1141-1145. DOI: 10.11958/20202336.

[34] 唐魁韩, 陈龙, 刘平举, 等. 中医药基于核因子κB信号通路调控破骨细胞分化的研究进展[J]. 世界中医药, 2022, **17**(4): 571-578. DOI: 10.3969/j. issn. 1673-7202.2022.04.023.
TANG K H, CHEN L, LIU P J, *et al*. Research Progress of Regulation of Osteoclast Differentiation[J]. *World Chin Med*, 2022, **17**(4): 571-578. DOI: 10.3969/j.issn.1673-7202.2022.04.023.

[35] RABAAN A A, AL-AHMED S H, BAZZI A M, *et al*. Overview of Hepatitis C Infection, Molecular Biology, and New Treatment[J]. *J Infect Public Health*, 2020, **13**(5): 773-783. DOI: 10.1016/j.jiph.2019.11.015.

[36] 刘阳, 冯悦, 翁长江, 等. 丙型肝炎炎症发生与细胞信号通路异常[J]. 生命的化学, 2016, **36**(4): 532-537. DOI: 10.13488/j.smhx.20160419.
LIU Y, FENG Y, WENG C J, *et al*. Inflammation of Hepatitis C and Abnormality of Cell Signaling Pathway [J]. *Chem Life*, 2016, **36**(4): 532-537. DOI: 10.13488/j.smhx.20160419.

[37] LEE H H, CHIN A, PAK K, *et al*. Role of the PI3K/AKT Pathway and PTEN in Otitis Media[J]. *Exp Cell Res*, 2020, **387**(1): 111758. DOI: 10.1016/j. yexcr.2019.111758.

[38] CIANCIULLI A, CALVELLO R, PORRO C, *et al*. PI_3k/Akt Signalling Pathway Plays a Crucial Role in the Anti-inflammatory Effects of Curcumin in LPS-activated Microglia[J]. *Int Immunopharmacol*, 2016, **36**: 282-290. DOI: 10.1016/j.intimp.2016.05.007.[PubMed]

[39] 陈忠仁, 欧宗兴, 王蕾, 等. PI3K信号通路在慢性阻塞性肺疾病大鼠气道及系统性炎症中的作用[J]. 中国老年学杂志, 2020, **40**(23): 5032-5035. DOI: 10.3969/j.issn.1005-9202.2020.23.030.
CHEN Z R, OU Z X, WANG L, *et al*. The Role of PI3K Signaling Pathway in Airway and Systemic Inflammation in Rats with Chronic Obstructive Pulmonary Disease [J]. *Chin J Gerontol*, 2020, **40**(23): 5032-5035. DOI: 10.3969/j.issn.1005-9202.2020.23.030.

[40] 汝触会, 何飞, 郑琦, 等. 基于PI3K/Akt信号通路探讨芍药甘草汤对哮喘大鼠Treg细胞的作用[J]. 中国中医药科技, 2020, **27**(6): 862-865.
RU C H, HE F, ZHENG Q, *et al*. Effects of Shaoyao Gancao Decoction (芍药甘草汤)on Treg Cells in Rats with Asthma Based on PI3K/Akt Signal Path[J]. *Chin J Tradit Med Sci Technol*, 2020, **27**(6): 862-865.

(C)1994-2023 China Academic Journal Electronic Publishing House. All rights reserved. http://www.cnki.net
