# Supplementary material for: Efficacy of Forsythia suspensa (Thunb.) Vahl on mouse and rat models of inflammation-related diseases: a meta-analysis
Source: Front Pharmacol. 2024 Mar 4;15:1288584. doi: 10.3389/fphar.2024.1288584 (PMC10946063; doi:10.3389/fphar.2024.1288584)
Supplement: Supplementary file 1 [file DataSheet1.zip › Data Sheet 1/This meta-analysis included research articles written in Chinese and we packed all these Chinese publications as the supplementary file/Xia et al 2016.pdf]

· 综述 ·

连翘化学成分及其药理学研究进展

夏伟，董诚明*，杨朝帆，陈浩
（河南中医学院，河南 郑州 450008）

［摘要］ 连翘作为市场上常用大宗药材之一，其用量近几年逐年增长，而连翘药材主要以野生为主。笔者综述了连翘的资源分布以及化学成分和药理作用，探讨了连翘的开发前景及药用价值，其目的在于最大化利用连翘资源。

［关键词］ 连翘；资源分布；化学成分；药理作用

Research Progress on Chemical Constituents and Pharmacology of *Forsythia suspensa*

XIA Wei，DONG Chengming*，YANG Chaofan，CHEN Hao
（*Henan University of Traditional Chinese Medicine*，*Zhengzhou Henan*，450008，*China*）

［Abstract］ *Forsythia suspensa* is one of the commonly used Chinese medicinal materials in the market and its annual consumption has increased in recent years，but *F. suspensa* is mainly wild. The author summarizes the resources distribution，chemical composition and pharmacological effects of *F. suspensa* and discusses the future development and medicinal value，in order to maximize the use of *F. suspensa* resources.

［Keywords］ *Forsythia suspensa*；resource distribution；chemical constituents；pharmacological action
doi：10.13313/j.issn.1673-4890.2016.12.031

连翘来源于木犀科植物连翘 *Forsythia suspensa*（Thunb.）Vahl. 的干燥果实，别名黄花条、连壳、青翘、落翘、黄奇丹。连翘作为中药材在我国已有悠久的历史，连翘气微香，味苦。具清热解毒，散结，消肿等功能。传统的应用领域主要集中在解热、抗炎等方面。连翘是市场上用药量较大的中药材之一。为进一步研究连翘，本文对连翘近年来的分布、化学成分、药理作用、有效成分提取方法及质量评价方法进行综述，并对其应用前景进行展望。

1 资源分布

我国连翘资源比较丰富，华北黄土高原是其分布中心，主要分布于河北、山西、陕西、山东、安徽西部、河南、湖北、四川，生于山坡灌丛、林下或草丛中，海拔 250 ~ 2200 m。我国除华南地区外其他各地均有少量栽培，以山西、陕西、河南产量最多[1]。从主产区资源和历史收购情况来看，山西省约占全国的 40%，河南占 30%，陕西占 20% 左右，其余主要分布于湖南、湖北、宁夏、甘肃、河北、山东、辽宁等省[2]。山西连翘广泛分布于中条山、太岳山、太行山、吕梁山、五台山等地，其中中条山、太岳山和太行山为山西野生连翘三大分布区，约占山西连翘总产量的 70%。河南卢氏、栾川、灵宝、嵩县、西峡、南召、济源、辉县等地为连翘主产区；陕西省商洛地区特有的地理位置、气候、土壤条件都很适合连翘的生长，是全国连翘主产区之一；湖北省十堰市郧县地处鄂西之北，是湖北连翘的主产区；河北涉县位于太行山东麓，河北省西南部，晋冀豫三省交界处，西与山西省黎城、平顺县相连，南与河南安阳、林州市相临，亦是全国连翘主产区之一[3]。

2 连翘中主要的化学成分

连翘中含有多种化学成分，通过药理实验研究表明，其中的多种化学成分具有一定的生物学活性。连翘中的不同生物活性成分具有不同的药理作用，各成分相互之间发挥协同作用从而用于不同疾病的治疗。

* ［通信作者］ 董诚明，教授，硕士生导师，研究方向：中药材规范化种植，Tel：（0371）65962581，E-mail：dcm371@sohu.com

(C)1994-2021 China Academic Journal Electronic Publishing House. All rights reserved. http://www.cnki.net

2.1 苯乙醇苷类

苯乙醇及其苷类化合物是连翘的主要特征性有效成分之一。现已从连翘中分离得到连翘酯苷 A、B、C、D、E、F、H、I、J，连翘酚，异连翘酯苷，calceolarioside A，plantainoside A，suspensaside A，suspensaside B，毛柳苷，β-羟基泽丁香酚苷，泽丁香酚苷，木通苯乙醇苷 B，2-(3，4-二羟基苯基)乙基-β-D-吡喃葡萄糖苷等[4-12,15]。

2.2 木脂素类

木脂素类成分是连翘中的另一类有效成分，主要包括连翘苷、(+)-松脂素-β-D-吡喃葡萄糖苷、(+)-表松脂素-β-D-吡喃葡萄糖苷、cedrusin、(+)-1-羟基-松脂素 4″-O-β-D-吡喃葡萄糖苷、(+)-异落叶松脂素 6α-O-β-D-吡喃葡萄糖苷、(-)-橄榄脂素-4′-β-D-吡喃葡萄糖苷、3，4-二羟基-烯丙基苯 4-O-β-D-吡喃木糖基-(1→6)-β-D-吡喃葡萄糖苷、8-羟基松脂素、连翘脂素、异橄榄脂素、forsythialan A、forsythialan B[6-7,11,13-14]。

2.3 萜类

萜类包括五福花苷酸、齐墩果酸、熊果酸、2α，23-羟基熊果酸、苯甲醇樱草糖苷、苯甲醇 O-(2′-O-β-D-吡喃木糖苷)-β-D-吡喃葡萄糖苷、乙酰齐墩果酸、异降香萜烯醇乙酸酯、β-香树脂醇乙酸酯、积雪草酸、商陆种酸、甲基-β-D-吡喃葡萄糖、长管大青素 A、20(S)-达玛烷-24-烯-3β，20-二醇-3-乙酸酯、20S，24S-环氧达玛烷-25-醇-3α-羟基乙酸酯、白桦酯酸等[6-7,13,15-18]。

2.4 黄酮类

有机酸包括槲皮素、异槲皮素、木犀草苷、山柰酚、异鼠李素、芦丁、橙皮苷、木犀草素、异鼠李素-3-O-α-L-吡喃鼠李糖苷-(1→2)-β-D-吡喃葡萄糖苷、翻白叶苷 A、金丝桃苷等[7,11,13,19]。

2.5 有机酸

有机酸包括咖啡酸、阿魏酸、没食子酸、对羟基苯乙酸、3，4-二羟基苯甲酸、硬脂酸、棕榈酸、香荚兰酸、琥珀酸、丁二酸等[6-18,20]。

2.6 其他类

甾体类化合物包括胡萝卜苷、β-谷甾醇、18-去甲基 5α，20ε-去氧胆酸等[16,18,20]。

其他类包括 3β，20-二羟基-4，4，8，14-四甲基-γ-内酯乙酸酯等；生物碱八氢-1H，5H-二吡咯 [1，2-a：1′.2′-d] 吡嗪，(-)-egenine 等。

3 药理作用

连翘一直是市售常用药材之一，在近几年连翘药理学研究中，发现其主要有抗菌、解热、抗炎、抗病毒、抗内毒素、保肝、抗肿瘤等作用。

3.1 抗菌作用

连翘为广谱抗菌药物，对多种细菌均有一定的抑制作用。秦臻[21]在对连翘研究中得出，连翘叶具有较强的抑菌作用。在对有效抑菌成分分离中得出，青翘 40% 乙醇提取物的 30% 洗脱物，其抑菌效果最好，且得出抑菌的有效成分为连翘酯苷 A。王婷婷[22]在对黄连、连翘协同抑菌的研究中得出，连翘及其药对的乙醇提取物对大肠杆菌金黄色葡萄球菌、枯草芽孢杆菌、藤黄微球菌等 7 种供试菌均表现出一定的抑制作用。权志博[23]将中药制成水煎剂原药，用琼脂二倍稀释法进行体外抑菌效果观察。结果得出，连翘单味水煎剂对产 ESBLs(超广谱β-内酰胺酶)大肠埃希菌 20 株临床株均有不同程度的抑菌作用。王新等[24]在对甲氧苄啶对连翘体外抗菌增效作用的研究中发现，连翘与甲氧苄啶联用对金黄色葡萄球菌和大肠杆菌抑制作用增强，呈协同作用。连翘挥发油有广谱抗菌作用[25]。连翘酚具有较强的抗菌活性[3]。连翘水煎液对金黄色葡萄球菌等 G^+ 菌抗菌作用强，对伤寒杆菌、奇异变形杆菌等 G^- 菌抗菌作用弱。因此连翘主要用于上呼吸道感染、急性肾炎，而不用于肠道革兰阴性菌引起的感染[26]。(+)-松脂素-β-D-葡萄糖苷和 forsythiaside 均有一定的抗菌活性，且 forsythiaside 较(+)-松脂素-β-D-葡萄糖苷的作用强[27]。

3.2 抗病毒作用

近年来，国内外有关抗病毒中药的研究日益增多，而常用药材连翘无疑成为近年来研究的重点。

陈杨等[28]在连翘抗病毒有效部位(LC-4)体外抗呼吸道合胞病毒(RSV)作用的研究中，得出 LC-4 在体外对 RSV 复制有明显的预防作用及治疗作用。杨溢[29]以水煎法提取连翘的有效成分，以利巴韦林为阳性对照药物，用细胞病变(CPE)观察法、噻唑蓝盐微量酶反应比色法(MTT)检测细胞活性，对连翘抗猪流感病毒的效果进行评价。实验得出，其具有抗病毒作用，主要是通过抵抗病毒吸附、干扰病毒复制和直接杀灭作用来对病毒产生抗性。刘晓静[30]

(C)1994-2021 China Academic Journal Electronic Publishing House. All rights reserved. http://www.cnki.net

在对 3 种中草药抑制甲 3 型流感病毒诱导细胞凋亡的研究中得出，连翘能抑制甲 3 型流感病毒诱导狗肾传代细胞凋亡。张丹丹等[31]研究连翘及其主要有效成分槲皮素体外抗人巨细胞病毒作用和细胞毒性实验中，采用细胞病变法和 MTT 法，检测连翘、槲皮素抗人巨细胞病毒的最大无毒浓度、最小有效浓度和治疗指数，并与更昔洛韦进行比较，最终得出结论：槲皮素抗人巨细胞病毒（HCMV）效果大大高于更昔洛韦和连翘，细胞毒性与连翘相同，比更昔洛韦低。连翘尤其是槲皮素具有良好的体外抗人巨细胞病毒效果。连翘酯苷合胞病毒、腺病毒 s 型和 7 型、柯萨奇病毒 B 组 3 型和 7 型均有一定的抑制作用。其中，对合胞病毒、柯萨奇病毒 3 亚型和副流感病毒 3 型无体外抑制作用[32]。另有报道，连翘有效成分体外对单纯疱疹病毒具有抑制作用[33]。连翘抗流感病毒的主要有效部位为酚类成分[2]。

3.3 抗炎作用

厉世伟[34]利用 0.22 μm 滤膜对培养过大肠杆菌的 LB 培养液进行除菌，所得滤过液作为致炎物，建立上皮细胞的炎症模型。将连翘水煎液和连翘苷分别作用于细胞炎症模型，通过细胞形态观察以及利用 MTT 法比色计数来判断细胞的活性。实验表明，0.002 5 $g \cdot L^{-1}$连翘黄酮类成分均致细胞死亡，上皮细胞均裂解破碎。实验中连翘水煎液与连翘苷的有效作用浓度一样，均在 0.25 ~ 0.13 $g \cdot L^{-1}$时对细胞有显著的保护作用，且发现连翘水煎液质量浓度为 0.13 $g \cdot L^{-1}$时或许具有促正常细胞增殖的作用。在研究药物对炎症细胞的保护作用中发现，连翘水煎液优于连翘苷。郭际[35]用二甲苯（0.03 mL/只）涂于小鼠右耳两面致炎，左耳不处理，将对照组与给药组致炎 1 h 后处死，用打孔器打下双耳相同部位，测其肿胀度。结果表明，与模型对照组比较，连翘挥发油高剂量组、低剂量组（0.24、0.12 $mL \cdot kg^{-1}$）有明显的抑制二甲苯所致小鼠的耳廓肿胀作用。倪力军等[36]在对二甲苯所致小鼠耳壳炎症模型的抗炎实验中，通过测定致炎小鼠耳肿胀度得出，连翘水煎液具有抗炎作用。胡竟一等[37]对连翘果壳与种子分别制为不同提取物，并对二甲苯或巴豆油所致小鼠耳肿胀进行研究，得到连翘果壳水煎剂、大孔树脂吸附物及连翘多酚为连翘抗炎作用的主要有效部位。另有报道，连翘酯苷具有一定的抗炎作用[38]。连翘苷同样有抗炎作用[39]。芮菁等[40]将连翘干燥果实的甲醇提取液、正己烷萃取甲醇提取液得到的正己烷可溶物和水可溶物分别进行真空冷冻干燥。得到 3 种棕色粉末状物质。小鼠毛细血管通透性、扭体反应、足趾肿胀和肉芽肿实验表明，连翘甲醇提取物和正己烷可溶物具有消炎和镇痛作用，而水溶物则无上述效果。

3.4 连翘保肝作用

杨建雄等[41]采用给小鼠腹腔注射四氯化碳制作肝脏损伤模型，通过测定小鼠血清中丙氨酸氨基转移酶（ALT）、天冬氨酸氨基转移酶（AST）、胆碱脂酶（CHE）、总胆红素（TBIL）、总蛋白（TP）、谷胱甘肽（GSH）和肝脏中超氧化歧化酶（SOD）、丙二醛（MDA）、CHE、白蛋白（ALB）、ALT、GSH 等指标，研究连翘叶茶对四氯化碳所致小鼠急性肝脏损伤的保护作用。结果表明，连翘叶茶可抑制血清和肝脏中 ALT 和 AST 的异常增高，提高血清和肝脏中 SOD 和 CHE 的活性，增加 GSH、TP 和钴结合蛋白（ACB）含量，减少 MDA 形成，调节 TBIL 代谢。说明连翘叶茶具有明显的保护肝脏的作用。研究发现，连翘苷具有很好的清除羟基自由基、超氧自由基等活性，抑制过氧化氢诱导的红细胞溶血，可降低体内过氧化产物丙二醛的积累，抑制线粒体的氧化损伤，抗肝损伤，具有保肝作用[42]。连翘苷元亦有保肝作用[43]。

3.5 其他药理作用

综合多年研究，连翘乙醇提取物[44]、连翘苷类成分Ⅰ和Ⅱ[45-47]、连翘叶水提物均具有抗衰老作用[48]；连翘酯苷具有改善拟阿尔茨海默病动物模型学习记忆障碍的作用[49]，并且连翘酯苷在一定程度上可防护顺铂所致的耳廓损伤[50]；近年来，连翘在肿瘤方面的应用也得到了发展，连翘脂素和表松脂素对人胃癌细胞株 SGC7901 生长具有一定的抑制作用[16]。连翘作为常用药材，在调节免疫、抗应激、解热镇痛等方面的研究报道也较多[51]。

4　开发利用及展望

连翘历来被称作“疮家圣药”，并且在清热解毒方面也极具疗效。主要应用方剂有双黄连口服液、VC 银翘片、双黄连粉针剂、银翘解毒合剂、连花清瘟胶囊、银翘解毒丸等。据近几年市场走势，连翘用量日益增长，并且市场价格自 2013 年涨幅较大，拉动了产区经济增长。但由于市场流通连翘主要来

(C)1994-2021 China Academic Journal Electronic Publishing House. All rights reserved.　http://www.cnki.net

源为野生连翘，产区连翘产量受环境因素影响较大。国家对中药材的规范化种植的大力扶持，推动了中药材种植的热情，山西、陕西、河南和河北这几大连翘主产区也相继建立了大大小小的连翘规范化种植基地，为连翘可持续发展作出了贡献。

我国盛产连翘，在当今对环境的关注下，连翘的开发利用前景十分广阔。连翘不仅是传统的药用树种，也是重要的经济树种，又是重要的油料作物、观赏植物和水土保持植物。另外，连翘也可做食品天然防腐剂或化妆品，应用广泛，市场前景广阔。连翘花在初春开放，花期长，具有很高的观赏性，亦可在旅游行业中进行开发。进一步研究连翘是一项非常有意义的工作。

参考文献

[1] 梁焕忠. 野生连翘资源保护与可持续利用研究[J]. 科学之友，2010(4)：156-157.

[2] 胡静，马琳，张坚，等. 连翘的研究进展[J]. 中南药学，2012，10(10)：760-764.

[3] 郭丁丁，张潞，朱秀峰. 中药连翘种质资源调查报告[J]. 时珍国医国药，2012，23(10)：2601-2603.

[4] Liu D L，Zhang Y，Xu S X，et al. Phenylethanoid glycosides from *Forsythia suspensa* Vahl.[J]. J Chin Pharm Sci，1998，7(2)：103-105.

[5] 原江锋，邱智军，刘建利，等. 河南和山西连翘叶中总木脂素、连翘醋苷 A、连翘醋苷 B 和连翘苷的含量比较[J]. 天然产物研究与开发，2015，27(5)：845-848.

[6] 赵文华. 连翘抗流感病毒有效物质基础研究黄连解毒汤有效部位的藏红花酸分析方法研究[D]. 北京：北京中医药大学，2003.

[7] 王福男. 中药连翘的化学成分研究[D]. 北京：中国协和医科大学，2009.

[8] 田燕泽. 中药连翘化学成分分离及其抗氧化活性评价[D]. 北京：中央民族大学，2011.

[9] 俞崇灵. 连翘抗菌成份的研究[J]. 药学学报，1960，8(6)：241-244.

[10] 曲欢欢. 连翘化学成分和生物活性研究[D]. 西安：西北大学，2008.

[11] 田燕泽. 中药连翘化学成分分离及其抗氧化活性评价[D]. 北京：中央民族大学，2011.

[12] 秦宇，张文丽，林媛媛，等. 连翘化学成分与抗氧化活性成分研究[J]. 中国实验方剂学杂志，2013，5，19(10)：149-152.

[13] 邹琼宇，邓文龙，丁立生，等. 连翘果实中的化学成分研究[J]. 中国中药杂志，2012，37(1)：57-60.

[14] Piao X L，Jang M H，Cui J，et al. Lignans from the fruits of *Forsythia suspense*[J]. Bioorg Med Chen Lett，2008，18(6)：1980.

[15] 侯雅楠. 青翘化学成分提取分离及初步活性研究[D]. 太原：山西大学，2013.

[16] 毛威. 连翘化学成分及其抗肿瘤活性研究[D]. 武汉：湖北中医学院，2009.

[17] 吴艳芳，王新胜，袁永亮，等. 连翘化学成分研究[J]. 中草药，2013，44(15)：2052-2054.

[18] 冯雪. 连翘籽化学成分研究[D]. 沈阳：辽宁中医药大学，2008.

[19] 原江锋，赵君峰，孙军杰，等. 河南、山西连翘叶黄酮类和三萜酸类化合物含量比较[J]. 良晶科学，2015，36(10)：164-167.

[20] Dai S J，Ren Y，Shen L，etal. Newslkaloids from *Forsythia suspensa* and their ani-inflanmatory activities[J]. Planta Med，2009，75(4)：375-377.

[21] 秦臻. 利用连翘叶制备天然防腐剂的初步研究[D]. 太原：山西大学，2013.

[22] 王婷婷. 黄连、连翘协同抑菌配方优化及保鲜应用研究[D]. 泰安：山东农业大学，2011.

[23] 权志博，周雪宁，王雷. 不同药性清热中药体外抗产 ESBLs 大肠埃希菌效果及分析[J]. 当代医学，2009，15(25)：149-150.

[24] 王新，崔一喆，邵红. 甲氧苄啶对连翘体外抗菌增效作用的研究[J]. 黑龙江八一农垦大学学报，2011，23(4)：30-32.

[25] 肖会敏. 连翘挥发油提取工艺及其初步活性的研究[D]. 西安：第四军医大学，2006.

[26] 李晓燕. 中药连翘抗菌活性的考察[J]. 山东医药工业，1997，16(2)：46-47.

[27] 匡海学，张宁，陆志博. 青连翘杭菌活性成分的研究[J]. 中药通报，1988，13(7)：32-34.

[28] 陈杨，李鑫，周婧瑜，等. 连翘抗病毒有效部位(LC-4)体外抗呼吸道合胞病毒(RSV)作用的研究[J]. 卫生研究，2009，38(6)：733-735.

[29] 杨溢. 抗猪流感病毒单味中药的体外筛选[D]. 泰安：山东农业大学，2013.

[30] 刘晓静. 三种中草药抑制甲 3 型流感病毒诱导细胞凋亡的研究[D]. 南昌：南昌大学，2007.

[31] 张丹丹，方建国，陈素华，等. 连翘及其主要有效成分槲皮素体外抗人巨细胞病毒的实验研究[J]. 中国中药杂志，2010，35(8)：1055-1059.

[32] 胡克杰，徐凯建，王跃红，等. 连翘酯苷体外抗病毒作用的实验研究[J]. 中国中医药科技，2001，8(2)：89.

[33] 刘颖娟，杨占秋，肖红，等. 中药连翘有效成分体外抗单纯疱疹病毒的实验研究[J]. 湖北中医学院学报，2004，6

(C)1994-2021 China Academic Journal Electronic Publishing House. All rights reserved.　http://www.cnki.net

(1):36-38.
[34] 厉世伟. 金银花、连翘抑制奶牛乳腺上皮细胞炎症反应的作用研究[D]. 乌鲁木齐: 新疆农业大学,2013.
[35] 郭际. 连翘挥发油抗炎作用及机理研究[D]. 成都: 成都中医药大学,2005.
[36] 倪力军,张立国,史万忠,等. 解热抗炎中药挥发性成分的药效学研究[J]. 中草药,2007,29(8):1217-1221.
[37] 胡竟一,雷玲,余悦,等. 连翘的抗炎解热作用研究[J]. 中药药理与临床,2007,23(3):51-52.
[38] 李鹤. 连翘酯苷对鸡肝脏抗氧化及抗炎活性研究[D]. 哈尔滨: 东北农业大学,2013.
[39] 潘晓龙. 连翘苷对 LPS 诱导炎症反应的影响及分子机制的研究[D]. 南京: 南京师范大学,2014.
[40] 芮菁,尾崎幸纮,唐元泰. 连翘提取物的抗炎镇痛作用[J]. 中草药,1999,30(1):43-45.
[41] 杨建雄,刘静. 连翘叶茶保肝作用的实验研究[J]. 陕西师范大学学报(自然科学版),2005,33(3):82-85.
[42] 张杲. 连翘叶在河北、陕西和河南常用作保健茶[D]. 西安: 陕西师范大学,2006.
[43] 冯芹,夏文凯,王现珍,等. 连翘苷元对四氯化碳大鼠急性肝损伤的保护作用[J]. 中国药理学通报,2015,31(3):426-430.
[44] 涂秋云,周春山,杨坚萍,等. 连翘乙醇提取物清除脂自由基和氧自由基的效果[J]. 湖南农业大学学报(自然科学版),2008,34(6):728-731.
[45] 杨建雄,杨晨,邱娟,等. 连翘叶中苷类成分Ⅰ的体外抗氧化作用研究[J]. 陕西师范大学(自然科学版),2006,34(4):79-82.
[46] 邱娟,杨晨,杨建雄,等. 连翘叶中苷类成分Ⅱ的体外抗氧化作用研究[J]. 陕西理工学院学报,2007,23(2):53-57.
[47] 魏希颖,徐慧娴,扬小军,等. 连翘种子油 GC-MS 分析及抗氧化活性研究[J]. 陕西师范大学学报(自然科学版),2010,38(1):70-74.
[48] 李兴泰,陈瑞,高明波. 连翘叶水提物保护线粒体及抗衰老研究[J]. 食品与生物技术学报,2009,28(6):840-844.
[49] 李长禄,王红梅,王立为. 连翘酯苷对拟 AD 动物模型学习记忆的改善作用[J]. 山东医药,2012,52(44):4-7.
[50] 黄世勇,陶泽璋,肖伯奎. 连翘酯苷对顺铂耳毒性防护作用的实验研究[J]. 听力学及言语疾病杂志,2011,19(2):152-156.
[51] 刘静. 连翘叶茶抗氧化抗衰老及保肝作用的实验研究[D]. 西安: 陕西师范大学,2004.

(收稿日期 2015-12-24)

(上接第 1669 页)

[20] 张海霞,卢永昌,王庶. GC-MS 分析宽筋藤中不同萃取物成分比较[J]. 生物技术世界,2014(4):62.
[21] 任艳丽. 中华青牛胆的化学成分研究[J]. 天然产物研究与开发,2008,20(2):278-279.
[22] 张敏,李苑,李海棠. 宽筋藤中 L—1,2,4/3,5 环己五醇的分离鉴定[J]. 中药材,1993,16(12):250.
[23] 帝玛尔·丹增彭措.《晶珠本草》[M]. 毛继祖,译. 上海: 上海科学技术出版社,2012.
[24] 滕红丽,梅之南,郭力城. 壮医风湿免疫病常用藤本药物药学及临床应用研究[J]. 辽宁中医杂志,2011,38(5):873-875.
[25] 林耐球,朱英,周广军,等. 壮医药治疗痹证研究概况[J]. 辽宁中医药大学学报,2013,15(11):139-142.
[26] 薛强,罗莹,许小林. 宽筋藤提取物对小鼠抗炎作用的实验研究[J]. 右江医学,2014,42(3):285-287.
[27] 段伟,毕良文,李文辉. 宽筋藤对辐射损伤小鼠造血功能的影响[J]. 中国辐射卫生,2008,6(17):138-140.
[28] Singh N, Kumar A, Gupta P, et al. *Evaluation of antileishmanial potential of Tinospora sinensis* against experimental visceral leishmaniasis[J]. Parasitol Res, 2008,102(3):561-565.
[29] Jain S, Sherlekar B, Barik R. Evaluation of antioxidant potential of *Tinospora cordfolia* and *Tinospora sinensis*[J]. Int J Pharm Sci Res,2010,11(1):122-128.
[30] Manjrekar P N, Jolly C I, Narayanan S. Comparative studies of the immunomodulatory activity of *Tinospora cordifolia* and *Tinospora sinensis*[J]. Fitoterapia,2000,71(3):254-257.

(收稿日期 2016-01-13)

(C)1994-2021 China Academic Journal Electronic Publishing House. All rights reserved. http://www.cnki.net
