# Supplementary material for: Efficacy of Forsythia suspensa (Thunb.) Vahl on mouse and rat models of inflammation-related diseases: a meta-analysis
Source: Front Pharmacol. 2024 Mar 4;15:1288584. doi: 10.3389/fphar.2024.1288584 (PMC10946063; doi:10.3389/fphar.2024.1288584)
Supplement: Supplementary file 1 [file DataSheet1.zip › Data Sheet 1/This meta-analysis included research articles written in Chinese and we packed all these Chinese publications as the supplementary file/Zhang et al 2022.pdf]

· 论著 ·

连翘提取物对慢性盆腔炎模型大鼠的干预作用及对ICAM-1和INF-r水平的影响*

张永会　罗福兰　胡红娟

（云南省中医医院妇科，云南 昆明 650021）

【摘要】 目的　探讨连翘提取物对慢性盆腔炎模型大鼠的干预作用及对细胞间黏附分子-1(ICAM-1)、γ-干扰素(INF-γ)水平的影响。方法　选取SPF级40只SD健康大鼠，按照双盲法随机分为正常组、模型组、阳性对照组、连翘提取物组，每组10只。模型组、阳性对照组、连翘提取物组建立慢性盆腔炎大鼠模型，建模型成功后，阳性对照组给予剂量为0.065 g/kg的氟哌酸胶囊剂灌胃处理，连翘提取物组使用连翘提取物250 mg/kg进行灌胃给药，正常组和模型组不作任何处理。对4组大鼠免疫球蛋白含量、ICAM-1、INF-γ、TLR4、NF-kB水平、子宫系数、红细胞压积、血浆粘度、右侧子宫质量及肿胀度进行检测。结果　连翘提取物组大鼠血浆免疫球蛋白含量、INF-γ水平均高于阳性对照组，且ICAM-1、TLR4、NF-kB、子宫系数、红细胞压积、血浆粘度、子宫质量、肿胀度均低于阳性对照组，差异有统计学意义（均$P<0.05$）。结论　连翘提取物能够有效缓解大鼠子宫黏连，改善体内炎症反应及血流病变异常，其机制可能与子宫组织ICAM-1、INF-γ表达有关。

【关键词】 慢性盆腔炎；连翘提取物；细胞间黏附分子-1；γ-干扰素

【中图分类号】 R711.33　【文献标志码】 A　DOI:10.3969/j.issn.1672-3511.2022.02.007

Effect of Forsythia suspensa extract on the level of ICAM-1 and INF-γ in chronic pelvic in flammatory model rats

ZHANG Yonghui, LUO Fulan, HU Hongjuan

(*Department of Gynaecology, Yunnan Provincial Hospital of Traditional Chinese Medicine, Kunming* 650021, *China*)

【Abstract】 Objective　To study the effect of Forsythia suspensa extract on the level of ICAM-1 (intercellular adhesion molecule-1) and inf -γ(interferon gamma) in chronic pelvic inflammatory model rats. **Methods**　40 SD healthy rats of SPF grade were randomly divided into normal group, model group, positive control group and Forsythia Extract group according to double blind. The model group, positive control group and Forsythia Extract group were used to establish the chronic pelvic inflammatory rat model. After the model was established successfully, the positive control group was given 0.065g/kg norfloxacin capsule, and the Forsythia Extract group was given 250mg / kg The normal group and the model group were not treated. The contents of immunoglobulin, ICAM-1, inf-γ, TLR4, NF-κB, uterine coefficient, hematocrit, plasma viscosity, right uterine mass and swelling degree were measured. **Results**　The content of plasma immunoglobulin and inf-γ water in Forsythia suspense extract group were higher than those in the positive control group, and ICAM-1, TLR4, NF KB, uterine coefficient, hematocrit, plasma viscosity, uterine quality and swelling degree were lower than those in the positive control group ($P<0.05$). **Conclusion**　Forsythia suspensa extract can effectively improve uterine adhesiveness, improve the inflammatory response and abnormal blood flow in rats. Its mechanism may be related to the expression of ICAM-1 and inf-γ in uterine tissue.

【Key words】 Chronic pelvic inflammatory disease; Forsythia suspensa extract; Intercellular adhesion molecule-1; Interferon gamma

引用本文：张永会，罗福兰，胡红娟．连翘提取物对慢性盆腔炎模型大鼠的干预作用及对ICAM-1和INF-r水平的影响[J]．西部医学，2022，34(2)：190-194．DOI：10.3969/j.issn.1672-3511.2022.02.007

(C)1994-2022 China Academic Journal Electronic Publishing House. All rights reserved.　http://www.cnki.net

盆腔炎是指女性生殖道及其周围组织的炎症，慢性盆腔炎是急性期时未得到有效的治疗和诊断，由于患者体质较差而导致形成的。慢性盆腔炎的病情较顽固，有研究表明[1]，此病的发生是局部调节与促炎因子相互作用和共同参与的结果。慢性盆腔炎是一种常见的妇科炎症，且呈现出迁延不愈的状态，患者主要表现为盆腔、腰骶部位疼痛，当身体劳累时，会直接导致病情加重[2]。其病症反复发作，严重影响患者的身心健康，是有待解决的重大妇科难题。连翘是木犀科植物连翘的果实，属于我国常用的 40 味药材之一。其具有清热解毒、消肿散结等功效。有研究表明[3]，连翘具有抗炎、解热、镇痛等药理作用。连翘主要用于急性传染、感染性炎性疾病等治疗中。但目前临床上连翘提取物对慢性盆腔炎作用的报道鲜少，因此本研究中探讨其对慢性盆腔炎小鼠的干预作用，为临床上此病的治疗提供新方向。

1 材料与方法

1.1 一般材料

1.1.1 实验动物 选取 SPF 级 SD 健康大鼠 40 只，由基尔顿生物科技（上海）有限公司提供，鼠龄（3.6±0.2）个月，体重（219.7±8.5）g。所有大鼠均养殖在干净笼子里，室温在（22.1±1.8）℃，相对湿度 35%～40%，每天光照 12 h，喂饮纯净水，饲养时间一周。本文研究所做实验均获得我院伦理委员会批准。

1.1.2 主要试剂 连翘提取物（山东唐正生物科技有限公司）；氟哌酸胶囊剂（广东恒健制药有限公司）。

1.2 方法

1.2.1 分组及建模 双盲随机选取 40 只大鼠中 10 只作为正常组，其余 30 只小鼠建立慢性盆腔炎模型，正常组在建模过程中不做任何处理。参照王新斌等[4]研究实验中慢性盆腔炎大鼠模型建立方法在无菌环境下建立慢性盆腔炎模型，将大鼠使用 100 g/L 的水合氯醛进行麻醉，将其仰卧固定，下腹手术部位进行去毛，常规消毒下，在腹中线于尿道口上方 1 cm 处作 0.8～1.0 cm 切口，将子宫完全暴露，在距离卵巢 2 cm 处，使用 5 号针头将子宫划伤，使用相同力道将子宫划伤 5 次后，分别向右侧输卵管-卵巢方向注入 250 g/L 苯酚胶浆溶液 0.05 mL，分层关腹。建模成功 27 只（成功标准：大鼠子宫内膜组织进行 HE 染色，见腺体增生、大量炎性因子浸润、纤维结缔组织增生，表明建模成功），随机分为模型组、阳性对照组和连翘提取物组各 9 只。

1.2.2 给药 阳性对照组大鼠使用 2 mg/kg 的地塞米松灌胃处理[4]；连翘提取物组使用连翘提取物 250 mg/kg 进行灌胃给药，正常组和模型组不作任何处理。

1.2.3 切片及染色 随机选取各组大鼠行全麻处理后，采用断头法处死，在无菌、低温条件下取小鼠子宫内膜组织，将小鼠子宫内膜组织在 4% 多聚甲醛中浸泡、固定，在温度为 4℃ 的环境下进行保存。之后行 HE 染色处理，观察病理组织变化。

1.2.4 样本采集 抽取 4 组大鼠颈总动脉血 3 mL，在抗凝管中保存，用 3000 r/min 的离心机离心，10 min后，将离心静脉血滴入 EP 试管中，分离上层血清，在-80℃环境下保存，待用。

1.2.5 免疫球蛋白含量检测 采用酶联免疫吸附法对（免疫球蛋白 G）IgG、（免疫球蛋白 A）IgA、（免疫球蛋白 M）IgM 水平进行检测。

1.2.6 ICAM-1、INF-γ、TLR4、NF-kB 水平检测 使用免疫组化法对（细胞间黏附分子-1）ICAM-1 水平进行检测；使用采用酶联免疫双抗体夹心法（ELISA）对（γ-干扰素）INF-γ 水平进行检测；使用（上海纪宁生物科技有限公司）ELISA 试剂盒对（Toll 样受体 4）TLR4、（核转录因子-KB）NF-kB 水平进行检测。

1.2.7 子宫系数、红细胞压积及血浆粘度检测 取大鼠全部子宫，剪去双侧卵巢，剔除脂肪，用电子天平进行称重，计算各组大鼠子宫重量系数，子宫系数（mg/g）＝子宫重（mg）/体重（g）；取 1 mL 全血，放入温室压积管中，3000 r/min 进行离心 20 min 后读取红细胞压积值；将全血、血浆注入血流变仪，然后检测血浆粘度。

1.2.8 右侧子宫质量、左侧子宫质量及肿胀度检测 子宫肿胀度（g）＝右侧子宫质量-左侧子宫质量。

1.3 统计学分析

采用 SPSS 19.0 统计软件包进行统计分析处理。计量资料采用均数±标准差（$\bar{x}\pm s$）描述，多组间比较采用 F 值检验，两组间比较采用独立样本 t 检验，$P<0.05$ 为差异有统计学意义。

2 结果

2.1 4 组大鼠病理组织学观察 正常组大鼠子宫内膜组织正常，子宫壁界限清晰，未见明显炎性细胞浸润；模型组大鼠浆膜层可发现大量炎性细胞，肌层血管明显水肿，结蹄组织明显增生，子宫腔内出现不同程度黏连；阳性对照组大鼠子宫组织结构恢复较好，但仍有炎性介质的参与；连翘提取物组大鼠子宫组织结构基本恢复正常，炎性细胞浸润和组织增生变少。见图 1。

2.2 4 组大鼠血浆免疫球蛋白含量比较 模型组、阳性对照组、连翘提取物组大鼠血浆免疫球蛋白含量均低于正常组，差异有统计学意义（$P<0.05$）；阳性对照组、连翘提取物组大鼠血浆免疫球蛋白含量均高于模型组，差异有统计学意义（$P<0.05$）；连翘提取物组大

(C)1994-2022 China Academic Journal Electronic Publishing House. All rights reserved. http://www.cnki.net

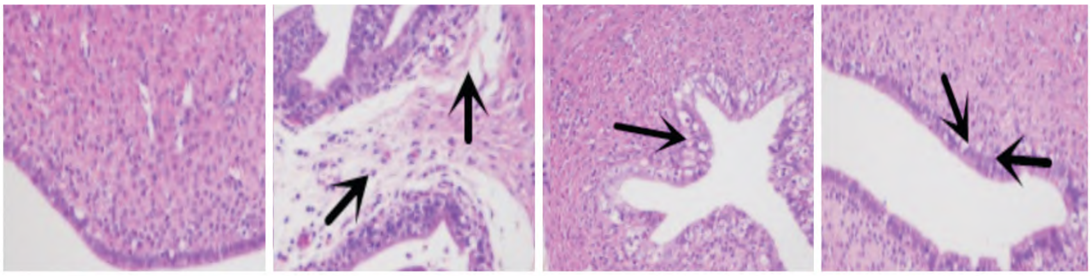

正常组　　模型组　　阳性对照组　　连翘提取物组

图 1　4 组小鼠子宫组织病理组织学观察（HE 200×）

Figure 1　Histopathological observation of uterus in four groups of mice

鼠血浆免疫球蛋白含量均高于阳性对照组，差异有统计学意义（$P<0.05$）。见表 1。

表 1　4 组大鼠血浆免疫球蛋白含量比较[（$\bar{x}\pm s$），μg/mL]

Table 1　Comparison of plasma immunoglobulin contents in four groups of rats

组别	n	IgG	IgA	IgM
正常组	10	64.15±3.66	53.26±6.66	15.22±1.66
模型组	9	27.15±5.11①	21.22±1.59①	4.25±1.01①
阳性对照组	9	33.25±4.56①②	31.56±4.88①②	9.56±1.17①②
连翘提取物组	9	51.22±5.21①②③	45.66±5.98①②③	13.88±1.59①②③
F		27.439	21.058	25.720
P		<0.05	<0.05	<0.05

注：与正常对照组相比，①$P<0.05$；与模型组相比，②$P<0.05$；与阳性对照组相比，③$P<0.05$

2.3　4 组大鼠体内 ICAM-1、INF-γ、TLR4、NF-kB 水平比较　**模型组、阳性对照组、连翘提取物组大鼠体内 ICAM-1、TLR4、NF-kB 均高于正常组，INF-γ 水平低于正常组，差异均有统计学意义（均 $P<0.05$）；阳性对照组、连翘提取物组大鼠体内 ICAM-1、TLR4、NF-kB 均低于模型组，INF-γ 水平高于模型组，差异有统计学意义（均 $P<0.05$）；连翘提取物组大鼠体内 ICAM-1、TLR4、NF-kB 均低于阳性对照组，INF-γ 水平高于阳性对照组，差异均有统计学意义（均 $P<0.05$）。见表 2。**

表 2　4 组大鼠体内 ICAM-1、INF-γ、TLR4、NF-kB 水平比较（$\bar{x}\pm s$）

Table 2　Comparison of ICAM-1，inf－γ，TLR4 and NF KB levels in four groups of rats

组别	n	ICAM-1（$\times10^{-2}$）	INF-γ（pg/mL）	TLR4（ng/mL）	NF-kB（pg/mL）
正常组	10	21.99±3.15	204.56±12.33	0.83±0.05	175.16±19.89
模型组	9	39.89±3.56①	175.33±11.25①	3.29±0.26①	451.22±45.23①
阳性对照组	9	31.88±3.45①②	189.88±9.89①②	2.89±0.15①②	315.33±21.56①②
连翘提取物组	9	23.15±2.15①②③	198.56±10.11①②③	1.61±0.06①②③	215.88±15.66①②③
F		17.448	8.064	44.119	26.324
P		<0.05	<0.05	<0.05	<0.05

注：与正常对照组相比，①$P<0.05$；与模型组相比，②$P<0.05$；与阳性对照组相比，③$P<0.05$

2.4　4 组大鼠子宫系数、红细胞压积、血浆粘度比较　**模型组、阳性对照组、连翘提取物组大鼠子宫系数、红细胞压积、血浆粘度高于正常组，差异有统计学意义（均 $P<0.05$）；阳性对照组、连翘提取物组大鼠子宫系数、红细胞压积、血浆粘度低于模型组，差异均有统计学意义（均 $P<0.05$）；连翘提取物组大鼠子宫系数、红细胞压积、血浆粘度低于阳性对照组，差异有统计学意义（均 $P<0.05$）。见表 3。**

表 3　4 组大鼠子宫系数、红细胞压积、血浆粘度比较（$\bar{x}\pm s$）

Table 3　Comparison of uterus coefficient，hematocrit and plasma viscosity in four groups of rats

组别	n	子宫系数（mg/g）	红细胞压积（$\times10^{-2}$）	血浆粘度（mPa/s）
正常组	10	2.01±0.45	41.11±1.26	1.45±0.06
模型组	9	3.89±0.19①	56.33±1.79①	1.89±0.09①
阳性对照组	9	3.45±0.11①②	49.15±1.58①②	1.75±0.02①②
连翘提取物组	9	2.21±0.06①②③	43.22±1.11①②③	1.52±0.03①②③
F		17.416	32.425	18.997
P		<0.05	<0.05	<0.05

注：与正常对照组相比，①$P<0.05$；与模型组相比，②$P<0.05$；与阳性对照组相比，③$P<0.05$

2.5　4 组大鼠右侧子宫质量、左侧子宫质量、肿胀度比较　**模型组、阳性对照组、连翘提取物组大鼠右侧子宫质量、左侧子宫质量高于正常组，差异有统计学意义（$P<0.05$）；阳性对照组、连翘提取物组大鼠右侧子宫质量、左侧子宫质量、肿胀度低于模型组，差异有统计学意义（均$P<0.05$）；连翘提取物组大鼠右侧子宫质量、左侧子宫质量、肿胀度低于阳性对照组，差异有统计学意义（均 $P<0.05$）。见表 4。**

(C)1994-2022 China Academic Journal Electronic Publishing House. All rights reserved.　http://www.cnki.net

表 4 4 组大鼠右侧子宫质量、左侧子宫质量、肿胀度比较($\bar{x}\pm s$)

Table 4 Comparison of right uterus quality, left uterus quality and swelling degree of four groups of rats

组别	n	右侧子宫质量(g)	左侧子宫质量(g)	肿胀度(g)
正常组	10	0.21±0.02	0.26±0.01	-
模型组	9	0.45±0.02①	0.31±0.02	0.16±0.01①
阳性对照组	9	0.37±0.03①②	0.29±0.01	0.11±0.02①②
连翘提取物组	9	0.25±0.04①②③	0.27±0.02	0.09±0.01①②③
F		39.176	10.511	22.853
P		<0.05	<0.05	<0.05

注:与正常对照组相比,①$P<0.05$;与模型组相比,②$P<0.05$;与阳性对照组相比,③$P<0.05$

3 讨论

慢性盆腔炎属于增生性炎症,其随着病程的变化而加重,是临床上难以解决的问题,也是今年来需要解决的重要问题[5-6]。慢性炎症黏连的形成与多种因素有关,在正常生理状态下,腹膜间皮细胞纤维蛋白原与蛋白溶解之间处于平衡状态,若此关系遭到破坏,导致纤维蛋白的不断增加,溶解障碍,进而形成纤维蛋白性黏连[7]。盆腔炎主要累及子宫、输卵管、卵巢和周围结缔组织等,并可波及盆腹腔腹膜的炎症的总称,因此其治疗准确性不高,其还会由性传播疾病感染、产后、流产后以及妇科手术后细菌进入创面感染而得病,发病可局限于一个部位、几个部位或整个盆腔,因此延误治疗有可能导致盆腔炎后遗症的发生[8-9]。

连翘提取物含有连翘苷、连翘脂素、松脂素-β-D-葡萄糖苷、异连翘脂苷等化学成分,其中连翘质感 A、芦丁等被证实具有抗炎疗效,属于连翘提取物的抗炎有效成分[10]。连翘提取物收载与《药典》中,其主要应用于临床和食品保鲜中,并没有明确的实验证实连翘与抗炎作用一致。孙佳玮等[11]研究表明,连翘根醇提物(FSEER)可以显著提高小鼠机体的免疫功能,恢复由 Cy 诱导的小鼠免疫抑制状态。在本文研究中显示,连翘提取物组大鼠血浆免疫球蛋白含量均高于阳性对照组和模型组,此结果说明,连翘提取物还能够有效改善大鼠机体的免疫功能,效果较好。有学者研究发现[12],将连翘干燥果实的甲醇提取液、正己烷萃取甲醇提取液后所得到的正己烷可溶物和水可溶物分别进行真空冷冻干燥,通过小鼠毛细血管通透性、扭体反应、足趾肿胀和肉芽肿实验表明,连翘甲醇提取物和正己烷可溶物具有消炎和镇痛作用。在本文研究中,连翘提取物组大鼠子宫系数、红细胞压积、血浆粘度低于阳性对照组和模型组,此结果说明,连翘提取物能够改善大鼠的子宫系数和红细胞压积,效果较为突出。武明红等[13]研究表明,慢性盆腔炎患者采用物理疗法与中药连翘炎宁合剂联合进行治疗,可以减少药物不良反应,迅速控制炎症反应,缩短用药时间,提高治疗效果。在本研究中还发现,连翘提取物组大鼠右侧子宫质量、左侧子宫质量、肿胀度低于阳性对照组,此结果说明,连翘提取物能够促进子宫恢复,使各方面恢复正常。与刘玉玲等[14]研究结果一致。

ICAM-1 属于免疫球蛋白家族的一员,其分布交广,且在组织局部炎症炎症中发挥着重要作用。ICAM-1 的生理作用不仅能够调节细胞,还能够参与细胞的信号转导、细胞的生长移行、肿瘤转移的浸润等一系列的生理和病理发展的过程[15-16]。其还能够介导白细胞的稳定、粘附和穿越内皮细胞,能够通过白细胞的激活,而释放出大量的炎性介质[17]。岳秀永等[9]研究表明,当归四逆汤有治疗慢性盆腔炎的作用,作用机理与改善慢性盆腔炎大鼠血流动力学状况,调节粘连相关免疫分子 ICAM-1、FGF-2 的表达有关。在本研究中发现,连翘提取物组大鼠体内 ICAM-1、TLR4、NF-kB 均低于阳性对照组和模型组,此结果说明,连翘提取物能够有效改善慢性盆腔炎大鼠的炎性因子水平,减缓病情进展。INF-γ 具有抗增殖、免疫抑制及抗纤维化活性,能够有效抑制转化生长因子的合成以及成纤维细胞的增殖,是一种重要的抗纤维化因子[18]。当抗纤维化因子水平较高时,会直接阻止过度纤维化的过程,使组织结构损伤后能够适当的修复,进而不会导致盆腔组织的过度黏连。在本文中,连翘提取物组大鼠体内 INF-γ 水平高于阳性对照组和模型组,此结果说明,连翘提取物能够有效提高慢性盆腔炎大鼠体内 INF-γ 水平,能够有效阻止纤维化的发展。与张志鹏等[19]研究结果一致。

4 结论

连翘提取物能够有效缓解大鼠子宫黏连,改善体内炎症反应及血流病变异常,其机制可能与子宫组织 ICAM-1、INF-γ 表达有关。

【参考文献】

[1] 王昕雯,张慕玲. 3 种奥硝唑用药方案治疗慢性盆腔炎的疗效与安全性比较[J]. 中国药房,2017,28(33):4664-4667.

[2] 宋霞. 中医辨证施护对输液式中药保留灌肠治疗慢性盆腔炎患者心理状态及临床疗效的影响[J]. 护士进修杂志,2019,34(9):834-836.

[3] 俞燕丽,傅睿. 连翘提取物通过 PI3K-AKT-mTOR 信号通路逆转结肠癌氟尿嘧啶耐药细胞株的研究[J]. 新中医,2019,51(5):27-30

[4] 王新斌,薛淑萍,马睿玲,等. 清热祛瘀方对慢性盆腔炎模型大鼠血流动力学及免疫分子的 13 影响[J]. 中医研究,2017,30(9):64-68.

(C)1994-2022 China Academic Journal Electronic Publishing House. All rights reserved. http://www.cnki.net

[5] 吕耀中,宗绍波,李芳,等. 散结镇痛胶囊对慢性盆腔炎大鼠抗炎及抗纤维化研究[J]. 中草药,2019,50(20):5011-5017.

[6] BUGG C W, TAIRA T. Pelvic Inflammatory Disease: Diagnosis And Treatment In The Emergency Department[J]. Emerg Med Pract,2016,18(12):1-24.

[7] CHIANG B J, KUO H C, LIAO C H. Can Botulinum Toxin A Still Have a Role in Treatment of Lower Urinary Tract Symptoms/Benign Prostatic Hyperplasia Through Inhibition of Chronic Prostatic Inflammation[J]. Toxins (Basel), 2019, 11(9): 547.

[8] 谢紫烨,黄政海,俞婵娟,等. 康妇消炎栓治疗慢性盆腔炎模型大鼠药效学研究[J]. 中成药,2018,40(12):2747-2750.

[9] 岳秀永,秦建设,方应权,等. 表没食子儿茶素没食子酸酯对慢性盆腔炎大鼠子宫组织的影响[J]. 中成药,2018,40(5):1182-1184.

[10] PAN L, MA X K, ZHAO P F, *et al*. Weeping forsythia extract alleviates dexamethasone-induced oxidative injury of breast muscles in broilers[J]. Animal, 2019,13(11):2660-2668.

[11] 孙佳玮,戴素丽,李磊,等. 连翘根提取物(FSEER)对化疗后小鼠骨髓和免疫抑制的影响[J]. 中国免疫学杂志,2015,(5):625-628.

[12] 陈诚. 连翘提取物可有效缓解力竭运动导致的骨骼肌组织氧化损伤[J]. 基因组学与应用生物学,2018,37(1):53-58.

[13] 武明红,张海燕,路燕. 连翘炎宁合剂联合物理疗法对慢性盆腔炎患者治疗效果的影响[J]. 中外女性健康研究,2019,(3):82,84.

[14] 刘玉玲,汪栋材,陈博勉,等. 王孟庸运用当归连翘赤小豆汤加味验案举隅[J]. 中国中医药信息杂志,2019,26(6):118-120.

[15] BUFFONE A J R, ANDERSON N R, HAMMER D A. Human Neutrophils Will Crawl Upstream on ICAM-1 If Mac-1 Is Blocked[J]. Biophys J,2019,117(8):1393-1404.

[16] LENNARTZ F, SMITH C, CRAIG A G, *et al*. Structural insights into diverse modes of ICAM-1 binding by Plasmodium falciparum-infected erythrocytes[J]. Proc Natl Acad Sci U S A, 2019,116(40):20124-20134.

[17] BENEDICTO A, HERRERO A, ROMAYOR I, *et al*. Liver sinusoidal endothelial cell ICAM-1 mediated tumor/endothelial crosstalk drives the development of liver metastasis by initiating inflammatory and angiogenic responses[J]. Sci Rep,2019,9(1): 13111.

[18] HU H, CHEN E, LI Y, *et al*. Effects of Arsenic Trioxide on INF-gamma Gene Expression in MRL/lpr Mice and Human Lupus[J]. Biol Trace Elem Res,2018,184(2):391-397.

[19] 张志鹏,高升,任存霞. 当归芍药散对慢性盆腔炎模型大鼠分子免疫调控的影响[J]. 中华中医药学刊,2015,33(11):2684-2686.

(收稿日期:2019-11-26;修回日期:2021-10-09;编辑:王小菊)

(上接第189页)

[18] YANG C, YAO C, TIAN R, *et al*. miR-202-3p Regulates Sertoli Cell Proliferation, Synthesis Function, and Apoptosis by Targeting LRP6 and Cyclin D1 of Wnt/β-Catenin Signaling [J]. Mol Ther Nucleic Acids,2019,14:1-19.

[19] LV M Q, ZHOU L, GE P, *et al*. Over-expression of hsa_circ_0000116 in patients with non-obstructive azoospermia and its predictive value in testicular sperm retrieval[J]. Andrology, 2020,8(6):1834-1843.

[20] REN S, LIN P, WANG J, *et al*. Circular RNAs: Promising Molecular Biomarkers of Human Aging-Related Diseases via Functioning as an miRNA Sponge[J]. Mol Ther Methods Clin Dev,2020,6(18):215-229.

[21] LIN W, LIU H, TANG Y, *et al*. Circular RNAs: Promising Molecular Biomarkers of Human Aging-Related Diseases via Functioning as an miRNA Sponge [J]. Mol Cell Biochem,2021, 476(1):109-123.

[22] AGARWAL V, BELL G W, NAM J W, *et al*. Predicting effective microRNA target sites in mammalian mRNAs [J]. Elife, 2015,4: e05005.

[23] LIN W, LIU H, TANG Y, *et al*. Circular RNAs: Promising Molecular Biomarkers of Human Aging-Related Diseases via Functioning as an miRNA Sponge [J]. RNA,2004, 10(10): 1507-1517.

[24] QUILLET A, SAAD C, FERRY, *et al*. Improving Bioinformatics Prediction of microRNA Targets by Ranks Aggregation [J]. Front Genet,2020,10:1330.

[25] KEN SAKURAI, KEI MIKAMOTO, MAKOTO SHIRAI, *et al*. MicroRNA profiling in ethylene glycol monomethyl ether-induced monkey testicular toxicity model [J]. J Toxicol Sci,2015, 40(3):375-382.

[26] WANG Y, ZANG R K, DU Y N. HSA_CIRC_0004050 on proliferation and apoptosis of A549 cells through ERK/JNK signaling pathway [J]. J Biol Regul Homeost Agents,2020, 34(6): 2037-2047.

[27] LI H, LI L, LIN C, *et al*. Decreased miR-149 expression in sperm is correlated with the quality of early embryonic development in conventional in vitro fertilization [J]. Reprod Toxicol, 2021,101:28-32.

(收稿日期:2021-04-18;修回日期:2021-11-01;编辑:王小菊)

(C)1994-2022 China Academic Journal Electronic Publishing House. All rights reserved. http://www.cnki.net
